# Selected abstracts from the 26th Conference of the European Cell Death Organization (ECDO): Cell death in disease — from small molecules to translational medicine

**DOI:** 10.1038/s41420-018-0128-4

**Published:** 2019-01-03

## Abstract

**Sponsorship:** Publication of this supplement was sponsored by the Medical Research Council (MRC). All content was reviewed and approved by the European Cell Death Organization (ECDO) Committee, which held full responsibility for the abstract selections.

## ECDO 01 Prevention of apoptosis progression by Deoxyribonuclease I inactivation through Endonuclease G-induced alternative splicing of its mRNA

### Dmitry D. Zhdanov^1,2,^, Yulia A. Gladilina^1^, Vadim S. Pokrovsky^1,2^, Dmitry V. Grishin^1^, Marina V. Pokrovskaya^1^, Svetlana S. Alexandrova^1^, Anna A. Plyasova^1^, and Nikolay N. Sokolov^1^

#### ^*1*^*Institute of Biomedical Chemistry, Moscow, Russia*; ^*2*^*Peoples Friendship University of Russia (RUDN University), Moscow, Russia*

Irreversibility of apoptosis is being ensured by apoptotic endonucleases that act cooperatively to fragment DNA at the latest stages of cell death. Potential regulatory links between the endonucleases remain to be investigated. Previously it was shown that Deoxyribonuclease I (DNase I) inactivation is caused by alternative splicing (AS) of DNase I pre-mRNA by exon 4 skipping, which occurred in response to Endonuclease G (EndoG) overexpression in human and murine T cells. The aim of the work was to determine the role of EndoG in the regulation of DNase I mRNA AS and modulation of its enzymatic activity. A strong correlation was identified between EndoG expression levels and DNase I splice variants in human lymphocytes. EndoG overexpression in CD4+ T lymphocytes down-regulated mRNA of active full-length DNase I variant and up-regulated the non-active spliced variant, which acts as dominant-negative. DNase I AS was induced by EndoG translocation from mitochondria into nuclei during apoptosis development. DNase I spliced variant was induced by recombinant EndoG or by incubation with EndoG-digested cell RNA in vitro system with isolated cell nuclei. Using antisense DNA oligonucleotides, we identified a 72-base segment which spans through the adjacent parts of exon 4 and intron 4 and responsible for the AS. DNase I-positive CD4+ T cells with overexpressed EndoG demonstrated decreased progression of bleomycin-induced apoptosis. Therefore, EndoG is an endonuclease with the unique ability to inactivate another endonuclease, DNase I and to modulate apoptosis development.

## ECDO 02 Exploiting Metabolic Reprogramming to OXPHOS in Oncogene Addicted Reclacitrant Cancers

### Hirpara Jayshree^1^, Jie Qing^1^, Andrea Wong^1^, Kumi Higuchi^2,3^, Takeshi Tsunoda^3^, Marie-Véronique Clément^1^, Boon Cher Goh^1,4^ and Shazib Pervaiz^1,4^

#### ^*1*^*National University of Singapore, Singapore*; ^*2*^*Fujii Memorial Research Institute, Japan;*^*3*^*Otsuka Pharmaceutical Co., Ltd., Japan;*^*4*^*National University Cancer Institute, NUHS, Singapore*

According to the Warburg phenomenon, cancer cells switch their major energy source from mitochondrial oxidative phosphorylation (OXPHOS) to glycolysis, thereby resulting in increased lactate production. Interestingly, there is emerging evidence to indicate a remarkable plasticity between these two cellular ATP sources and the switch to one or the other is a function of mitochondrial redox environment. While, glycolysis might be the trigger for cellular transformation and initiation of carcinogenesis, cancer cells that are addicted to oncogene-induced signaling and resistance to targeted therapies exhibit a significantly enhanced reliance on OXPHOS. As such, specific targeting of OXPHOS presents itself as an attractive strategy against aggressive and refractory cancers. Using two different models of oncogene addicted cancers, i.e non small cell lung carcinoma (NSCLC) cell line HCC827 and its gefitinib resistant clone and malignant melanoma cell line A375 and its vemurafanib resistant clone, we provide evidence that the TKI-resistant clones harbour significantly higher OXPHOS activity. Significant upregulation of the mitochondrial electron transport chain complex I protein (NDUFA9) together with increased complex I activity and higher mitochondrial DNA copy number are observed in both the resistant clones. Notably, a significant increase in STAT3 activity is detected in oncogene addicted cancer cells, and the increased mitochondrial oxygen consumption and complex I activity could be significantly inhibited by a novel small molecule inhibitor of STAT3, OPB-51602. The latter is shown to be an effect that might be independent of the STAT3 inhibitory activity of the small molecule compound. Most importantly, the novel complex I inhibitor elicited strong anti-tumor activity in three different murine models of carcinogenesis as well as in cancer patients treated with the novel small molecule. Taken together, these data demonstrate that oncogene addicted recalcitrant cancers rewire their metabolism to one that is predominantly OXPHOS dependent, and thus highlight an exploitable vulnerability to pharmacological inhibitors of OXPHOS.

## ECDO 03 Unexpected overlapping roles of multiple caspases and programmed cell death pathways in the response to bacterial infection

### Ranja Salvamoser^1,2^, Paul G Whitney^2,3^, Marcel Doerflinger^1,2^, Sammy Bedoui^2,3^, Andreas Strasser^1,2^, Clare Bryant^4^, Marco J Herold^1,2^

#### ^*1*^*The Walter and Eliza Hall Institute of Medical Research, 1G Royal Parade, Parkville, Victoria 3052, Australia;*^*2*^*University of Melbourne, Parkville, Victoria 3050, Australia;*^*3*^*Peter Doherty Institute for Infection and Immunity, Melbourne, Victoria, Australia;*^*4*^*The University of Cambridge, Cambridge, Cambridgeshire, UK*

Large multi-protein complexes known as inflammasomes control pathogen invasion and induce inflammatory cell death known as pyroptosis via the activation of caspases, a family of aspartate-specific cysteinyl proteases.

*Salmonella* Typhimurium triggers pyroptosis by activating caspases -1 and -11, in part via the NLRP3 and NLRC4 inflammasomes. Previous reports implicated also caspase-8 (and possibly other caspases) in the inflammatory response. Additionally, there is evidence for a high level of functional overlap between different cell death pathways in the cellular response to pathogens. We tested these hypotheses by generating mice deficient for multiple caspases, and also lacking RIPK3 an essential mediator of necroptotic cell death to prevent the embryonic lethality caused by the loss of caspase-8.

Interestingly, primary macrophages lacking caspases-1/11/12 and also caspase-8 as well as RIPK3 were highly resistant in vitro to infection with the Salmonella Typhimurium Sl1344 strain, even at MOIs as high as 500. This was surprising, as previous reports have shown that caspase-8/RipK3 double-deficient macrophages showed only a minor delay in Salmonella induced cell death compared to WT cells.

Importantly, when infecting the Caspase-1/11/12/8/RipK3 quintuple deficient mice with the vaccine strain BRD509, these mice were not able to clear the bacteria from their livers and spleens. Interestingly, the lack caspase-12 appears to affect the ability of bacteria to disseminate from the gall bladder; since higher log numbers of salmonella were observed in the gall bladders of the Caspase-1/11/12 triple knockout mice as well as the Caspase-1/11/12/8/RipK3 quintuple knockout mice when compared to Caspase-1/11 double deficient animals and wildtype controls. This might suggest a role for caspase-12 in the release of bacteria from the gall bladder.

Collectively, these findings provide clear evidence that multiple cell death pathways and hence the caspases involved in these processes show functional overlap in the response to infection with bacteria and possibly other pathogens. Moreover, our studies suggest a novel role for caspase-12 in the dissemination of bacteria from the gall bladder.

## ECDO 04 Cancer cells employ nuclear caspase-8 to overcome the p53-dependent G2/M checkpoint through cleavage of USP28

### Ines Müller^1^, Elwira Strozyk^1^, Sebastian Schindler^1^, Greta Del Mistro^1^, Stefan Beissert^1^, Htoo Zarni Oo^2,3^, Thomas Sauter^4^, Philippe Lucarelli^4^, Sebastian Raeth^5^, Angelika Hausser^5^, Nader Al Nakouzi^2,3^, Beibei Zhai^2,3^, Ladan Fazli^2,3^, Martin E. Gleave^2,3^, He Liu^6^, Hans-Uwe Simon^6^, Henning Walczak^7^, Douglas R. Green^8^, Jiri Bartek^19,10^, Mads Daugaard^2,3^& Dagmar Kulms^1^

#### ^*1*^*TU-Dresden, 01307 Dresden, Germany;*^*3*^*University of British Columbia, Vancouver, BC, Canada V5Z 1M9;*^*3*^*Vancouver Prostate Centre, Vancouver, BC, Canada V6H 3Z6;*^*4*^*University of Luxembourg, 1511 Luxembourg;*^*5*^*University of Stuttgart, 70569 Stuttgart, Germany;*^*6*^*University of Bern, 3010 Bern, Switzerland;*^*7*^*University College London, London WC1E 6DD, UK;*^*8*^*St. Judes Children´s Research Hospital, Memphis, TN 38105, USA;*^*9*^*Danish Cancer Society Research Center, Copenhagen, DK-2100, Denmark.;*^*10*^*Karolinska Institute, Stockholm, 171 77, Sweden*

Cytosolic caspase-8 is a mediator of death receptor signaling. While caspase-8 expression is lost in some tumors, it is increased in others, indicating a conditional pro-survival function of caspase-8 in cancer. Here we show that tumor cells employ DNA damage-induced nuclear caspase-8 to override the p53-dependent G2/M cell cycle checkpoint. Caspase-8 is upregulated and localized to the nucleus in multiple human cancers correlating with treatment resistance and poor clinical outcome. Depletion of caspase-8 causes G2/M arrest, stabilization of p53, and induction of p53-dependent intrinsic apoptosis in tumor cells. In the nucleus, caspase-8 cleaves and inactivates the ubiquitin-specific peptidase 28 (USP28), preventing USP28 from de-ubiquitinating and stabilizing wildtype p53. This results in *de facto* p53 protein loss, switching cell fate from apoptosis towards mitosis. In summary, our work identifies a non-canonical role of caspase-8 exploited by cancer cells to override the p53-dependent G2/M cell cycle checkpoint.

## ECDO 05 Respective place of venetoclax and MCL1 BH3 mimetics in Multiple Myeloma treatment and mechanism of action of the BH3 mimetics

### Amiot M^1,2^, Maiga S^1,2^, Tessoulin B^1,2^, Seiller C^1^, Bourcier J^1^, Bonnet A^1,2^, Descamps G^1^, Touzeau C^1,2^, Moreau P^1,2^, Pellat-Deceunynck C^1^, Gomez-Bougie P^1,2^

#### ^*1*^*CRCINA, INSERM, CNRS, Université d’Angers, Université de Nantes, France;*^*2*^*CHU de Nantes, France*

Targeting anti-apoptotic proteins of the BCL2 family by BH3 mimetics is a new promising therapeutic approach in Multiple Myeloma (MM). In the present study, we used a BH3 mimetic toolkit that includes venetoclax, A1155463 and A1210477, which target BCL2, BCLXL and MCL1 respectively to define dependencies/co-dependencies in a large cohort of 60 myeloma patients (21 at diagnosis and 39 at relapse). Alternatively, MCL1 dependency was confirmed using the S63845 MCL1 inhibitor in MM patient samples. Mononuclear bone marrow/blood cells were treated overnight with the respective BH3 mimetic and cell death was specifically measured in the tumor cell population. Primary MM cells dependencies were stratified using PCA analysis in three groups as highly dependent, intermediately dependent or not dependent. Our study demonstrated that half of patients at diagnosis were BCL2 dependent while only 10% were BCLXL dependent. The dependence on BCL2 or BCLXL was not significantly different between samples at diagnosis and relapse. Strikingly, we found that the MCL1 dependency was 33% at diagnosis while it was 69% at relapse, suggesting a significant increase in MCL1 dependency during the disease progression (p=0.01). Besides, 36% of overall patients showed co-dependencies on BCL2/MCL1. We also identified primary MM cells that did not depend on any of the three pro-survival molecules, both at diagnosis and relapse.

Among this cohort of MM patients, 47 samples were further analyzed for the presence of recurrent translocations (t(11;14), t(6;14), t(4;14) and t(14;16)) allowing the analysis of dependencies in the different subgroups; these recurrent translocations lead to the over-expression of CCND1, CCND3, MMSET and MAF oncogenes respectively. We found that BCL2 dependency was significantly higher in t(11;14) subgroup (83%) compared to all other subgroups (20%, p=0.008) as previously demonstrated by our team^1^ and confirmed by the result of the clinical trial evaluating venetoclax in relapsed/refractory MM^2^. We also confirmed the BCL2/BCLXL mRNA ratio as a valuable biomarker to define BCL2 dependence (p=0.0001). At diagnosis, MCL1 dependency was absent in patients not harboring the common recurrent translocations while at relapse 6 out 9 patients not harboring the recurrent translocations were MCL1 dependent, indicating an increase of MCL1 dependency at relapse in this group (p=0.03).

In conclusion, our study highlights the potential clinical use of BH3 mimetics in MM treatment guided by the practical ex-vivo testing of myeloma cell dependencies using the BH3 toolkit. This strategy could be used to identify the respective and tailored use of venetoclax, MCL1 BH3 mimetic or their combination in myeloma treatment.

## ECDO 06 Second mitochondrial-derived activator of caspases (SMAC) mimetic LCL-161 and TNF-related apoptosis-inducing ligand (TRAIL) induce apoptosis through a kinase-independent RIP1 function in sensitive breast cancer cells

### Christian Holmgren^1^, Ellen Sunström Thörnberg^1^, Victoria Granqvist^1^, Christer Larsson^1^

#### ^*1*^*Lund University, Lund, Sweden*

Second mitochondrial-derived activator of caspases (SMAC) is a pro-apoptotic protein that facilitates apoptosis through binding inhibitor of apoptosis proteins (IAPs), resulting in disinhibition of caspases and stimulation of pro-apoptotic signaling. Based on the ability of SMAC to inhibit IAPs and stimulate apoptosis, a class of compounds called SMAC mimetics have been developed that mimic the IAP-binding functions of SMAC. They facilitate cell death in cancer cells and are currently investigated in clinical trials as anti-cancer agents. However, a majority of cancer cells show resistance to SMAC mimetics. This has led to the pursuit of other compounds that can be used in combination with SMAC mimetics to induce death of cancer cells.

Our objective is to study if SMAC mimetics in combination with TNF-related apoptosis-inducing ligand (TRAIL) could be used to induce cell death in breast cancer cells that are resistant to SMAC mimetics.

The efficiency of the SMAC mimetic LCL-161 to induce cell death alone or in combination with TRAIL was studied by WST-1 assay on breast cancer cell lines and cell death was measured using annexin-V/propidium iodide labeling and flow cytometry. Western blotting and inhibitors against cell death mediators was used to determine the mode of cell death in the cell lines and this was complemented with siRNA-mediated downregulation of proteins in cell death pathways. Cell death complex formation was analyzed using co-immunoprecipitation.

We found that the estrogen receptor (ER)-negative MDA-MB-468 and the ER-positive CAMA-1 cell lines are insensitive to LCL-161 as a single agent and require high TRAIL concentrations for cell viability to be decreased.

Our data shows that sensitivity to TRAIL can be increased by co-treatment with SMAC-mimetic LCL-161 in the MDA-MB-468 and CAMA-1 breast cancer cell lines. In addition, we show that the cell death is linked to association of caspase-8 with RIP1 and that RIP1 mediates the apoptotic response independently of its kinase activity.

## ECDO 07 SMAC mimetic LCL-161 in combination with TRAIL induce an inflammatory response in MCF-7 breast cancer cells

### Victoria Granqvist^1^, Christian Holmgren^1^, Christer Larsson^1^

#### ^*1*^*Lund University, Lund, Sweden*

Breast cancer constitutes around 25 percent of all new cancer cases among females and is therefore the most common form of cancer affecting women. Generally the prognosis is good but there are still a substantial number of patients that suffer from relapse. Therefore, there is a need of novel treatments. Second mitochondrial-derived activator of caspases (SMAC) mimetics are a class of small molecule compounds that mimic the function of the pro-apoptotic protein SMAC and were developed to facilitate cell death. SMAC mimetics are usually combined with other agents to induce apoptosis. When treating MCF-7 breast cancer cells with SMAC mimetic LCL-161 together with TNF-related apoptosis-inducing ligand (TRAIL), we have found that the cells change phenotype rather than undergo apoptosis.

Our objectives are to study the underlying mechanisms of phenotypic changes induced by SMAC mimetic and TRAIL in breast cancer cells, focusing on the roles of interferon and NF-κB signaling.

The effects of SMAC mimetic and TRAIL treatment on gene expression were analyzed by microarray, RNA-sequencing and qRT-PCR. Western blot was used to determine activation of cell signaling pathways.

signaling, two pathways associated with inflammation, were upregulated in MCF-7 breast cancer cells after 24 h of treatment with LCL-161 and TRAIL. Western blot confirmed that these pathways are active, as we could detect an increase in the non-canonical NF-κB transcription factor p52 as well as phosphorylation of STAT1. After 96 h of treatment + 3 d after re-seeding the cells, and thereby removing treatments, genes related to estrogen receptor signaling were downregulated. Moreover, we found a shift towards a more basal-like phenotype since the majority of the downregulated genes are lower in basal-like tumors and the majority of the upregulated genes are higher in basal-like tumors, when compared to luminal tumors. Results from RNA sequencing and qRT-PCR analysis, where the cells were treated with zVAD-fmk in addition to LCL-161 and TRAIL, show that some genes are caspase-dependent. More specifically, interferon-related genes were potentiated after inhibition of caspase activity, whereas genes associated with NF-κB signaling were dampened. The protein level of phosphorylated STAT1 was also found to be enhanced after pre-treatment with zVAD-fmk.

Our results from RNA analysis show that treatment with SMAC mimetic LCL-161 and TRAIL induces an inflammatory response in luminal MCF-7 breast cancer cells. Several of these genes are known to be related to interferon- and NF-κB-signaling. Induction of some of these genes was found to be caspase-dependent. Elevated protein levels of phosphorylated STAT1 and p52 indicate that both interferon and non-canonical NF-κB signaling pathways are activated. In addition, the combination treatment induces a shift towards a more basal-like phenotype.

Acknowledgment: VG and CH contributed equally.

## ECDO 08 Ultrastructural features of apoptosis of cultured bone marrow cells

### G.V. Fedotovskikh^1^,G.M. Shaimardanova^1^, M.B. Askarov^1^

#### ^1^*JSC "National Scientific Medical Center” Astana, Kazakhstan*

Mechanisms of functioning of hemopoietic and mesenchymal bone marrow cells, widely used in regenerative medicine, are still on the search path. Interesting data on the pronounced therapeutic effect of apoptotic bodies and microvesicles of apoptotic mesenchymal stem cells (Petrenko A.E. et al., 2017) led us to a more careful study of the morpho-functional state of cultured bone marrow cells in the state of apoptosis.

The research objective was to study the ultrastructure of apoptosis of cultured bone marrow cells. Electron microscopy was used to study the 24-hour pre-cultured hematopoietic and cultured mesenchymal cells (MSC) of the bone marrow of 62 patients with non-infectious chronic pathology. The cells were fixed in a 2.5% solution of glutaraldehyde with postfixation at 1% 0s04, conducted according to a conventional method and enclosed in epon. Semi-thin and ultrathin sections were obtained on the ultramicrotome "Leica" (Austria). Semi-thin sections were stained with methylene blue - azur II and basic fuchsin by Humphrey C., Pittman F. (1974). Ultrathin sections were contrasted with uranyl acetate and lead citrate by Reynolds. Observation and shooting of ultrathin sections was carried out on an electron microscope Libra 120 (C.Zeiss). At a high-resolution light-optical level (semi-thin sections) in the presence of single cultured cells in the state of apoptosis with the formation of apoptotic bodies, a certain part of cultured GSK and MSC contained an osmiophilic nucleus of round and crescent shape without fragmentation. The cell sizes and cytoplasm density were not changed. The nuclei had a central and peripheral location. Electron microscopy noted a sharp condensation of nuclear chromatin with a dense arrangement of ribonucleoprotein complexes. Fragmentation of small areas of condensed chromatin located under the nuclear membrane was rare. Small and large euchromatin sections were marked at the periphery of the nucleus, whose low electron density determined the crescent shape of the nucleus on semi-thin sections. In the field of nuclear membranes, vesicles were formed. In the complete absence of a nuclear envelope, ribonucleoprotein complexes were located freely in the hyaloplasm among the organelles and numerous endosomes. The mitochondria had a condensed type of structure. The outer cytoplasmic membrane was not altered. An electron microscopic examination revealed ultrastructural signs of preapoptosis in a certain part of cultured GSK and MSC of the bone marrow. The obtained data may be related to regulatory RNA that determine the state of chromatin and play an important role in the transfer of genetic information by stem cells (A. Tkachuk, 2017).

## ECDO 09 Expansion of human NK cells for the treatment of multiple myeloma

### Chantal Reina-Ortiz^1^, Taylor Ewing^1^, Alfonso Serrano del Valle^1^, Joaquin Marco-Brualla^1^, Isabel Izquierdo^2^, Gemma Azaceta^3^, Luis Palomera^3^, Isabel Marzo^1^, Javier Naval^1^, Alberto Anel^1^

#### ^*1*^*University of Zaragoza, Zaragoza, Spain;*^*2*^*University Hospital Miguel Servet, Zaragoza, Spain;*^*3*^*University Clinic Hospital Lozano Blesa, Instituto Aragonés de Ciencias de la Salud (IACS), Zaragoza, Spain*

Chemotherapeutic and radiotherapeutic approaches to cancer treatment have a limited ability to discriminate between healthy and malignant cells. To increase specificity and decrease damage to healthy tissue, cancer immunotherapy is leading a paradigm shift in research and treatment development. Natural killer cells are part of our immune defense against transformed cells and are a promising option for use against cancer cells. For a viable treatment, human allogenic NK cells are expanded and activated before use on target cells. The purpose of this study was to test the efficacy of expanded natural killer cells (eNKs) as a treatment against multiple myeloma (MM) cell lines and MM patient bone marrow aspirates.

We produced expanded allogenic NK cells using activation with feeder cells and cytokines. The cytotoxic effect of eNKs was studied alone and in combination with daratumumab, an anti-CD38 monoclonal antibody approved for use in MM patients.

In MM cell lines, eNKs were highly cytotoxic with 20-70% specific cell death, depending on the cell line. For the MM patient samples, when eNKs were harvested during the optimum expansion period, they produced a cytotoxic effect against the MM cells. Daratumumab alone was also effective against some of the patient samples. However, when combined, eNKs and daratumumab produced a greater cytotoxicity than when used separately.

Our data supports the possible use of eNKs in combination with daratumumab therapy against multiple myeloma in further trials.

## ECDO 10 Novel BRET-based proximity biosensor for the study mitochondria-ER contact sites

### Hector Flores-Romero^1^, Vanessa Hertlein^1^, Kushal K. Das^1^, Sebastian Fischer^2^, Michael Heunemann^1^, Klaus Harter^1^, Ana J. García-Sáez^1^

#### ^*1*^*University of Tübingen, Germany;*^*2*^*University of Heidelberg, Germany*

The contacts between the endoplasmic reticulum (ER) and mitochondria play a key role in cellular functions like the exchange of lipids and calcium between both organelles, as well as in apoptosis and autophagy signaling. The molecular architecture and spatiotemporal regulation of these distinct contact regions remain obscure and there is a need for new tools that enable tackling these questions. Here we present a new Bioluminescence Resonance Energy Transfer (BRET)-based biosensor for the quantitative analysis of distances between ER and mitochondria that we call MERLIN (Mitochondria ER Length Indicator Nanosensor). The main advantages of MERLIN compared to available alternatives are that it does not rely on the formation of artificial physical links between the two organelles, which could lead to artifacts, and that it allows to study contact site reversibility and dynamics. We show the applicability of MERLIN by characterizing the role of the mitochondrial dynamics machinery on the contacts of this organelle with the ER.

## ECDO 11 Birinapant augments the efficacy of isolated limb perfusion in an animal model of extremity soft tissue sarcoma

### Kunzah Jamal^1^, Henry Smith^1^, Tencho Tenev^1^, Joan Kyula^1^, Victoria Roulstone^1^, Kevin Harrington^1^, and Pascal Meier^1^

#### ^*1*^*Institute of Cancer Research, Mary-Jean Mitchell Green Building, Chester Beatty Laboratories, Fulham Road, London SW3 6JB, UK*

The addition of tumour necrosis factor (TNF) has significantly improved the efficacy of Isolated limb perfusion (ILP) for the treatment of extremity soft tissue sarcomas (ESTS). However, in most cases metastases occur limiting disease free survival. Birinapant is a bivalent SMAC mimetic (SM), which synergises with TNF and induces immunogenic cell death. Here we show that the addition of BIR to the established ILP regime significantly increases the survival of an orthotopic model of ESTS. The rat sarcoma cells, BN175, are moderately sensitive to the combination of TNF, melphalan and SM in vitro. Mechanistically, the death induced by TNF and SM is RIPK1 and capsase-8 dependent. FACS analysis of the SM treated tumours reveals increased FOXP3-expressing regulatory T (Treg) cells infiltration compared to the standard of care suggesting that the reoccurrence of disease could be due to an immune-suppressing environment. In fact, immune checkpoint blockage post SM treatment further increases survival compared to the standard of care. Furthermore, tumour cell lines derived from the SM/ILP treated cohort remain sensitive to SM in vitro suggesting that tumour re-occurrence is due to the initial treatment not killing off the tumour population. In accordance, treatment of BN175 cells with a novel TLR3 agonist riboxxol and SM potently kills BN175 in vitro suggesting that in vivo, riboxxol and SM could be applied systemically to treat recurring malignancies and prolong disease free survival.

Acknowledgment: KJ and HS contributed equally. KH and PM serve as Co-senior authors.

Disclosure: The authors declare no competing interests.

## ECDO 12 Pyroptosis features a caspase-1-induced apoptotic program that coincides with induction of GSDMD-driven cell lysis

### Nathalia M. de Vasconcelos^1^, Nina Van Opdenbosch^1,2,^, Hanne Van Gorp^1^, Mohamed Lamkanfi^1,2^

#### ^*1*^*Ghent University, Ghent, Belgium;*^*2*^*Janssen Immunosciences, World Without Disease Accelarator, Janssen Pharmaceutica, Pharmaceutical Companies of Johnson & Johnson, Beerse, Belgium*

Pyroptosis is a lytic programmed cell death mode that is induced by inflammatory caspases, and represents a major mechanism for extracellular release of alarmins and the inflammatory cytokines IL-1β and IL-18. We previously reported on the pyroptotic subcellular changes that are induced by the pore-forming protein gasdermin D (GSDMD), which include a gradual permeabilization of the plasma membrane and mitochondrial decay prior to catastrophic cell lysis. However, understanding of the biochemical mechanisms that underpin pyroptosis is still limited. Here, we show that pyroptosis comprises an underlying apoptotic program that is masked by fast GSMDM-mediated cell lysis. We demonstrate that pyroptosis induction by the NLRP1b and NLRC4 inflammasomes is associated with a biochemical signature of executioner caspase activation. Pyroptotic DEVDase activity in bone marrow-derived macrophages (BMDMs) was associated with the concomitant cleavage of hallmark apoptotic substrates BID, ROCK-I and p23. Genetic deletion of GSDMD unmasked an apoptotic program that proceeded independently of ASC and caspase-8, contrary to inflammasome-induced apoptosis induction in caspase-1-deficient macrophages. Moreover, GSDMD-deficient macrophages lacking the apoptotic executioner caspases 3 and 7 were markedly protected from inflammasome-induced cell death. Based on these findings, we propose that pyroptosis features a caspase-1-induced apoptotic program underneath the parallel induction of GSDMD-driven cell lysis.

Acknowledgement: NMdV and NVO contributed equally.

## ECDO 13 Apoptosis regulation in ischemic penumbra after photothrombotic stroke

### Uzdensky A.B.^1^, Demyanenko S.V.^1^

#### ^*1*^*Academy of Biology and Biotechnology, Southern Federal University, Rostov-on-Don, 344090, Russia*

Stroke is the leading cause of human death and disability. In the ischemic core vascular occlusion and ATP depletion rapidly, for minutes induce necrosis. During next hours cell damage spreads, and the transition zone (penumbra), in which apoptosis prevails, is formed. Penumbra is potentially salvageable, however, an efficient anti-stroke neuroprotector is still not found. Failure of medications aimed at the first level processes of the injury propagation and penumbra formation (blockers of NMDA receptors and Ca^2+^ channels, antioxidants, anti-apoptotic drugs, etc.) requires the search of agents directed to signaling pathways and transcription factors, which control apoptosis.

Using “The Panorama Antibody Microarray – Cell Signaling” (Sigma-Aldrich) we studied the expression of 224 signaling proteins in the penumbra (1.5 mm width ring around the Ø3mm infarct core in the rat brain cortex) after photothrombotic stroke (a model of ischemic stroke).

Two basic features of cell death regulation in the ischemic penumbra were observed along with other signaling events (regulation and deterioration of the cytoskeleton, vesicular transport, synaptic processes, etc.): (*i*) Simultaneous upregulation in the penumbra of both: apoptotic and anti-apoptotic proteins, so that the cell fate was determined by the balance between these opposite tendencies. (*ii*) Concerted upregulation of proteins that execute apoptosis (caspases 3, 6, 7; Bcl-10, SMAC/DIABLO, AIF, PSR), signaling proteins that regulate different apoptosis pathways (p38, JNK, DYRK1A, neurotrophin receptor p75); transcription factors that control expression of pro-apoptotic proteins (E2F1, p53, c-Myc, GADD153); and proteins, which are normally involved in diverse cellular functions, but stimulate apoptosis in specific situations (NMDAR2a, Par4, GAD65/67, caspase 11). Simultaneously, various anti-apoptotic proteins (Bcl-x, p21/WAF-1, MDM2, p63, PKBα, ERK1, RAF1, ERK5, MAPKAPK2, protein phosphatases 1α and MKP-1, estrogen and EGF receptors, calmodulin, CaMKII, CaMKIV) were over-expressed in ischemic penumbra. Therefore, diverse apoptosis regulation pathways are induced simultaneously in ischemic penumbra from very different initial positions.

These data provide an integral view of neurodegeneration and neuroprotection in the ischemic penumbra. Some of these proteins may serve as potential targets for anti-stroke therapy.

Disclosure: Supported by Russian Science Foundation (#18-15-00110). A.B. Uzdensky was supported by Russian Ministry of Education and Science (Research organization grant #6.4951.2017/6.7).

## ECDO 14 Epigenetic processes in the ischemic penumbra after photothrombotic stroke in the rat cerebral cortex

### Demyanenko S.V.^1^, Dzreyan V.A., Guzenko V.V.^1^, Uzdensky A.B.^1^

#### ^*1*^*Southern Federal University, Rostov-on-Don, Russia*

Stroke is the leading cause of human death and disability. In ischemic stroke vascular occlusion and energy deficit rapidly induce cell necrosis and tissue infarct. However, during next hours the cell damage propagates from the infarct core to the surrounding tissue and forms the transition zone (penumbra), which is potentially salvageable. The mechanisms that regulate protein expression in the penumbra are insufficiently studied. Epigenetic processes such as covalent histone modifications play a pivotal role in global regulation of transcriptional activity of the genome.

To study the epigenetic mechanisms involved in the regulation of protein expression in the ischemic penumbra after photothrombotic stroke (a model of ischemic stroke) in the rat cerebral cortex, we used antibody microarrays that, except other cellular proteins, contain antibodies against 10 proteins involved in epigenetic regulation. Photothrombotic stroke was induced by local laser irradiation of the rat cerebral cortex after i.v. administration of the photosensitizer Rose Bengal, which does not penetrate cells and remains in the bloodstream. Laser irradiation causes oxidative lesion of blood vessels, formation of thrombi, and local tissue infarct. The microarrays were scanned on the GenePix 4100A microarray scanner at 532 and 635 nm. The fluorescence images were subjected to ratio-based normalization. Validation effects was carried out by immunohistochemistry and western blotting.

Photothrombotic stroke reduced the acetylation of lysine 9 and phosphorylation of serine 10 in histone H3 in the ischemic penumbra at 1, 4 or 24 hours after impact. This effect could be first associated with the increased expression of histone deacetylase HDAC1 observed at 1 h after photothrombotic stroke. Later, at 4-24 h, other deacetylases HDAC2 and HDAC4 were also over-expressed that additionally deacetylated histone H3. Histone acetyltransferases HAT1 and PSAF were also up-regulated in the penumbra, but lesser and later (at 4-24 and 24 h, respectively.

Disclosure: Supported by Russian Science Foundation (#18-15-00110). A.B. Uzdensky was supported by Russian Ministry of Education and Science (Research organization grant #6.4951.2017/6.7).

## ECDO 15 Adipose-derived stromal cells of patients with type 2 diabetes mellitus have higher level of autophagy and inflammation compare with normal glucose tolerance patients

### I. Stafeev^1,2,3^, S. Michurina^1,2^, N. Podkuychenko^1,2,3^, I. Sklyanik^3^, A. Panevina^3^, E. Shestakova^3^, K. Yah’yaev^4^, E. Ratner^1,3^, A. Vorotnikov^1,2^, M. Menshikov^1^, Yu. Yashkov^5^, M. Shestakova^3^, Ye. Parfyonova^1,2^

#### ^*1*^*National Medical Research Center for Cardiology, Russia;*^*2*^*Lomonosov Moscow State University, Russai;*^*3*^*National Medical Research Center for Endocrinology, Russia;*^*4*^*Central Clinical Hospital #1 of Russian Railways, Russia;*^*5*^*National Medical Center of Obstetrics, Gynecology and Perinatology, Russia*

Obesity, remaining a topical issue of modern medicine, leads to development of latent inflammation and insulin resistance in adipose tissue with subsequent development of type 2 diabetes mellitus (T2DM). Among patients with obesity, patients with obesity and normal glucose tolerance (NGT) are often encountered. In our study we addressed the question, if the adipose tissue derived mesenchymal stromal cells (ADSC) of these patients differ in their features and properties.

We isolated ADSC from samples of subcutaneous (sADSC) and visceral (vADSC) adipose tissue, which were obtained during bariatric surgery from patients with morbid obesity (BMI>35) and presence/absence DM2T (5/5 patients). ADSCs were analysed for autophagy by immunoblotting (measurement of expression LC3 and p62 for autophagy and phosphoJNK for inflammation). To evaluate the inflammatory state of adipose tissue, we examined level of M1 and M2 macrophages in cryosections of subcutaneous and visceral adipose tissue samples taken from patients.

We have shown that sADSC and vADSC from T2DM patients had significantly higher level of inflammation. Moreover, ADSC maintain inflammation at later passage such as 6^th^. According to measurement of p62 expression and LC3 modification we can say that ADSC from T2DM patients have less autophagy level compare with ADSC from NGT patients. Immunohistochemistry have shown that patients with T2DM have much more infiltration of CD68^+^-cells in subcutaneous and visceral adipose tissue and immunophenotype (balance of M1-M2 macrophages) of adipose tissue at this patients shift toward inflammatory state.

We assume that the findings will help to understand the causes of diabetes in obese patients. At this stage, it can be assumed that a decreasing of autophagy level and high inflammation associate with the development of T2DM.

Disclosure: This study was supported by Russian Scientific Foundation grant #17-15-01435.

## ECDO 16 Cell cycle arrest in mitosis or G2 promotes IFN-induced necroptosis

### Tanja Frank^1^, Sjoerd J.L. van Wijk^1^ and Simone Fulda^1,2,3^

#### ^*1*^*Goethe-University, Frankfurt, Germany;*^*2*^*German Cancer Consortium (DKTK), Heidelberg, Germany;*^*3*^*German Cancer Research Center (DKFZ), Heidelberg, Germany*

Resistance to apoptosis is a hallmark of cancer and deregulation of apoptosis often leads to chemoresistance. Therefore, new approaches to target cancer cells, which are unable to undergo apoptosis, are crucial for development of targeted cancer therapies. In the present study, we investigated the effect of cell cycle regulators on interferon (IFN)-induced necroptosis as an alternative cell death mechanism to overcome apoptosis-resistance.

HT29 cells, Capan-2 cells and MEFs were co-treated with several cell cycle regulators and IFNs. Cell death was measured by propidium iodide uptake to assess plasma membrane permeability. Cell cycle analysis was carried out by flow cytometry and the fraction of cells in mitosis was determined by immunofluorescent staining or protein expression analysis for the mitotic marker pH3. RNA interference was used for genetic silencing of necroptotic target genes. To check protein expression and post-translational modification of apoptotic or necroptotic markers, Western blot analysis was performed. For quantification of relative mRNA levels, real-time qPCR was carried out.

Here, we report a novel combination treatment of IFNs together with cell cycle arrest-inducing compounds that overcomes apoptosis resistance and elucidate the underlying molecular mechanisms. We find that combination treatment of IFNs (i.e. IFNβ) with inhibitors of the cell cycle (e.g. vinorelbine (VNR), aurora kinase A (AurA) inhibitor MLN 8237, nocodazole (Noc), polo-like kinase (Plk)-1 inhibitor BI 6727) co-operate to induce necroptotic cell death when caspase activation is blocked. The mode of cell death was confirmed by pharmacological inhibition as well as siRNA-mediated knockdown or knockout of the key necroptotic mediators receptor-interacting protein kinase (RIP)3 and mixed-lineage kinase-like (MLKL) in various cancer cell lines. Mechanistically, we show that necroptosis upon VNR/IFNβ/zVAD.fmk treatment is RIP1-independent but relies on IFNβ-induced gene expression of Z-DNA binding protein (ZBP)1 as shown by quantitative RT-qPCR and genetic knockdown experiments. Interestingly, we find that RIP3 is phosphorylated in response to compounds that trigger G2 or M arrest, even in the absence of IFNβ signaling and necroptosis induction.

This identification of a novel combination treatment that triggers necroptosis has implications for developing molecular targeted therapies to circumvent apoptosis resistance and point to an underestimated role of cell cycle regulation in cell death signaling.

## ECDO 17 The deubiquitinating enzyme USP22 modulates the ubiquitination of two key mediators of necroptosis, RIPK3 and MLKL

### Jens Rödig^1^, Sjoerd van Wijk^1^ and Simone Fulda^1,2,3^

#### ^*1*^*Goethe-University, Frankfurt, Germany;*^*2*^*German Cancer Consortium (DKTK), Heidelberg, Germany;*^*3*^*German Cancer Research Center (DKFZ), Heidelberg, Germany*

The Ubiquitin-specific peptidase 22 (USP22) is a member of the deubiquitinases (DUBs) family with diverse roles in regulating biological processes such as tumor development, cell growth and differentiation, gene expression and cell death. So far, USP22 and USP22 substrates are implicated in the regulation of SAGA-mediated gene transcription, apoptosis and autophagy. For example, silencing of USP22 in various cell lines resulted in an increase of apoptosis and autophagy due to the USP22/SIRT1 regulatory pathway. To further understand the role of USP22 in regulating different modes of cell death, we show here a novel role of USP22 in modulating necroptosis in colon carcinoma cells. Genetic knock-out of USP22 using CRISPR/Cas9 in HT-29 cells, revealed a significantly delayed resistance to death receptor-induced necroptosis. Utilizing proteome-wide ubiquitinome profiling using SILAC and LC-MS/MS, revealed USP22- and necroptosis-dependent alterations in two key players of necroptosis, RIPK3 and MLKL. Functional analysis revealed an increase in ubiquitination in the absence of USP22. Using TUBE (Tandem ubiquitin binding entities) pulldowns and in vivo deubiquinating assays, we verified increased RIPK3 and MLKL ubiquitination in the USP22 knockout cells. Our results show for the first time a DUB mediated MLKL deubiquitination in conjunction with necroptosis. Thus, we could identify USP22 as a crucial regulator of necroptosis by modulating the ubiquitination of RIPK3 and MLKL, which further elucidates the important role of deubiquitinating enzymes in regulating programmed cell death pathways.

## ECDO 18 Phosphatidylserine-dependent efferocytosis and entosis

### ^1^Shigekazu Nagata

#### ^*1*^*Osaka University, Osaka 565-0871, Japan*

Phosphatidylserine (PtdSer) is confined to the inner leaflet by the action of flippases that translocate PtdSer from the outer to inner leaflet. When cells undergo apoptosis, PtdSer is exposed to the cell surface, and is recognized as an “eat me” signal by macrophages for “efferocytosis”. Two P4-type ATPases (ATP11A and 11C) function as the flippase, and are inactivated by the caspase cleavage during apoptosis. This flippase-inactivation is necessary but not sufficient for the apoptotic PtdSer exposure. To quickly expose PtdSer to the surface, scramblases of the XKR family containing 10 transmembrane regions must be activated by caspase to non-specifically and bi-directionally translocate phospholipids between the inner and outer leaflets. Among the 9 Xkr members, Xkr8 is ubiquitously expressed, and the apoptotic PtdSer exposure and efferocytosis was strongly delayed with *Xkr8*^*-/-*^ hematopoietic cells. The two plasma membrane flippases (ATP11A and 11C) were ubiquitously expressed except for B cell progenitors, in which only ATP11C was present. *ATP11C*^*-/-*^ mice suffered from B cell lymphopenia, which could be rescued by double deficiency of *Axl* and *MerTK* genes coding for TAM tyrosine-kinase receptors involving PtdSer-dependent efferocytosis. The *ATP11C*^*-/-*^ B cell progenitors in *Axl*^-/-^*MerTK*^-/-^ background constitutively exposed PtdSer, and were efficiently engulfed by resident macrophages. These results indicate that PtdSer plays an important role not only in efferocytosis of apoptotic cells but also in entosis of living cells.

## ECDO 19 Inhibitors with high affinity to BCL-X_L_ protein cause protein kinase A activation in caspase-3-dependent manner in platelets

### Valentina S. Shpakova^1^, Stepan P. Gambaryan^1^, Natalia I. Rukoyatkina^1^

#### ^*1*^*Sechenov Institute of Evolutionary Physiology and Biochemistry of the Russian Academy of Sciences, S. Petersburg, Russia*

Thrombocytopenia and impaired activation of platelets are common side effects of anticancer therapy. The mechanisms of some low-molecular inhibitors of the pro-survival BCL-2 family proteins to block platelets activation are unknown. In our study, we used four inhibitors, WEHI-539, ABT-737, obatoclax and gossypol, and investigated whether they can affect platelets functions and the molecular mechanism(s) of inhibition of platelets activation. The compounds have different affinity to BCL-X_L_ protein which plays a crucial role in platelets viability. Only WEHI-539 and ABT-737 possess high affinity to BCL-X_L_.

Washed platelets from healthy donors were incubated with the compounds for 10 and 60 minutes and then prepared for FACS and Western blot analysis. Phosphatidylserine (PS) surface expression (Annexin V-PE) and αIIb*β*3 integrins activation (PAC-1-FITC) were assessed by flow cytometry. Vasodilator-stimulated phosphoprotein (VASP) phosphorylation and caspase-3 activation were analyzed by Western blotting.

PS exposure and caspase-3 activation were used as apoptosis markers. We found that obatoclax and gossypol induced PS exposure in 10 minutes but did not activate caspase-3. Nevertheless both obatoclax and gossypol impaired platelet αIIb*β*3 integrins activation induced by TRAP-6, the agonist of thrombin PAR-1 receptors. ABT-737 and WEHI-539 caused caspase-dependent apoptosis in platelets in 1 hour and blocked their activation. The major inhibitory processes in platelets are mediated by the activation of adenylate (AC) and guanylate cyclases (GC) and synthesis of cyclic AMP (cAMP) and cyclic GMP (cGMP) which activate protein kinases A (PKA) and G (PKG) respectively. Activation of the inhibitory pathways can be monitored by the phosphorylation of the substrate protein VASP. In our study both ABT-737 and WEHI-539, but not obatoclax and gossypol caused VASP phosphorylation, which indicated activation of inhibitory PKA/PKG pathways. Neither ABT-737 nor WEHI-539 inhibited PP1 and PP2 phosphatases activity which also could lead to VASP phosphorylation. ABT-737 and WEHI-539 did not elevate cAMP or cGMP level in platelets and, at the same time, AC and GC inhibition did not prevent VASP phosphorylation caused by the inhibitors. However, PKA or caspase-3 inhibition prevented VASP phosphorylation induced by ABT-737 or WEHI-539, which indicates that these compounds activate PKA in AC-independent but caspase-3-dependent manner.

In summary, we showed that only high-affinity BCL-X_L_ inhibitors cause apoptosis in platelets and block their functions through PKA activation in caspase-3-dependent pathway. These data indicate a new AC-independent way of PKA activation in apoptotic cell.

Disclosure: The reported study was funded by RFBR according to the research project №17-00-00141 (17-00-00139).

## ECDO 20 TRAF1 does not compensate for TRAF2 deficiency in tumor necrosis factor (TNF) receptor superfamily (TNFRSF) signaling

### Jennifer Kreckel^1^, Daniela Siegmund^1^, Harald Wajant^1^

#### ^*1*^*University Hospital Wuerzburg, Germany*

TNF receptor associated factor1 (TRAF1) and TRAF2 are part of the ligand-induced signaling complexes utilized by receptors of the TNF receptor superfamily (TNFRSF). TRAF2 assembles into trimeric molecules with the ability to bind a single molecule of either cIAP1 and cIAP2. TRAF1 forms heterotrimers with TRAF2 and interacts with cIAP1 and cIAP2 in a similar fashion as TRAF2. It is, however, poorly understood whether, and if yes to which extent, TRAF1 can fulfill similar functions as TRAF2.

The aim of the study was to investigate the role of TRAF1 in signaling by receptors of the TNFRSF and its relation to TRAF2.

We generated TRAF1 expressing HCT-116 cells but also TRAF2 deficient HCT-116 cells and a counterpart reconstituted with TRAF1. We investigated in the various HCT-116 variants the ability of the TNFRSF receptors TNFR1, Fn14 and LTbetaR to activate caspase-8 and proinflammatory pathways.

Ectopic TRAF1 expression showed nearly no effect on signaling by the various TNFRSF receptors. TRAF2 knockout led to reduced classical NF-kappaB signaling and enhanced TNF-induced caspase-8 cleavage at lower ligand concentrations. Additionally, the alternative NF-kappaB pathway was constitutively active. Surprisingly, we did not observe any functional reconstitution of TNFRSF receptor signaling in TRAF1 transfected TRAF2 deficient cells, whereas TRAF2 reconstitution restored functionality.

Our data suggest that TRAF1, in contrast to TRAF1-TRAF2 heterotrimers does not substitute for TRAF2 in TNFRSF receptor signaling. It remains our aim to investigate the role of TRAF1 further.

## ECDO 21 The role of PERK-E-Syt1 interaction in mitochondria homeostasis and lipid trafficking at the ER-mitochondria contact sites

### Maria Livia Sassano^1^, Alex R. Van Vliet^1^, Sofie Van Eygen^1^, Rita Derua^1^, Jonas Dehairs^1^, Johan Swinnen^1^, Paolo Pinton^2^, Patrizia Agostinis^1^

#### ^*1*^*Laboratory of Cell Death Research and Therapy, Department for Cellular and Molecular Medicine, University of Leuven (KU Leuven), Campus Gasthuisberg, O&N1, Herestraat 49, Box 802, 3000 Leuven, Belgium;*^*2*^*University of Ferrara, Via Fossato di Mortara 70 (c/o CUBO),44121 Ferrara, Italy*

Recent evidence show that the endoplasmic reticulum (ER) pancreatic kinase PERK, a key mediator of the unfolded protein response (UPR), is also involved in signaling processes beyond the UPR. Interestingly, works from our laboratory indicated that PERK is an important coordinator for the formation of the ER-organelles contact sites, such as the ER-plasma membrane (PM) juxtapositions and the mitochondria-associated membranes (MAMs). Our studies show that PERK is enriched at the ER-mitochondria contacts and its deletion decreases the proximity between these two compartments under homeostatic conditions. In line with this, we found that PERK deficiency counteracts the mitochondrial calcium uptake, following depletion of the ER Ca^2+^ stores by IP_3_ mobilizing agents. Analysis of the PERK-interactome and co-IP assays uncovered that PERK physically interacts with the Ca^2+^-regulated phospholipid binding protein Extended Synaptotagmin-1 (E-Syt1), which tethers the ER to the PM upon ER store Ca^2+^ depletion.

Here we reveal that PERK and E-Syt1 interact within the MAMs subdomain and we mapped the specific domains of E-Syt1 responsible for this interaction. Furthermore, we found that PERK is required for the recruitment of E-Syt1 at MAMs.

Lipidomics analysis of MAM- and mitochondria fractions show that the presence of PERK, as well as E-Syt1, modulate the distribution of phospholipids, such as phosphatidylserine (PS) and phosphatidylinositol (PI), within these compartments, thereby suggesting that PERK-E-Syt1 interaction at MAMs regulates the lipid trafficking between ER and mitochondria.

Ongoing studies are assessing whether the modulation of PERK-E-Syt1 interaction has an effect on the mitochondrial respiration and morphology.

## ECDO 22 p53-independent NOXA induction by fluorizoline is regulated by ATF3 and ATF4 in HeLa cells

### Sonia Núñez-Vázquez^1^, José Saura-Esteller^1^, Ismael Sánchez-Vera^1^, Ana M. Cosialls^1^, Gabriel Pons^1^, Daniel Iglesias-Serret^1^, Joan Gil^1^

#### ^*1*^*Universitat de Barcelona-Institut d'Investigació Biomèdica de Bellvitge (IDIBELL), L'Hospitalet de Llobregat, Catalunya, Spain*

Fluorizoline, a diaryl trifluorothiazoline compound, induces p53-independent apoptosis in a wide range of tumor cell lines. Although its mechanism of action is not entirely understood yet, we know that Prohibitin (PHB) 1 and 2 are targets of fluorizoline and it has been established that fluorizoline induces the mitochondrial apoptotic pathway predominantly through the upregulation of NOXA.

Latest results show that PHB interference does not confer resistance to fluorizoline-induced apoptosis in HeLa cells, in contrast to other cell lines. On the other hand, we observe that exposure to fluorizoline triggers a endoplasmic reticulum (ER) stress response, as shown by the increase of CHOP levels, eIF2α phosphorylation and Xbp-I splicing.

In parallel, we have found that the transcriptional factors ATF3 and ATF4 are induced by fluorizoline. Moreover, a NOXA promoter construction with the CRE binding site mutated displays a reduction in its luciferase activity response to fluorizoline while a promoter without p53 does not show any changes. Furthermore, we demonstrate that ATF3 and ATF4 bind to NOXA promoter upon fluorizoline treatment and ATF4 presence is critical to induce apoptosis and NOXA upregulation by fluorizoline.

In conclusion, we show that NOXA upregulation is critical for fluorizoline-induced apoptosis and this induction is regulated at the transcriptional level by ATF3 and ATF4, caused probably by an increase of ER stress.

## ECDO 23 Recombinant analogon RL2 of human κ-Casein induces cell death in breast cancer cell lines

### Max Richter^1^, Fabian Wohlfromm^1^, Olga Koval^2^, Thilo Kähne^1^, Vladimir Richter^2^, Inna Lavrik^1^

#### ^*1*^*Otto von Guericke University, Magdeburg, Germany;*^*2*^*Institute of Biological Chemistry and Fundamental Medicine, Novosibirsk, Russia*

Breast carcinoma is with a frequency of 30% the most occurring cancer for women. Specified therapeutics are indispensable for optimal treatment. In this study we demonstrate that RL2, the recombinant fragment of human κ-Casein, induces cell death in breast cancer cell lines in caspase-independent and dependent manners. Various non-breast cancer cell lines are unaffected by RL2 treatment. In breast carcinoma MDA-MB 231 and MCF-7 cells previous studies showed the impact of RL2 on caspase-3 activation. Here we demonstrate that the induction of cell death by RL2 is only partially caspase-3/7 dependent. The mass spectrometry-based identification of RL2 interaction partners identified various potential targets for cell death mediation. The interaction with mitochondrial import protein TOM70 provides new insights into the connection of RL2 and mitochondrial induced cell death. Downregulation of TOM70 reduces RL2-mediated cell death effects. Other RL2-interacting proteins as Cathepsin D and cell surface receptors that were identified by proteomics screening could contribute to the strong cell death-inducing effect and contribute to the breast cancer specifity of RL2. So far it remains unclear how RL2 gains this specifity and if healthy breast tissue is affected by RL2 treatment.

## ECDO 24 Regulation and stabilization of death-inducing signaling complex (disc) by its key proteins

### L. K. Hillert^1^, N. V. Ivanisenko^2^, J. Espe^1^, C. König^1^, D. Busse^1^, I. N. Lavrik^1^

#### ^*1*^*University Magdeburg, Madgeburg Germany;*^*2*^*Institute of Cytology and Genetics SB RAS, Novosibirsk, Russia*

The formation of the death-inducing signaling complex (DISC) is the first crucial step to activate extrinsic apoptosis. CD95 DISC comprising CD95, FADD, c-FLIPs, procaspase-8 and procaspase-10 is essential for the assembly of DED filaments that serve as a platform for procaspase-8 activation. This activation is crucially controlled by c-FLIP proteins: c-FLIP_Short_ and c-FLIP_Long_. In this study we further unraveled the mechanisms of c-FLIP action at the DED filaments. To address this issue, we have generated HeLa cells stably overexpressing different c-FLIP isoforms. We discovered that an increase of c-FLIP isoforms c-FLIP_Short_ and c-FLIP_Long_ directly influences the caspase-8 filament formation. In particular, quantitative mass spectrometry analysis with AQUA peptides supported by biochemical analysis and imaging flow cytometry demonstrated that the overexpression of c-FLIP proteins stabilizes the DED filaments and changes their stoichiometry. In addition, to distinguish the different stages of the apoptotic pathway and link them to different stages of the DISC assembly we used the cutting edge technology of imaging flow cytometry analysis (AMNIS^®^) which combines cell imaging and flow cytometry in one measurement circuit. Finally, computational modelling allowed us to get more information about the dynamics of the DISC and its stability which offers new points of application in the discovery of novel medical drugs. Our latest findings will be presented.

## ECDO 25 Screening for novel E3 ubiquitin ligases to regulate Necroptosis

### Yu-Jin Ha^1^, Hyun-Jin Noh^1^, Seung-Jae Oh^1^, Iseul Yoo^1^, Han-Hee Park^1^, Se-Yeon Park^1^, You-Sun Kim^1^

#### ^*1*^*Ajou University, Suwon, Korea*

Necroptosis is a form of regulated cell death and distinguished from apoptosis morphologically and biochemically. Necroptosis plays substantial roles in pathological processes, including a facilitative role in tissue damage, such as in ischemia-reperfusion injury, and also in host defense of viral infections. The recent fascination with necroptosis is largely the result of an increased realization that it plays many physiological and pathological roles. Following Tumor Necrosis Factor (TNF) Receptor 1 (TNFR1) ligation, the protein kinase, RIP1 (Receptor Interacting Kinase 1), activates RIP3 presumably by promoting RIP3 autophosphorylation, which in turn leads to MLKL (Mixed Lineage Kinase domain-Like) activation. Diverse necroptotic stimuli lead to the activation of cell death through the modulation of RIP1 and/or RIP3 kinase activity. However, recent studies imply a more diverse picture of post-transcriptional modifications regulating the necrosome. The importance of ubiquitylation in the regulation of TNF-induced necroptosis pathway has been emphasized and ubiquitin-mediated protein turnover is involved in regulating the activity and/or stability of a number of TNF-induced necroptosis pathway components. Aside from phosphorylation, ubiquitination of necrosome components is probably the most important post-translational modification and different ubiquitin patterns on these are known to dictate the fate of cells. To identify novel E3 ubiquitin ligases which regulate necroptosis by ubiquitylation, we searched potent E3 by extensive cross-screening of our E3 libraries which include over 200 of E3. Among them, we selected several E3 ubiquitin ligases which could block the TNF-induced Necroptosis. These novel E3 will be studied to understand the molecular mechanisms that how they target the necrosome components and control cell death. A better understanding of the regulation of post-translational modification of necrosome may thus help the development of drugs for various necroptosis-related diseases.

## ECDO 26 HDAC6 may influence the death of brain cells in the early post-stroke recovery period

### Demyanenko S.^1^, Berezhnaya E.^1^,Neginskaya M.^1^, Nikul V.^1^

#### ^*1*^*Academy of Biology and Biotechnology, Southern Federal University, Rostov-on-Don, Russia*

We studied the expression of HDAC6 at 3, 7, 14, and 21 days after photothrombotic stroke (PTS) in the mouse somatosensory cortex using immunohistofluorescence and western-blot analysis. The unilateral irradiation of the somatosensory cortex was performed through the cranial bone using a 532 nm diode laser (0.2 W/cm^2^, Ø 1 mm, 15 min). We studied the level of HDAC6 in intact regions of the ipsilateral cortex, contralateral hemisphere, and hippocampus.

Histone deacetylase 6 (HDAC6) belongs to the IIb class histone deacetylases. HDAC6 is a predominantly cytoplasmic enzyme. However, in our studies, HDAC6 was present both in the cytoplasm and in the nuclei of most neurons, but not astrocytes in the cortex and hippocampus.

In the early recovery period after the stroke (3, 7, and 14 days), a growth in HDAC6 in cortical and hippocampal neurons was observed near the injury and, remarkably, in the contralateral hemisphere. The quantitative determination of apoptosis in HDAC6-positive cells in our study indicates its growth in the cortical cells of the damaged hemisphere during the period of growth in the protein expression. This may be connected with the observed growth in the nuclear import of HDAC6.

Thus, the results that we obtained show that inhibition of HDAC6 may protect neurons and glia from ischemic damage and help restore the brain tissue after stroke.

The study was supported by the Russian Foundation for Basic Research (project no. 16-04-01135).

## ECDO 27 A comparative study of apoptosis in peripheral blood leukocytes in patients with Parkinson's disease and healthy donors

### Julia Teterina^1^, Anna Boyko^1^, Olga Shustova^1^, Maria Grechikhina^1^, Ekaterina Doronina^1^, Natalia Troyanova^1^, Elena Kovalenko^1^, Alexander Sapozhnikov^1^

#### ^*1*^*Shemyakin – Ovchinnikov Institute of Bioorganic Chemistry, Moscow, Russia*

Parkinson's disease (PD) is one of the most common neurodegenerative diseases. In recent years, there are growing evidences that the pathogenesis of this disease is connected with regional and peripheral immune processes. Currently, the association of clinical signs of PD with different characteristics of patient immune status is actively being searched. In the framework of this problem we perform an investigation of functional state of immune cells, in particular, level of apoptosis and autophagy (p62 expression) in cultivated in vitro fractions of peripheral blood leukocytes isolated from a group of patients with Parkinson's disease in comparison with a group of healthy donors.

29 healthy donors and 27 patients with PD participated in the study. Flow cytometric analysis of early stage (Anexin V+/PI-) of spontaneous and induced (by oxidative stress) apoptosis in the samples of peripheral blood mononuclear cells (PBMC) revealed that proportion of apoptotic cells in the PD group was increased in comparison with the values in the healthy group. This was detected for both, the model of spontaneous apoptosis (54.1% and 29.8%, respectively, p=0.005), and the model of induced (50 µM H_2_O_2_, 20 hours) apoptosis (55.2% and 38%, respectively, p=0.01). No significant difference in these models was registered for the samples of peripheral blood granulocytes (PMN). Evaluation of the activity of the process of autophagy in leukocytes was analyzed by detection of intracellular content of the important marker of autophagy – protein p62. Analysis of the data measured by ELISA in the cell samples lysates showed that intracellular p62 level in PBMC was significantly higher in the PD group compared to the healthy group (3.25 ng/ml and 1.14 ng/ml, respectively, p=0.007). No significant difference in this model was registered for the samples of PMN.

The registered differences between patients and healthy donors can be considered as the possible peripheral biomarkers of PD pathogenesis; application of these biomarkers for clinical practice might extend the current approaches to diagnostics and analysis of the course of the PD.

Disclosure: This work was supported by Russian Science Foundation, grant No. 16-15-10404.

## ECDO 28 Deregulation of mTORC1/autophagy axis during senescence leads to death of Ras-transformed cells upon MEK/ERK inhibition

### Kochetkova E.Y.^1^, Blinova G.I.^1^, Bystrova O.A.^1^, Martynova M.G.^1^, Pospelov V.A.^1^, Pospelova T.V.^1^

#### ^*1*^*Russian Academy of Science, Moscow, Russia*

Ras GTPase is mutated in about 30% of tumors, and these tumors are among the most aggressive and therapy-resistant, as mutant Ras provides tolerance to therapy. Thus, understanding the mechanisms of Ras-transformed cells’ resistance to tumor suppression agents is of fundamental importance to counter cancers characterized with oncogenic overexpression of Ras. One of novel strategies against Ras-transformed cells is based on the use of kinase inhibitors of the Raf/MEK/ERK pathway. However, Ras-expressing cells overcome the antiproliferative and pro-apoptotic effects of these inhibitors through activation of cytoprotective autophagy. MEK/ERK pathway suppression with PD0325901 in *E1A+cHa-Ras* transformed rat embryo fibroblasts (ERas cells) results in complete inhibition of ERK1,2 phosphorylation and mitochondria damage. However, cells successfully overcome the effect of PD0325901 by activating AMPK-regulated autophagy and restore their viability. Therefore, autophagy is a desirable target in development of strategy for elimination of Ras-expressing cells, but direct suppression of autophagy with inhibitors like chloroquine didn’t lead to effective cell death. In our research we aimed to find a strategy to deprive cytoprotective properties of autophagy in Ras-expressing cells upon treatment with MEK1,2 kinase inhibitor PD0325901. We used histone deacetylase inhibitor sodium butyrate (NaBut) to induce senescence. Senescence is linked with high constitutive activity of mTORC1 that stimulates protein, lipid and nucleotide synthesis and inhibits cellular catabolic processes, first of all, autophagy. The recruitment of the mTORC1 to the lysosomal membranes has been shown to be essential for mTOR Complex 1 activation and attenuation of autophagy to sustain senescent hypertrophic hypersecretory phenotype. Upon MEK/ERK suppression, senescent ERas cells fail to eliminate the damaged mitochondria and fail to rescue viability. In ERas cells treated with MEK and HDAC inhibitors the autophagosome-lysosome fusion does not take place that leads to accumulation of damaged mitochondria and reactive oxygen species. The incapability of senescent cells to terminate cytoprotective autophagy is due to localization of lysosomes, carrying constitutively active mTORC1 and AMPK, in perinuclear region, while autophagosomes are formed predominantly at the periphery of the cytoplasm. In senescent cells, lysosome-bound mTORC1 is active even in conditions of growth factor and amino acid deprivation, thus attenuating autophagy. As a result, upon MEK/ERK suppression autophagosomes don’t fuse with lysosomes, don’t degrade damaged mitochondria, and cells undergo apoptosis. We found that senescent cells acquire unique sensitivity to MEK/ERK kinase inhibitor due to the spatial separation of the participants involved in autophagy process. Consequently, the use of MEK/ERK kinase inhibitors together with senescence inducer represents a promising strategy for elimination of Ras-expressing tumor cells.

Disclosure: This work was supported by Russian Scientific Foundation № 14-50-00068.

## ECDO 29 To stimulate or inhibit cAMP signal in regulating cardiac myocyte function and death

### Yoshihiro Ishikawa^1^

#### ^*1*^*Yokohama City University School of Medicine, Yokohama, Japan*

Activation of the sympathetic nerves initiates the most potent stimulus for changing the metabolic status and enhancing cardiac output, both acutely and chronically. Norepinephrine released from the sympathetic nerve terminals binds to beta-adrenergic receptors and activates the stimulatory G protein, Gs. Gs then stimulates adenylyl cyclase, a membrane-bound enzyme that catalyzes the conversion of ATP to cAMP. cAMP, an intracellular second messenger, activates protein kinase A and Epac. Protein kinase A initiates a series of enzymatic reactions leading to a phosphorylation cascade, activating multiple proteins that regulate both the rate and force of cardiac contraction. Epac initiates small G protein-related reactions. The integrated effect of these reactions is an enhancement in cardiac output by increasing both oxygen and metabolic demands. Thus, catecholamine is needed to stimulate cAMP signal to increase its pump function in acute heart failure. Since the identification of cardiac adenylyl cyclase, i.e., the type 5 subtype, in 1991, we have investigated the role of cAMP signal in promoting myocyte cell death. The type 5 subtype exhibits an extremely high enzymatic activity. Upon activation, a large amount of cAMP is produced with increased reactive oxygen species, leading to myocyte death. Mice, in which Gs is overexpressed, cardiac function is increased at young age while decreased at old age with development of cardiac myocyte apoptosis. Mice, in which type 5 adenylyl cyclase is knocked out (AC5KO), are resistant to various cardiac stresses, including pressure overload or chronic catecholamine infusion, and have increased median lifespan. Old AC5 KO mice are protected from aging-induced apoptosis and fibrosis with activated Raf/MEK/ERK signaling pathway and superoxide dismutase. Knocking out of Epac1 showed similar resistance to cardiac stresses, including aging or catecholamine stress. Cardiac toxicity evoked by type 5 adenylyl cyclase can be prevented by Epac1 knockout. Thus, it is necessary to stimulate cAMP signal to enhance cardiac function, however, over activation of cAMP signal may induce cardiac myocyte apoptosis. Targeting a specific molecule(s) to inhibit cAMP signal, may be useful to prevent myocyte apoptosis without decreasing cardiac function.

## ECDO 30 Gambogic acid triggers paraptosis in cancer cells via disruption of thiol proteostasis

### Min Ji Seo^1^, Dong Min Lee^1^, In Young Kim^1^, Dong Joo Lee^1^, Min-Koo Choi^2^, Joo-Youn Lee^3^, Seok Soon Park^4^, Seong-Yun Jeong^4^, Eun Kyung Choi^4^, Kyeong Sook Choi^14^

#### ^*1*^*Ajou University, Suwon, Korea;*^*2*^*College of Pharmacy, Dankook University, Cheoan, Korea;*^*3*^*Chemical Data-Driven Research Center, Korea Research Institute of Chemical Technology, 141 Gajeong-ro, Yuseong-gu, Daejeon, Korea;*^*4*^*University of Ulsan College of Medicine, Seoul 05505, Korea*

The anticancer activity of GA, a xanthonoid extracted from the resin from Garcinia hanburyi, was recently reported in many studies, but its underlying mechanism still needs to be clarified. We show here that GA induces the cancer cell death accompanied by vacuolation not only in vitro but also in vivo. GA-induced vacuolation in various cancer cells was derived from the dilation of the endoplasmic reticulum (ER) and mitochondria, which was blocked by cycloheximide, suggesting that GA kills cancer cells via induction of paraptosis. We found that megamitochondria formation due to the fusion of swollen mitochondria preceded the fusion of ER-derived vacuoles. While GA-induced proteasomal inhibition contributes to the ER dilation and ER stress, mitochondrial membrane depolarization was followed by megamitochondria formation. Interestingly, GA-induced paraptosis was effectively blocked by various thiol-containing antioxidants, independently of ROS generation. We observed that GA can react with cysteinyl thiol to form Michael adducts, suggesting that the ability of GA to covalently modify nucleophilic cysteinyl group of the proteins may cause protein misfolding and accumulation of misfolded proteins within the ER and mitochondria. Collectively, our findings that disruption of thiol proteostasis may critically contribute to the anti-cancer effects of GA via induction of paraptosis.

Acknowledgment: MJS and DML contributed equally.

## ECDO 31 ER stress, aldo-keto reductases and cancer cell resistance to ferroptotic cell death

### Gagliardi Mara^1,2^, Mauro Piacentini^1^, Corazzari Marco^2^

#### ^*1*^*University of Rome Tor Vergata, Rome, Italy;*^*2*^*University of Piemonte Orientale, Novara, Italy*

Currently, cancer remains the challenge of the most difficult to face and fight for both clinicians and researchers. This is mainly due to the high heterogeneity of its nature and the ability to develop a complicated and extremely efficient repertoire of defense mechanisms to counteract therapeutic strategies. Therefore, the most effective therapies tend to be those developed on the individual tumor types. Nevertheless, even in these cases there is a high internal variability due to the intrinsic high heterogeneity of each type of tumor. The result is a continuous and increasing effort in developing alternative therapeutic strategies to counteract tumor development and resistance. Recently, an alternative non-apoptotic cell death pathway has been discovered, named ferroptosis. This process is stimulated by the inhibition of the XC^-^ system resulting in glutathione depletion, ER stress induction and generation of lipid-ROS, the key executioners of the process. This is a promising novel anti-cancer strategy since it seems to be independent from the classical mediators of apoptosis, frequently mutated in most cancers. However, as evidence of the remarkable tumor efficiency of developing countermeasures to ensure their own survival, even in this case some tumors show resistance to this new cell death process. In fact, we found human skin melanoma cells are able to express/activate downstream specific enzymes, aldo-keto reductases (AKRs), to detoxify ALOX-generated lipid-ROS, thus inhibiting the execution of the ferroptotic process, sustain cell survival. However, abrogating AKRs activity/expression resensitize melanoma cells to ferroptosis, thus identifying the combined treatment of pro-ferroptotic drugs plus AKRs inhibitors as a new valuable therapeutic strategy to treat human skin melanoma. Moreover, we also found the ferroptotic process is ER stress independent in melanoma cells, in which a pivotal role is played by the oxidative stress related transcription factor Nrf2.

## ECDO 32 Mechanisms of resistance to targeted therapies in melanoma and myelodysplastic syndromes: characterization and validation of innovative covalent inhibitor

### Marwa Zerhouni^1^, Anthony Martin^1^, Rachid Benhida^1^, Stéphane Rocchi^1^, Guillaume Robert^1^, Patrick Auberger^1^

#### ^*1*^*Université Côte d’Azur, C3M/Inserm U1065, Nice, France*

High risk Myelodysplastic Syndromes (MDS) and Metastatic Melanoma (MM) are highly aggressive and heterogeneous cancers with high rates of resistance to their targeted chemotherapeutic treatment. Despite of approximately 50% initial response to Vidaza (5-azacytydine) for MDS and 80% to BRAF inhibitors for BRAF mutated MM patients, majority of patients will eventually experience therapeutic failure by becoming resistant. This acquired resistance can be explained by epigenetic modifications such as aberrant DNA methylations or point mutations, which in turn can be used as prognostic markers for cancers’ initiation, progression and aggressiveness. As such, developing innovative compounds against MDS and MM seems to be a key point to offer second line treatment to patients in therapeutic failure. While covalent inhibitors have been considered as too toxic on healthy cells in vitro and not enough selective compared to targeted therapies, the interest for this class of compounds is growing since the FDA approval of ibrutinib in 2013 for mantle lymphoma and other pathologies in 2014 and 2017.

Here, we present the characterization and preclinical validation of a novel covalent inhibitor HA 344 non-nucleoside derivative of Acadesine, efficient on both sensitive and resistant MDS and MM while relatively not toxic on healthy PBMC and fibroblasts. Our recent studies have shown that HA 344 enters cells through ENT (nucleoside analog) transporters to induce a blockade of cell cycle followed by a caspase independent cell death. HA 344 activates DNA damage and stress signaling response involving H2AX, p38MAPK and JNK. Moreover, electron microscopy allowed to determine that HA 344 is an inhibitor of pro-survival functions of autophagy. As we characterized that HA 344 is a covalent inhibitor, we generated and used a clickable form of our HA 344 to trap the “*in cellulo”* molecular target(s). This powerful technique was coupled to mass spectrometry to deeply identify a covalent target: PKM2 involved in glucose metabolism. The biological effect of targeting PKM2 on MDS and MM cells must be determined and fully characterized. Finally, an in vivo study on sensitive and resistant melanoma xenografts showed no toxicity of HA 344 in mice and a significant decrease in tumor volume.

This study allows to consider covalent inhibitors targeting metabolic pathways as potential innovative second line treatments against MDS and MM resistant to conventional chemotherapy.

## ECDO 33 Novel ARTS mimetic small molecules bind directly to XIAP and promote killing of cancer cells through degradation of XIAP and Bcl-2

### ^1^Sarit Larisch, ^1^Dana Mamriev, ^1^Juliana Kagan

#### ^*1*^*University of Haifa. Haifa, Israel*

Inhibitor of Apoptosis (IAP) proteins are expressed at high levels in many human cancers, thus representing favorable therapeutic targets. In cells undergoing apoptosis the inhibition of caspases by XIAP (X-linked Inhibitor of Apoptosis) has to be overcome to enable apoptosis. This is achieved by natural XIAP-antagonists. The most widely used strategy for targeting IAPs is based on mimicking the IAP-antagonist, SMAC/Diablo. These molecules, called SMAC mimetics, act primarily by neutralizing and degrading cIAPs. Here we report of a new class of specific XIAP antagonists which mimic the function of ARTS protein. ARTS (Sept4_i2) is a mitochondrial pro-apoptotic tumor suppressor protein. ARTS induces apoptosis by directly binding and degrading the two major proteins inhibiting apoptosis- XIAP and B-cell lymphoma 2 (Bcl-2) (Edison et al., Cell Reports, 2017 21(2), 442-454). ARTS is localized at the outer mitochondrial membrane and initiates caspase activation upstream of mitochondrial outer membrane permeabilization (MOMP). We have recently shown that ARTS binds directly to Bcl-2 and XIAP, bringing them into close proximity and allowing XIAP-the E3-ligase to degrade Bcl-2 via the Ubiquitin Proteasome System (UPS).

ARTS binding to XIAP is different from any other known IAP-anatgonists. ARTS contains a unique binding site to XIAP and binds to distinct sequences within BIR3/XIAP which are not bound by SMAC/Diablo.

We performed a computational “*in silico*” screen” of 3.5M compounds to identify small molecules that fit into the unique ARTS binding groove between ARTS and XIAP. The best fitting 100 molecules resulting from this screen were tested for their ability to kill A375 melanoma and Jurkat leukemia cells. Two small molecules showing potent killing of these cells were further tested on a 93 panel of cancer cell lines representing 18 different cancer types. While selective killing of specific cell types was demonstrated for each of these compounds, none of them killed normal PBMC (peripheral blood mononuclear cells). The X4 compound showed specific direct high affinity binding to XIAP, but not to cIAP in MST (Microscale Thremophoresis) assays. Furthermore, X4 induced UPS-mediated degradation of endogenous XIAP and Bcl-2 and promoted apoptosis. These compounds are unique in their ability to stimulate degradation of both XIAP and Bcl-2. As levels of these two anti-apoptotic targets are high in many types of cancers, ARTS-mimetics represent a novel class of dual antagonists that have considerable potential for cancer therapy.

## ECDO 34 Rational design of molecular probes targeting caspase-8 /c-FLIP_L_ heterodimer in the Death-Inducing Signaling Complex

### Nikita V. Ivanisenko^1^, Laura K. Hillert^2^, Denise Busse^2^, Johannes Espe^2^, Vladimir A. Ivanisenko^1^, Inna N. Lavrik^2^

#### ^*1*^*The Siberian Branch of the Russian Academy of Sciences, Prospekt Lavrentyeva 10, Novosibirsk, 630090, Russia;*^*2*^*University Magdeburg, Magdeburg, 39106, Germany*

The induction of extrinsic apoptosis via CD95 and TRAILR1/R2 is triggered by the activation of corresponding receptors, which leads to the formation of the Death-Inducing Signaling Complex (DISC). Caspase-8 activation at the DISC is largely controlled by cellular FLICE inhibitory proteins (c-FLIPs). Heterodimer formation of procaspase-8 and c-FLIP is considered to be one of the key events in the regulation of apoptosis activation. However detailed molecular mechanisms of this control are yet to be investigated. To further unravel this question we applied advanced *in silico* methods of virtual screening and mathematical modeling. Using virtual screening of large databases of chemical compounds we could identify first in class chemical probe targeting heterodimer caspase-8/c-FLIP_L_. Identified chemical compound termed FLIPinBγ was shown to enhance caspase-8 activity at the DISC and promote cell death. Furthermore, a kinetic mathematical model was further developed to analyze the observed effects of FLIPinBγ on DISC activation. Based on the modeling results we could predict that the FLIPinBγ/caspase-8/c-FLIP_L_ complex plays a significant role at the very initial stages of the DED chain assembly and procaspase-8 processing. Furthermore its activity can be enhanced up to several times upon increase of c-FLIP_L_/procaspase-8 ratio, suggesting that compounds targeting caspase-8/c-FLIP_L_ heterodimer have a high therapeutic potential in cancer cell lines that are characterized by high c-FLIP levels.

Disclosure: Supported by the Russian Science Foundation grant 14-44-00011.

## ECDO 35 The Prohibitin-binding compound fluorizoline increases ER stress and activates the intrinsical pathway of apoptosis in HEK293T and U2OS cells

### ^1^José Saura-Esteller, ^1^Sonia Núñez-Vázquez, ^1^Ismael Sánchez-Vera, ^1^Ana M Cosialls, ^1^Gabriel Pons, ^1^Daniel ^1^Iglesias-Serret, ^1^Joan Gil

#### ^*1*^*Universitat de Barcelona-Institut de Investigació Biomèdiqca de Bellvitge (IDIBELL), L’Hospitalet de Llobregat, Catalunya, Spain*

Fluorizoline was selected as the lead compound of a new family of compounds with fluorinated thiazoline scaffold due to its high pro-apoptotic activity. Fluorizoline treatment resulted in BAX-BAK dependent apoptosis in HEK293T and U2OS cell lines. Moreover downregulation of Prohibitins, the molecular target of fluorizoline, resulted in increased resistance to this compound as previously observed in MEF cells. Individual downregulation of NOXA, BIM, PUMA and simultaneous knockout of NOXA and BIM in HEK293T was not sufficient to render HEK293T cells resistant to fluorizoline-induced apoptosis. However, RNA interference of PUMA in NOXA-/-/BIM-/- HEK293T increased cell viability after fluorizoline treatment.

Treatment with this drug results in increased protein expression of the Integrated Stress Response proteins ATF4, ATF3, and CHOP and increased phosphorylation of eIF2a and JNK. In spite of this, individual RNA interference and chemical inhibition of these proteins had no effect on cell viability. Increased levels of spliced XBP1 were also detected following fluorizoline treatment, indicating activation of the ER-stress receptor IRE1β. Study of Ca+2 levels revealed a quick increase of intracellular Ca+2 concentration after fluorizoline treatment that could be responsible for the effect observed in ER stress related proteins.

In conclusion, we have further validated the requirement of Prohibitins for fluorizoline-induced apoptosis in HEK293T an U2OS and we have identified the BH3-only proteins responsible for the induction of the mitochondrial pathway of apoptosis in HEK293T cells. Finally we have demonstrated that fluorizoline treatment results in increased ER-stress and activation of the Integrated Stress Response that might be caused by alteration of Ca+2 homeostasis.

## ECDO 36 Fluorizoline induced-apoptotic mechanism in the near-haploid human cell line HAP1

### ^1^Ismael Sánchez-Vera, ^1^José Saura-Esteller, ^1^Sonia Núñez-Vázquez, ^1^Ana M. Cosialls, ^1^Daniel Iglesias-Serret, ^1^Gabriel Pons and Joan Gil

#### ^*1*^*Universitat de Barcelona-Institut d’Investigació Biomèdica de Bellvitge (IDIBELL), L’Hospitalet de Llobregat, Catalunya, Spain*

We previously described fluorizoline (diaryl trifluorothiazoline) as a new apoptosis inducing compound. This drug leads to apoptosis by selectively binding to prohibitin (PHB) and in a p53 independent manner. It has been described that fluorizoline induced apoptosis takes place through the intrinsic pathway by modulation of BCL-2 pro-apoptotic family members. In this context, we have assessed the mechanism triggered by our compound in HAP1 cell line. HAP1 is a near-haploid human cell line (HAP1), derived from a chronic myelogenous leukaemia cell line (KBM7), and due to its haploid features provides us a great tool for gene edition.

We have characterized that fluorizoline triggers apoptosis by the intrinsic pathway in HAP1 cells, as BAX/BAK interference prevents the apoptotic outcome. NOXA, a BCL-2 pro-apoptotic member, is upregulated upon fluorizoline treatment at both transcriptional and translational levels. Hence, HAP1 NOXA knock out cell line has been generated using CRISPR/Cas9 technology. This cell line lacking NOXA, has shown a great resistance to fluorizoline treatment. Therefore, fluorizoline triggers apoptosis mainly through NOXA upregulation, as BIM interference in NOXA KO cells has not shown a high resistance. Furthermore, transcriptional factors ATF4 and ATF3 are upregulated upon treatment at short times, suggesting that fluorizoline could induce ER stress, as we have observed in other cell lines.

To conclude, fluorizoline triggers apoptosis through the intrinsic pathway mainly by the upregulation of NOXA. Moreover, NOXA upregulation could be caused by ATF4 and ATF3, probably induced by an increase of ER stress.

## ECDO 37 CRISPR/Cas9 screens identify novel *Mycobacterium tuberculosis* host cell death restriction factors as potential drug targets for treatment of Tuberculosis

### Doerflinger M^1^, Stutz M^1^, Milla L^1^, Herold MJ^1^, Pellegrini M^1^

#### ^*1*^*The Walter and Eliza Hall Institute of Medical Research, Parkville VIC, Australia*

An estimated one quarter of the world’s population are infected with *Mycobacterium tuberculosis*, the causative agent of tuberculosis (TB). The skyrocketing number of multi-drug resistant TB cases illustrates that novel host-directed approaches are urgently needed to mitigate morbidity, mortality and antimicrobial resistance of *M. tuberculosis*. In order for the pathogens to infect their host target cell and manifest disease, a multitude of host cellular defense strategies have to be evaded and host cell death signaling pathways manipulated. We propose that a detailed understanding of host-pathogen interactions involved in cell death manipulation and disease progression will enable us to uncover a potential Achilles’ heel and pave the way to develop novel therapeutic intervention strategies.

Alveolar macrophages are the first line defense against infection and critical lung host cell for *Mycobacterium tuberculosis*. I have established a novel in vitro screening platform by lentivirus transduction of murine macrophages with a full genome CRIPSR/Cas9 guide library. This macrophage knockout library was infected large-scale with *M. tuberculosis* and a cell death enrichment screen performed to decipher the host factors that are manipulated to facilitate pathogen-mediated host cell death induction. Next Generation Sequencing of the barcoded resistant clones revealed 166 host resistance genes. The screen hits represent many well-known host pathways subverted by *M. tuberculosis* as well as includes well-characterized host factors involved in cell death regulation during TB such as Toll-like receptor 2 (*TLR2*), Cathepsin G (*Ctsg*) and CC-Chemokine ligand 20 (*CCL20*). Most importantly, we identified previously unknown host resistance factors including *SF3B2* that might play an important role in dysregulating host cell transcriptional splicing of cell death regulators, including the pro-survival protein *MCL1*. We are now interrogating the novel candidate genes and the relevant signaling pathways as potential therapeutic targets for cell death manipulation during TB disease both by genetic knock out and drug targeting.

## ECDO 38 α-Casein Changes Gene Expression Profiles and Promotes Tumorigenesis of Prostate Cancer Cells

### Joo-Young Kim^1^, Seong Ik Bang^2^, Sang Don Lee^1,2^

#### ^*1*^*Pusan National University Yangsan Hospital, Yangsan, Republic of Korea;*^*2*^*Pusan National University School of Medicine, Yangsan, Republic of Korea*

Prostate cancer is among the most prevalent malignancies in men and development of prostate cancer is known to be affected by environmental factors such as diet. However, up until now, the precise mechanisms by which milk and dairy products exert effects on the proliferation and progression of prostate cancer have remained unclear. In this study, we set out to examine the gene expression profiles of prostate cancer cells after treatment with the milk protein, α- casein, with the aim of determining the mechanism by which α-casein induces the proliferation and progression of prostate cancer cells.

The gene expression profiles of PC3 prostate cancer cells treated with α-casein were assessed using microarray. To clarify the molecular events involved in the effect of α-casein on proliferation and progression of prostate cancer cells, we examined cell proliferation assay, Quantitative real-time PCR, Western blotting, and immunohistochemical and immunofluorescence staining.

In this study, we used gene expression profiling to identify gene changes in PC3 cells exposed to α-casein. Our microarray data provided a genome-wide analysis of the cellular response to α-casein treatment. α-casein altered the expression of a large number of genes related to the control of growth, cell survival, and physiologic behaviors in PC3 cells. α-casein promoted PC3 cell proliferation and survival under serum-free conditions by increasing the expression of E2F1 and its target gene PCNA, suggesting that expression of E2F1 and PCNA may be associated with growth activity of α-casein in PC3 cells. Upon serum deprivation, α-casein also protected PC3 cells from serum-starved autophagic cell death by activating the PI3K/Akt pathway through activation of mTORC1, upregulation of p70S6K, and downregulation of LC3 autophagosome formation. Our data suggest that alterations in the biological processes and molecular functions in α-casein treated PC3 cells are complex, and are likely to be mediated by a variety of regulatory pathways.

These data may be helpful in determining the molecular mechanisms by which α-casein exerts its tumorigenic effects on PC3 prostate cancer cells; such information could be further exploited to devise preventive or therapeutic strategies for the treatment of prostate cancer.

## ECDO 39 Programmed Cell Death in a Model of The Nonhealing Skin Wound

### Elena I. Morgun^1,2,3^, Olga S. Rogovaya^2,3^, Ekaterina A. Vorotelyak^2,3^

#### ^*1*^*Moscow Institute of Physics and Technology (State University), Dolgoprudny, Russia;*^*2*^*Koltzov Institute of Developmental Biology of Russian Academy of Sciences, Moscow, Russia;*^*3*^*Pirogov Russian National Research Medical University, Moscow, Russia*

To study programmed cell death in a mouse model of the nonhealing skin wound.

We used balb/c mice in this study. Manipulations with mice were done under anesthetics and with acceptance of the Ethic committee. The H-shaped 10×30 mm skin flap and full-thickness circular wound 5-7 mm in diameter in the middle of the flap were inflicted onto the back of an animal. We studied programmed cell death on day 5, 7, 14 and 21 after operation by methods of histology and immunohistochemisty.

On the day 5 after an operation we observed a lot of degenerated and dead dermal cells and keratinocytes in the wound margins. Inflammatory cells were observed in the wound bed. On the day 7 cell death became more expressed. On the day 14 we observed epithelization of wound beds of 50% of mice, however dermal cells were degenerated. Wounds of other 50% of mice still weren’t healed. On the day 21 wounds of the majority of mice were epitalizated, however keratinocytes and dermal cells were disposed to cell death. Immunohistochemical assay for RIPK-1 and RIPK-3 showed positive staining of cells of the wound bed and margins on day 5, 7, 14 and 21. The most significant intensity of staining was observed on the day 14 in the cells of the granulation tissue. On the day 21 keratinocytes and hair follicle cells showed positive staining for RIPK-1 and RIPK-3.

Programmed cell death plays important role in the pathogenesis of the non-healing wound and can be a target of therapy.

Disclosure: The work was conducted under the IDB RAS Government basic research program, № 0108-2018-0004.

## ECDO 40 Sphingomyelin synthase 1: A novel regulator of TNF-induced necroptosis

### Caroline Moerke^1^, Andreas Dahl^2^, Andreas Petzold^2^, Ulrich Kunzendorf^1^, Stefan Krautwald^1^

#### ^*1*^*University Hospital Schleswig-Holstein, 24105 Kiel, Germany;*^*2*^*Technische Universität Dresden, Center for Molecular and Cellular Bioengeneering (CMCB), Biotechnologisches Zentrum (BIOTEC), 01307 Dresden, Germany*

Knowledge of the molecular mechanisms of cell death and understanding their fundamental roles in a wide range of diseases including stroke, myocardial infarction, sepsis, cancer, and in solid organ transplantation, is of major importance to clinicians. Although apoptosis has been a focus of drug discovery for many years, recent research has identified regulatory mechanisms and signaling pathways for previously undiscovered regulated necrotic cell death routines. Significant pharmaceutical research is attempting to develop therapeutic agents to target the pathways of regulated cell death modalities. Before pharmacological interference in regulated necrosis can be successfully achieved in patients, the key molecular players and mechanisms associated with individual pathways must be identified and understood.

Inflammatory pathologies are driven by TNF-induced cell death. We used a genome-scale CRISPR-Cas9 knockout (GeCKO) library to screen for unknown novel players in TNF-induced necroptosis and identified as one of the highest-ranking candidate in our setting sphingomyelin synthase 1 (*Sgms1*) as a so far undescribed mediator of this cell death modality. SGMS1 is located in the Golgi apparatus and catalyzes the last step in sphingomyelin biosynthesis, the transfer of phosphorylcholine to ceramide, producing sphingomyelin and diacylglycerol. *Sgms1* knockdown was correlated with decreased sphingomyelin levels in the lipid membrane and changes in sphingomyelin-rich membrane domains such as lipid rafts. Classical knockout of *Sgms1* conducted subsequently in vitro in different cell lines led to resistance to TNF-induced necroptosis, accompanied by absence of the phosphorylation of key regulators of the necroptotic cell death machinery, receptor-interaction protein kinase 3 (RIPK3) and mixed lineage kinase domain-like protein (MLKL). Furthermore, knockout of *Sgms1* largely suppressed activation of the nuclear factor NF-κB as well as p38 mitogen-activated protein kinase (MAPK). *Sgms1* knockout cells revealed an altered composition of lipid rafts. Because the TNF receptor translocates into lipid rafts upon TNF binding and the integrity of the lipid rafts is required for TNF receptor-mediated signaling, we next analyzed whether the knockout of SGMS1 resulted in sphingomyelin-depletion in lipid rafts. This was confirmed, and we also found that the altered lipid raft composition is not sufficient for TNF receptor integration and its signaling transduction. Using preclinical animal models, we will next analyze in vivo the therapeutic relevance of SGMS1 in the course of pathophysiological necrosis.

## ECDO 41 Genetic ablation of RIP3 protects from dopaminergic neuronal death in the MPTP-mouse model of Parkinson’s disease

### Joana D. Amaral^1^, Pedro A. Dionísio^1^, Sara R. Oliveira^1^, Cecília M. P. Rodrigues^1^

#### ^*1*^*Universidade de Lisboa, Lisbon, Portugal*

Parkinson’s disease (PD) is driven by dopaminergic neurodegeneration in the substantia nigra (SN) and striatum. Although apoptosis is considered as the main mechanism for neuronal death, other cell death pathways may be involved. In this regard, necroptosis is a regulated form of cell death dependent on receptor interacting protein 3 (RIP3) that is implicated in several neurodegenerative disorders. To explore the role of necroptosis in PD, we used wild-type (Wt) and RIP3 knockout (RIP3ko) mice intraperitoneally injected with 40 mg/kg MPTP and sacrificed 31 days after.

As expected, MPTP exposure decreased the levels of tyrosine hydroxylase (TH) staining, a marker of dopaminergic neurons, in the SN and striatum of Wt mice. Notably, RIP3ko reverted this phenotype. Intriguingly, no markers of necroptosis were found at this late time point. MPTP-treated RIP3ko mice, however, presented reduced TUNEL staining and caspase-3 activation in the SN, suggesting that neuronal apoptosis was diminished. Moreover, in vitro RIP3ko neuronal cultures exposed to MPP^+^, the toxic MPTP metabolite, were also protected from general cell death. MPTP treatment also increased astrogliosis in the striatum of Wt mice, which was further exacerbated in RIP3ko mice. Interestingly, this effect was accompanied by a reposition of glial cell line-derived neurotrophic factor (GDNF) levels in the striatum of MPTP-treated RIP3ko mice when compared to MPTP-treated Wt mice, which presented a massive decrease in GDNF levels. Of note, GDNF is a potent neurotrophic agent for dopaminergic neurons, thus suggesting a protective role for the gliosis observed in RIP3ko mice. Finally, RIP3ko mixed glial cultures exposed to pro-inflammatory stimuli showed reduced pro-inflammatory gene expression when compared to Wt.

Overall, our results highlight RIP3 as a potential therapeutic target in PD. Ablation of RIP3 protected from multiple cell death pathways in the MPTP-mouse model of PD, while potentiating a more neurotrophic and milder inflammatory phenotype in reactive astrocytes.

Acknowledgements to UID/DTP/04138/2013, SFRH/BD/102771/2014, SFRH/BPD/100961/2014, PD/BD/128332/2017, FCT, Portugal

## ECDO 42 PDK suppression due to chronic influence of NOS inhibitor blocks the development of hypoxic resistance of experimental neoplasions

### M.V. Filimonova ^1^, Т.С. Korneeva^1^, LI Shevchenko^1^, A.S. Samsonova^1^, A.S. Filimonov^1^

#### ^*1*^*Tsyb Medical Radiological Research Center, Obninsk, Russia*

Earlier in our studies, we showed that the original synthesized N, S-substituted isothiourea named T1023, an effective inhibitor of NOS, induced a marked suppression of the growth and metastasis of a number of transplanted animal tumors, caused mainly by anti-angiogenic, hypoxic action, leading to the decrease of proliferation and marked increase in apoptosis of tumor cells. But in the long term, despite stable violations of tumor vascularization and depth of hypoxia, the inhibition of tumor growth was weakened, and the activity of tumor cells apoptosis decreased. We regarded this as a manifestation of the development of hypoxic resistance of neoplasia. To overcome it, in this study, we tried to combine in one chemical compound the NOS-inhibiting and hypoxia-oriented cytotoxic activity. Isothiourea T1023 is a weak chemical base; we synthesized its derivative MLA84 in the form of a salt with dichloroacetate (DCA), which, being a PDK inhibitor, is able, by activating the respiratory chain, to induce apoptosis of tumor hypoxic cells.

The aim of this investigation was to compare antitumor activity of the NOS inhibitor T1023, its derivative MLA84 and DCA.

A comparative study of the dynamics of the solid Ehrlich carcinoma (EC) growth in female F1 (CBAxC57BL6j) mice receiving daily intraperitoneal administration of T1023 (30 mg/kg) or equimolar DCA (16.8 mg/kg) or MLA84 (35.4 mg/kg).

The antitumor effect of all investigated compounds was statistically significant from 3-5 days after the onset of treatment until the end of the observation. The effect of T1023 and DCA on EC growth was similar, and no significant differences were observed between these groups. Both compounds caused rapid inhibition of EC growth, which reached a maximum by 5-8 days and was 40-45%. But subsequently suppression of the EC growth in mice receiving T1023 and DCA progressively weakened and by the end of the experiment it was 20-25%. An essentially different dynamics of the antitumor effect was observed in the group of mice receiving MLA84. In this case, the increase in suppression of EC growth was more pronounced and prolonged. The inhibition of the EC growth reached a maximum by the 10th day from the onset of the effects, it was 50-55%, and by the end of the experiment it remained at the level of 40-45%, which corresponded to the maximum for T1023 and DCA. It is noteworthy that in late terms the antitumor effect of MLA84 statistically significantly exceeded the effects of T1023 and DCA.

The results obtained indicate that the EC is more sensitive to the action of MLA84, and no pronounced adaptation of this tumor to its effect is observed. In this connection, it is advisable to study in detail the characteristics of the antineoplastic activity of this compound.

## ECDO 43 New inhibitors of the BCL-2 family members derived from natural drimane sesquiterpenes

### Florian Daressy^1,2^; Loëtitia Favre^1^, Laura Bousquet^1^, Marc Litaudon^2^, Cécile Apel^2^, Jérome Bignon^2^, Olivier Pamlard^2^, Florian Malard^2^, Fanny Roussi^2^, Joëlle Wiels^1^, Aude Robert^1^

#### ^*1*^*UMR CNRS 8126, Univ. Paris Sud, Université Paris-Saclay, Institut Gustave Roussy, 114 rue Edouard Vaillant, 94805, Villejuif Cedex, France;*^*2*^*Institut de Chimie des Substances Naturelles, CNRS, ICSN UPR2301, Université Paris-Saclay, 91198, Gif-sur-Yvette, France*

Proteins of the BCL-2 family play a major role in cellular homeostasis since they regulate apoptotic cell death through dynamic interactions between the anti- and pro-apoptotic members. Over-expression of anti-apoptotic members (BCL-2, BCL-xL, MCL-1...) participates in the development of tumors as well as in their resistance to treatments (chemotherapy, radiotherapy). Synthetic inhibitors targeting these anti-apoptotic proteins have therefore been developed, some of which being currently in clinical trials.

Natural products possess a unique and vast chemical diversity and are therefore playing a significant role in drug discovery and development processes. Since the 1940’s, 75% of the 175 small molecules used in cancer therapy are either natural products or derivatives of natural products. Screening of plant extracts, marine organisms or microorganisms can provide highly original and functionalized bioactive molecules that are unlikely obtained by the screening of synthetic libraries.

In this context, the bio-guided screening of 9000 plant extracts led to the identification of five new families of leads that were active on anti-apoptotic members of the BCL-2 family from a micro to a sub-micromolar range. Among them, drimane sesquiterpenes isolated from *Zygogynum pancheri* were selected for further investigations. Various analogues were prepared and their interactions with BCL-XL, MCL-1 and BCL-2 proteins were first evaluated by fluorescence polarization assay (FPA). Two compounds were then tested for their cytotoxic activities (measured by MTT assay and flow cytometry analysis after Annexin-V/IP labeling) on hematological cancer cell lines whose dependency on distinct pro-survival BCL-2 family members was previously determined by BH3 profiling. The results showed that one compound is a pan-BCL-2 inhibitor while the other one selectively targets Mcl-1. Finally, we also showed that both inhibitors killed tumor cells by a BAX/BAK-mediated apoptotic process (assessed by flow cytometry labeling with conformational antibodies and western-blots on subcellular fractions) and were able to disrupt BAK/MCL-1 interaction in cells (assessed by co-IP experiments).

We are currently investigating activity of these compounds in solid tumors and the most effective and selective one will be tested in vivo, alone or in combination with genotoxic drugs, on different xenografted murine models.

## ECDO 44 Drug delivery with functionalized magnetic metal-organic framework nanoparticles

### Andrey V. Babenyshev^1^, Konstantin G. Shevchenko^1,2^, Alexey V. Yaremenko^1^, Vladimir R. Cherkasov^1^, Petr I. Nikitin^2^, Maxim P. Nikitin^1,2^

#### ^*1*^*Moscow Institute of Physics and Technology, Dolgoprudny, Russia;*^*2*^*Prokhorov General Physics Institute of the Russian Academy of Sciences, Moscow, Russia*

Nanoparticles are perspective agents for constructing of “smart” materials for different biomedical applications (Shevchenko et al., 2017; 10.1016/j.bios.2016.09.042) and for targeted drug delivery (Shevchenko et al., 2018; 10.1007/s13204-018-0659-2). Such carriers as nano- and microsized metal organic frameworks has recently provided an appealing alternative to conventional instruments. Here we show a novel approach to design of multifunctional nanoagents for targeting of cell membrane proteins by developing a magnetic core-MOF shell nanoparticles with doxorubicin and functionalized surface to target specific cell surface markers.

First, we synthetized superparamagnetic nanoparticles composed of magnetite core within porous metal organic framework shell (MP-MOF). We chose iron (III) trimesate due to its high loading capacity and feasibility of this MOF to degrade in physiological conditions (Tregubov et al., 2018; 10.1016/j.jmmm.2017.10.070). Doxorubicin was employed as a cytotoxic drug. Next, we evaluated kinetics of doxorubicin sorption and desorption from the nanoparticles, which was similar to nanosized MOF. Doxorubicin was progressively released after 2 weeks in PBS. To enhance the properties of the synthesized particles we coated them with and functionalized them with specific receptors to cell surface proteins. Using flow cytometry, we demonstrated the specify of interaction of MP-MOF with cells, while MTT assay showed the specificity of therapeutic effect. These particles can be an attractive tool for development of specific agents against clinically relevant targets for cancer treatment.

Disclosure: Different aspects of this research were supported by the Russian Foundation for Basic Research (grants No. 18-29-04065, 17-33-80176, and 17-02-01415) and by the Russian Federation President grant No. MK-5336.2018.3.

Acknowledgment: AVB and KGS contributed equally

## ECDO 45 Fabrication of Highly Specific Drug Delivery Nanoagents and Investigation of Their Interaction with Cells

### Elizaveta N. Mochalova^1,2^, Ilya L. Sokolov^1,2^, Irina L. Nikitina^2^, Alexey V. Orlov^1,2^, Konstantin G. Shevchenko^1,2^

#### ^*1*^*Moscow Institute of Physics and Technology, Dolgoprudny, Russia;*^*2*^*Prokhorov General Physics Institute of the Russian Academy of Sciences, Moscow, Russia*

Nanoparticles capable of biosensing, drug delivery, and diagnosis are expected to be an attractive tool for various biomedical applications (I.L. Sokolov et al., 2017, 10.1016/j.bbagen.2017.01.027; A.V. Orlov et al., 2018, 10.1016/j.snb.2017.11.025; S.L. Znoyko et al., 2018, 10.1016/j.aca.2018.07.012). Unique properties of nanoparticles allow their functionalization with bioreceptors and further loading with active drugs. Such theranostic agents can be used for a variety of biomedical purposes and understanding the intricacies of nanoparticle-cell interactions is essential for efficient drug delivery.

Here we synthesized a diverse set of nanoparticles functionalized with bioreceptors and investigated their interactions with both target and non-target cells. We studied structure of nanoparticle-cell complexes with SEM and observed binding and clustering of the nanoparticles on the cell surface. Using imaging flow cytometry we evaluated various parameters nanoparticle interaction with the target cells (internalization, membrane targeting, clustering, etc.). For magnetic nanoparticles their total binding to cell surface was quantified with MPQ-cytometry (magnetic particle quantification). This method allows precise direct quantification of magnetic nanoparticles bound to cells without complex sample preparation and additional labels (V.O. Shipunova et al., 2016, 10.1039/c6nr03507h; A.V. Orlov et al., 2016, 10.1021/acs.analchem.6b02066). Furthermore, using spectral phase interferometry, a separate investigation was performed to compare nanoparticle characteristics in terms of affinity to cells to their affinity to solid phase-bound ligands.

The obtained data will be useful for development of advanced nanoagents with high specificity to cellular targets.

Disclosure: Different aspects of the research were partially supported by the RFBR grants No. 16-33-60228, 18-34-00676, Program of the PRAS No. 32.

Acknowledgment: ENM and ILS contributed equally.

## ECDO 46 Development of the SPR based method for real-time monitoring of surface cell markers density

### Konstantin G. Shevchenko^1,2^, Afanasiy V. Lunin^1^, Anastasia V. Popova^1^, Evgeny L. Kolychev^1^, Boris G. Gorshkov^2^, Maxim P. Nikitin^1,2^

#### ^*1*^*Moscow Institute of Physics and Technology, Dolgoprudny, Russia;*^*2*^*Prokhorov General Physics Institute of the Russian Academy of Sciences, Moscow, Russia*

Smart nanomaterials provide unprecedented opportunities for development of various methods and instruments addressing a wide range of biomedical problems (Shevchenko et al., 2017; 10.1016/j.bios.2016.09.042). Although many systems were designed to actuate target cells, development of the instruments for localized biosensing lag behind (Shevchenko et al., 2018; 10.1007/s13204-018-0659-2). Here we show an attractive approach for real-time *in situ* biosensing of the cell surface markers and their concentration based on the shift of the surface plasmon resonance (SPR) upon specific assembly of gold nanoparticles on cell surface.

First, we developed a nanoparticle model of such biosensing system. It is self-assembled via non-covalent molecular interface from a larger magnetic nanoparticle (a “core”) and smaller gold nanoparticles (AuNPs). The close packing of the gold particles during assembly results in a shift of a surface plasmon resonance. Using UV-Vis spectroscopy we observed the significant, specific and reversible SPR shift in phosphate buffer and in cell culture medium. The range of SPR shift correlated with the density of the immobilized gold nanoparticles on the core. The proposed approach could be used for *in situ* biosensing, which can detect changes in concentration of a target cell surface markers.

Our system is attractive for use in different areas of biology and medicine with focus on cell biology, physiology and theranostics (Isaev et al., 2017; 10.1038/bmt.2017.46).

Acknowledgment: KGS and AVL contributed equally.

Disclosure: Different aspects and parts of this multidisciplinary research were partially supported by the Russian Foundation for Basic Research (grants No 18-03-01252, 16-34-60230, and 17-54-560024) and Program of the PRAS No. 40.

## ECDO 47 The role of caspase-2 in the regulation of apoptosis, necroptosis and mitotic catastrophe upon genotoxic stress in ovarian carcinoma cells

### Kopeina G.S.^1^, Zamaraev A.V.^1^, Prokhorova E.A.^1^, Egorshina A. Yu.^1^, Lavrik I.N.^1,2^, Zhivotovsky B.^1,3^

#### ^*1*^*ML Lomonosov State University, 119192 Moscow, Russia;*^*2*^*Otto von Guericke University, 39120 Magdeburg, Germany.;*^*3*^*Karolinska Institute, Box 210, SE-17177 Stockholm, Sweden;*

Caspase-2 possesses the proapoptotic and tumor suppression functions and participates in maintenance of genomic stability. Caspase-2 is known to play a critical role in apoptosis upon genotoxic stress. Moreover, several tumors (e.g. hepatocellular carcinoma, breast cancer, glioblastoma, ovarian carcinoma) are characterized by the decreased expression level of caspase-2. Despite a significant progress in our understanding of caspase-2 apoptotic and non-apoptotic functions, the mechanisms of its activation and action are still poor understood. Here, we demonstrate that caspase-2 can regulate three different modes of programmed cell death (PCD) induced by genotoxic stress.

We found that in CAOV-4 ovarian carcinoma cells genotoxic stress triggers an activation of caspase-2 in PIDDosome-independent complex which does not include the adapter protein RAIDD. Indeed, downregulation of RAIDD using siRNA did not affect caspase-2 processing or apoptotic cell death induced by cisplatin, DNA-damaging chemotherapeutic agent. Moreover, isolated high-molecular-weight (HMW) complex contained functionally active caspase-2 but not RAIDD. At the same time, the downregulation of caspase-2 using shRNA or siRNA significantly attenuated apoptotic death of CAOV-4 cells after treatment with cisplatin or doxorubicin. To analyze the role of caspase-2 in the necroptosis induction cells were treated with cisplatin in combination with the pan-caspase inhibitor zVAD-fmk. Intriguingly, downregulation of caspase-2 led to an enhanced phosphorylation of RIPK1 and MLKL and an increase of necroptosis. Taken together, our data strongly indicate that caspase-2 initiates apoptosis and negatively regulates necroptotic cell death upon genotoxic stress.

Cellular localization of caspases influences their function and plays a crucial role during PCD. Previously, caspase-2 was shown to possess a nuclear localization signal and could be localized in the nucleus. Interestingly, we found caspase-2 probably in the ubiquitinated form in the nucleus. Interestingly, accumulation of caspase-2 in the nucleus was detected not only under genotoxic stress, but also after TNF treatment. Further study of caspase-2 nuclear functions revealed that the low concentration of DNA-damaging agent doxorubicin induced caspase-2-dependent cell death which was preceded by mitotic catastrophe. Moreover, phosphorylation of histone H2AX – one of the main hallmarks of DNA damage, strongly correlated with caspase-2 expression level and significantly reduced in caspase-2-deficient cells treated with doxorubicin. Thus, upon genotoxic stress caspase-2 regulates three modes of PCD, apoptosis, necroptosis and mitotic catastrophe and influences molecular pathway(s) responsible for DNA damage detection.

Disclosure: This work was supported by the grant from the Russian Science Foundation (17-75-20102)

## ECDO 48 Suppression of glycolysis and its consequences for tumor cell elimination

### Polina Maximchik^1^, Alibek Abdrakhmanov^1^, Evgeniya Inozemtseva^1^, Boris Zhivotovsky^2^, Vladimir Gogvadze^2^

#### ^*1*^*MV Lomonosov Moscow State University, 119991, Moscow, Russia;*^*2*^*Karolinska Institutet, Box 210, Stockholm, SE-171 77 Sweden*

The dependence of tumors on glycolysis for ATP generation offers a rationale for therapeutic strategies aimed at selective inhibition of the glycolytic pathway. 2-Deoxyglucose (2-DG), a non-metabolizable glucose analog, inhibits phosphorylation of glucose by hexokinase in a competitive manner and mimics glucose deprivation conditions, decreasing glucose-6-phosphate level and glycolytic capacity. However, it is also known that 2-DG blocks N-linked glycosylation, leading to ER stress and an unfolded protein response (UPR) in many cell types, which may be followed by cell dysfunction and death. Therefore, we decided to investigate how the outcome of co-treatment with therapeutic drugs and modulators of glycolysis will be determined by multiple factors and their interplay.

Using a set of experimental approaches (western-blot analysis, measurement of caspase activity and Annexin V-PI staining) a comparison of neuroblastoma SK-N-BE(2) and HCT116 cells was performed and revealed that in SK-N-BE(2), but not in HCT116 cells, 2-DG alone could induce apoptosis. In contrast, in HCT116, 2-DG induced not apoptosis, but autophagy. In SK-N-BE(2) cells, the decrease in ATP levels upon treatment with 2-DG was more prominent, because in HCT116 cells mitochondria were able to compensate for the loss of ATP caused by glycolysis suppression. In both cells lines 2-DG triggered endoplasmic reticulum stress, assessed by the accumulation of the marker protein GRP78/BiP. Suppression of ER stress by mannose attenuated the 2-DG-induced apoptotic response in SK-N-BE(2) cells, implying that apoptosis in these cells is a consequence of ER stress induction. In HCT116 cells, ER stress stimulated autophagy, assessed by the accumulation of the lipidated form of LC3. The inhibition of ER stress by mannose suppressed autophagy and reversed cisplatin-induced apoptosis suppression caused by 2-DG. When autophagy in HCT116 cells was suppressed by bafilomycin, apoptosis was decreased. At the same time, stimulation of autophagy in SK-N-BE(2) cells attenuated cell death.

We found that co-treatment with 2-DG and cisplatin can either stimulate or suppress cell death in various cancer cell lines. The outcome of the treatment with combination of 2-DG and cytotoxic agents depends on the interplay between ER stress, autophagy and apoptosis. Thus, successful treatment of tumors with conventionally used anticancer drugs should be combined with targeting metabolic pathways involved in the regulation of apoptosis, autophagy, and cellular bioenergetics.

Disclosure: This work was supported by a grant from the Russian Foundation for Basic Research (18-315-00327) and the Russian Science Foundation (14-25-00056).

## ECDO 49 Modulation of Mcl-1 transcription by serum deprivation sensitizes cancer cells to cisplatin

### Viacheslav V. Senichkin^1^, Gelina S. Kopeina^1^, Inna N. Lavrik^1,2^, and Boris Zhivotovsky^1,3^

#### ^*1*^*Faculty of Medicine, MV Lomonosov Moscow State University, Moscow, Russia;*^*2*^*Otto von Guericke University, Magdeburg, Germany;*^*3*^*Karolinska Institutet, Stockholm, Sweden*

One of the most commonly exploited strategies in anticancer treatment is based on the use of DNA-damaging agents which trigger apoptotic death of cancer cells. However, these widely used drugs have significant toxic effects on normal cells, which also undergo apoptosis upon treatment. Thus, development of approaches that increase therapeutic effects of anticancer drugs is an important task of oncomedicine. The combination of calorie restriction (CR) and the chemotherapeutic drugs has been shown to enhance programmed death of cancer cells that will allow decreasing the dose of chemotherapy drugs and reducing their toxic effects. Here we examined the mechanisms of how serum deprivation (SD), which serves as an in vitro model of CR, modulates apoptosis in cancer cell lines (ovarian carcinoma Caov-4 and cervical adenocarcinoma HeLa) in response to treatment with DNA-damaging chemotherapeutic agent, cisplatin. SD enhanced apoptotic death of cancer cells in combination with cisplatin in time-dependent manner documented by Annexin V/Propidium iodide staining and PARP cleavage. Our studies have demonstrated that Mcl-1 – one of the key apoptotic regulators – underwent rapid degradation in response to the combinatorial treatment that promoted apoptosis induction through more rapid caspase-9 activation. Enhanced degradation of Mcl-1 upon combinatorial treatment occurred upstream of caspases. The crucial role of Mcl-1 degradation in sensitization of cancer cells to cisplatin upon SD was confirmed using siRNA approach. In contrast, in cells with relatively low basal level of Mcl-1 (ovarian carcinoma line Skov-3) the treatment with cisplatin alone led to the efficient degradation of Mcl-1. Consistently, no increased PARP cleavage upon combinatorial treatment was detected in these cells. This also emphasizes the important role of Mcl-1 in SD-mediated sensitization towards cisplatin-induced cell death. It was shown that Mcl-1 undergoes proteasomal degradation upon cisplatin administration. At the same time, direct influence of SD on the proteasomal degradation of Mcl-1 protein was not observed. Instead, SD led to downregulation of Mcl-1 mRNA that resulted in rapid drop of Mcl-1 level in cisplatin-treated cells. Taken together, these data demonstrate that decreased transcription of Mcl-1 gene is one of the mechanisms through which SD increases cell death of cancer cells that could be applied in anticancer therapy.

Disclosure: This work was supported by Russian Science Foundation (grant 14-25-00056)

## ECDO 50 Glutamine addiction and etoposide exposure effect on neuroblastoma treatment

### Kadri Valter^1^, Polina Maximchik^2^, Boris Zhivotovsky^1,2^, and Vladimir Gogvadze^1,2^

#### ^*1*^*Karolinska Institutet, Stockholm, Sweden;*^*2*^*Moscow State University, Moscow, Russia*

Tumor cell elimination through stimulation of mitochondrial pathways in apoptosis seems to be a promising strategy. Compounds that target mitochondria can suppress mitochondrial activity or contribute to the outer mitochondrial membrane permeabilization leading to apoptosis. Various therapeutic drugs are DNA-damaging agents; and whether or not they can directly target mitochondria remains unclear. In addition, some tumors display addiction to glutamine despite the fact that it is a nonessential amino acid. Attained glutamine is converted by glutaminase into glutamate, which is used as an important pre-substrate for the Krebs cycle, synthesis of glutathione, amino and fatty acids to support intensive proliferation of cancer cells. One of such glutamine-dependent tumors is neuroblastoma (NB), most common solid cancer in childhood. Our aim was to evaluate the direct effect of widely used therapeutic drugs on mitochondrial activity in combination with glutamine withdrawal, and possible apoptotic effects of such interaction.

Our results revealed that etoposide caused a rapid decrease in oxygen consumption. However, the inhibition of respiration was reversible and restored in a time- and concentration-dependent manner. Further studies demonstrated a severe suppression of complex I after adding etoposide, whereas no immediate effect of the drug on complex II was detected. However, the assessment of respiration after 6h and 18h etoposide treatment revealed that complex I activity has recovered to normal level. Blocking of the complex I can cause a leakage of electrons and formation of reactive oxygen species (ROS). Therefore, the assessment of mitochondrial superoxide production upon etoposide administration was performed, revealing sudden ROS formation. However, apoptotic manifestation was detectable only upon withdrawal of glutamine. Thus, depletion of the antioxidant glutathione together with the destabilization of mitochondria by ROS can compromise barrier properties of mitochondrial membrane leading to cytochrome c release and activation of mitochondrial apoptotic pathway.

In conclusion: in addition to targeting DNA, etoposide can stimulate cell death by ROS due to the leakage of electrons through inhibition of complex I of the mitochondrial respiratory chain. However, powerful mitochondrial antioxidant defence system protects cells and prevents cell death. Thus, the depletion of antioxidants or inhibition of pathways responsible for cellular antioxidant response can make targeting mitochondria more efficient, enhances and strengthens antitumor therapy.

Disclosure: This work was partially supported by Russian Science Foundation (grant number 14-25-00056). The work in the authors’ laboratories is supported by the Stockholm and Swedish Cancer Societies, the Swedish Childhood Cancer Foundation, the Swedish Research Council.

## ECDO 51 Structure comparative analysis of phosphorylation sites in caspases

### Alexey V. Zamaraev^1^, Gelina S. Kopeina^1^, Boris Zhivotovsky^1,2^, Inna N. Lavrik^1,3^

#### ^*1*^*MV Lomonosov Moscow State University, 119991 Moscow, Russia;*^*2*^*Karolinska Institute, Box 210, 17177 Stockholm, Sweden;*^*3*^*Otto von Guericke University, Magdeburg 39106, Germany;*

Phosphorylation is one of caspase posttranslational modifications in which phosphorylation of serine, threonine or tyrosine residues takes place. Phosphorylation can cause conformational changes in the structure of caspases or directly affect the activity of their active centers. The number of well-known caspase phosphorylation sites is quite small, in comparison with other intracellular enzymes. Therefore, the discovery of new phosphorylation sites in caspases and analysis of the mechanisms of their phosphorylation require further detailed investigation. This in turn would allow to understand new mechanisms of programmed cell death.

To study this type of regulation of cell death we decided to perform a comparative analysis of the conservative serine, threonine and tyrosine residues in the caspases among vertebrates and invertebrates. In addition, the positions of all residues were examined by Chimera software on the tertiary structure of caspases and the correlation with the already known phosphorylation sites was performed. The following isoforms of human caspases were used: caspase-2L *(P42575)*, caspase-8a *(Q14790; pdb id:4jj7)*, caspase-9 *(P55211; pdb id:3v3k)*, caspase-3 *(P42574; pdb id:4pry)*, caspase-7 *(P55210; pdb id:4fdl)*, caspase-6 *(P55212; pdb id:3od5)* and caspase-1 *(P29466; pdb id:3e4c)*. Multiple alignment has been performed by ClustalO software. The analysis of multiple alignment has shown that phosphorylation sites, which were experimentally proven, are highly conserved. Moreover, some of them, such as Thr-263 in caspase-8 and Ser-183 in caspase-9 are located in the same position in ​​the alpha-helix in the immediate vicinity of the active center. Analysis of the alpha-helix regions located in the similar part of the tertiary structure in the different caspases demonstrated the same location of the threonine or serine residues Ser-220, Thr-67, Thr-67, Thr-90 in caspase-2, -3, -6, and -7, respectively. Moreover, fascinating is the presence in different organisms of the highly conserved phosphorylation sites Ser-347 of caspase-8, Ser-150 of caspase-3 and Thr-173 of caspase-7 at the same spatial position of the alpha-helix in the large catalytic subunit. Analysis of the similar position in caspases, in which this site was not identified, revealed the presence of putative phosphorylation sites exactly at the same place. These positions are highly conserved and involve Ser-274 of caspase-9, Ser-150 of caspase-6 and Ser-272 of caspase-1.

Thus, it was hypothesized that these sites could participate in the modification of caspases and the regulation of proteolytic activity of the enzyme. Indeed, a comparative analysis of caspase sequences and their spatial structures permitted us to identify potential phosphorylation sites. Moreover, preliminary experiments showed the involvement of these sites in the regulation of caspase activation and programmed cell death.

Disclosure: This work was supported by the grant from the Russian Science Foundation (17-75-20102).

## ECDO 52 Implication of p53 transcriptional function in the repression of PINK1-dependent mitophagy control

### Alves da Costa, C.^1^, Goiran, T.^1^, Duplan, E.^1^, Rouland, L.^1^, El Manaa, W.^1^, Lauritzen, I.^1^, Dunys, J.^1^, You, H^2^. Checler, F^1^

#### ^*1*^*Université Côte d’Azur, INSERM, CNRS/UMR7275, IPMC, team labeled “Laboratory of Excellence (LABEX) Distalz”, 660 route des Lucioles, 06560, Sophia-Antipolis, Valbonne, France;*^*2*^*Xiamen University, Xiamen, Fujian 361102, China*

p53 is a multifaceted tumor suppressor protein involved in key cellular responses that can both activate and repress autophagy. This dual effect is directly driven by its subcellular distribution. Thus, nuclear p53 elicits a pro-autophagy phenotype via the transcriptional activation of pro-autophagy genes while cytoplasmic p53 represses autophagy independently of its transcription factor function, via the activation of the AMPK-dependent inhibition of mTOR signaling cascade.

Our work completed this rather simplistic view by demonstrating that p53 can also repress mitophagy by controlling PINK1, a key pro-mitophagy protein that grants mitochondrial homeostasis. Thus, we demonstrated that nuclear p53 induces the transcriptional repression of PINK1 promoter activity, protein and mRNA levels, *ex-vivo* and in vivo. We showed that PINK1 behaves as a direct transcriptional target of p53 since the deletion of a p53-responsive element on PINK1 promoter impacts p53-mediated *PINK1* transcriptional repression. This conclusion was further validated by DNA binding assay that demonstrated a physical interaction of p53 with PINK1 promoter. Accordingly, p53 knockout enhances LC3 maturation as well as optineurin and NDP52 autophagy receptors expression and down-regulates TIM23, TOM20 and HSP60 mitophagy markers, ex-vivo and mice brain. Finally, we show that the p53-mediated negative regulation of autophagy is PINK1-dependent. Hence, pifithrin-α-mediated blockade of p53 transcriptional activity like p53 genetic invalidation lead to increased mitophagy (augmentation of LC3 maturation and reduced p62, TIM23, TOM20 and HSP60 protein levels), a response that is fully abolished by PINK1 depletion. Overall, our data describes a novel signaling circuit by which nuclear p53 can repress autophagy/mitophagy. This pathway could well be impacted in both neurodegenerative and cancer, two pathological conditions where p53 expression and transcriptional activity are drastically altered.

## ECDO 53 Gambogic acid triggers paraptosis in cancer cells via disruption of thiol proteostasis

### Min Ji Seo^1^, Dong Min Lee^1^, In Young Kim^1^, Dong Joo Lee^1^, Min-Koo Choi^2^, Joo-Youn Lee^3^, Seok Soon Park^4^, Seong-Yun Jeong^4^, Eun Kyung Choi^4^, Kyeong Sook Choi^1^

#### ^*1*^*Department of Biomedical Science, Ajou University Graduate School of Medicine, Suwon, Korea;*^*2*^*Dankook University, Cheoan, Korea;*^*3*^*Korea Research Institute of Chemical Technology, 141 Gajeong-ro, Yuseong-gu, Daejeon, Korea;*^*4*^*University of Ulsan College of Medicine, Seoul 05505, Korea*

The anticancer activity of GA, a xanthonoid extracted from the resin from Garcinia hanburyi, was recently reported in many studies, but its underlying mechanism still needs to be clarified. We show here that GA induces the cancer cell death accompanied by vacuolation not only in vitro but also in vivo. GA-induced vacuolation in various cancer cells was derived from the dilation of the endoplasmic reticulum (ER) and mitochondria, which was blocked by cycloheximide, suggesting that GA kills cancer cells via induction of paraptosis. We found that megamitochondria formation due to the fusion of swollen mitochondria preceded the fusion of ER-derived vacuoles. While GA-induced proteasomal inhibition contributes to the ER dilation and ER stress, mitochondrial membrane depolarization was followed by megamitochondria formation. Interestingly, GA-induced paraptosis was effectively blocked by various thiol-containing antioxidants, independently of ROS generation. We observed that GA can react with cysteinyl thiol to form Michael adducts, suggesting that the ability of GA to covalently modify nucleophilic cysteinyl group of the proteins may cause protein misfolding and accumulation of misfolded proteins within the ER and mitochondria. Collectively, our findings that disruption of thiol proteostasis may critically contribute to the anti-cancer effects of GA via induction of paraptosis.

Acknowledgment: MJS and DML contributed equally.

## ECDO 54 BAX activation for apoptosis: mutations near its proposed non-canonical BH3 binding site reveal allosteric changes controlling mitochondrial association

### Michael A. Dengler^1^, Adeline Y. Robin^1^, Leonie Gibson^1^, Mark X. Li^1^, Jarrod J. Sandow^1^, Andrew I. Webb^1^, Sweta Iyer^1^, Dana Westphal^1^, Grant Dewson^1^, Jerry M. Adams^1^

#### ^*1*^*The Walter and Eliza Hall Institute* of *Medical Research, 1 G Royal Parade, Parkville, Melbourne, Victoria 3052, Australia*

To elicit apoptosis, BAX metamorphoses from an inert cytosolic monomer into homo-oligomers that permeabilize the mitochondrial outer membrane (MOM). A long-standing puzzle is that BH3 domains apparently activate BAX via not only its canonical groove but also a proposed site involving the distant helices α1 and α6. However, to date, BAX mutations supporting a role for the non-canonical β1/β6 site have been lacking, as has understanding of the respective roles of this site and the groove site. Hence, we have sought to identify BAX mutations that impair its activation through the β1/β6 site and to explore whether either the groove or β1/β6 site suffices to drive full activation of BAX, or whether engagement of each site drives separate steps.

Our studies with two canonical groove mutants revealed that late steps of BAX activation like dimerization and oligomerization require activation through the groove but probably not earlier steps like MOM association and integration. Conversely, we could readily disulphide- link cysteine-tagged BH3 peptides, as well as full-length tBID, near the modeled alternative binding site, and obstructive labeling of helices α1 and α6 with PEG-maleimide strongly implicated the central regions of those helices early in BAX activation. Indeed, an alanine mutagenesis scan of both helices allowed us to characterize the first mutations in α1 or α6 impairing BID-induced BAX activation. These mutations reduced linkage of tBID to BAX and altered the BAX conformation; their exposure of the BAX α1-α2 loop allosterically sequestered its distant α9 membrane anchor in the groove, reducing MOM association. The crystal structure of an α6 mutant revealed additional allosteric effects within BAX controlling sequestration of α9 and therefore BAX MOM translocation. Together, our results suggest that the α1/α6 region can drive MOM association and integration, whereas groove binding favors subsequent steps towards oligomerization.

## ECDO 55 Interplay of Fas-ligand, caveolin-1 and Fyn kinase

### Xenia A. Glukhova^1^, Julia A. Trizna^1^, Olga V. Proussakova^1^, Igor P. Beletsky^1^

#### ^*1*^*Russian Academy of Sciences, Puschino, 142290, Russia*

Fas-ligand/CD178 belongs to the TNF family proteins; it can induce apoptosis through death receptor Fas/CD95 or by the reverse signaling pathway. It has been demonstrated earlier that Fas-ligand can bind SH3 domain of Lck and Fyn kinases as well as caveolin-1 can bind the same Src kinases. To check for relations between Fas-ligand, caveolin-1 and Fyn kinase we produced HeLa cells with tetracycline-inducible Fas-ligand expression and used it for analysis of kinase activity and protein association. The pooled raft-containing and non-raft fractions were incubated with purified Fc-fusion proteins or recombinant Fc-fragment (as a control) and bound proteins were separated by SDS-PAGE and subjected to Western-blot analysis. *In* order to investigate a possible role of p59Fyn kinase in formation of caveolin-1-Fas-ligand complexes the activity and content of the enzyme in cells were analyzed upon activation of Fas-ligand expression and cell death. Using antibodies to N-terminal (1-132 аа) fragment and to negative regulatory site of p59Fyn kinase, providing information on protein amount and its activity respectively, we showed that expression and activity of p59Fyn kinase in dying cells decreased dramatically. Additional data on the role of p59Fyn kinase in caveolin-1-Fas-ligand complex formation were obtained using a recombinant protein consisting of intracellular part of Fas-ligand fused with Fc-fragment of human IgG1 (IcFasL-Fc). Incubation of recombinant IcFasL-Fc with raft and non-raft fractions of control and induced cells demonstrated that in control cells IcFasL-Fc formed a complex with p59Fyn, but not with caveolin-1. In induced cells, p59Fyn- IcFasL-Fc complex was not detected; however IcFasL-Fc was able to bind сaveolin-1. Thus, under normal conditions, Fyn kinase (or any other Src kinase) interacts with Fas-ligand, most likely through its PRD, and caveolin-1, causing their phosphorylation. Stimulation of Fas-ligand - dependent cell death leads to dissociation of Fyn kinase- Fas-ligand and Fyn kinase-caveolin-1 complexes, dephosphorylation of Fas-ligand and association of Fas-ligand and caveolin-1.

## ECDO 56 New insights into TNF-mediated immunoregulation

### Sergei A. Nedospasov^1^

#### ^*1*^*Engelhardt Institute of Molecular Biology and Lomonosov Moscow State University, Moscow, Russia*

TNF is a multifunctional cytokine that can transmit its intracellular signals through two distinct TNF receptors, TNFR1 and TNFR2. Since TNF- and TNFR1- (but not TNFR2-) deficient mice showed very similar phenotypes, the significance of TNFR2 signaling in health and disease remained incompletely understood for a long time. To definitively ascertain TNFR2 functions in vivo, in particular, in Treg compartment, we used novel mice with humanization of TNF-TNFR system and with conditional ablation of TNFR2 in FoxP3+ cells. TNFR2 expressed on Tregs plays a protective role in experimental autoimmune encephalomyelitis (EAE) through the control of autoreactive Th17 cells. Interestingly, in several disease models TNF produced by T cells is protective, while TNF produced by myeloid cells may be pathogenic. Therefore, we are evaluating bi-specific antibodies that specifically neutralize bioactivity of TNF produced by myeloid cells. Overall, our findings mechanistically explain how the same molecule can play opposite roles depending on the context.

Disclosure: This work is supported by the Russian Science Foundation grant №14-50-00060.

## ECDO 57 Boosting of the cytotoxic effect of RNAs by using the pro-apoptotic protein lactaptin for delivery of nucleic acids into cancer cells

### Golubitskaya E. ^1,2^, Chinak O.^1^, Pyshnaya I.^1^, Stepanov G.^1^, Juravlev E.^1^, Richter V.^1^, Koval O.^1,2^

#### ^*1*^*Institute of Chemical Biology and Fundamental Medicine, Novosibirsk, Russia;*^*2*^*Novosibirsk State University, Novosibirsk, Russia*

Cell penetrating peptides (CPP) are promising agents for transporting diverse cargo into the cells. Amino acid sequence and the mechanism of lactaptin entry into the cells allow referring it to CPP group. Lactaptin, the fragment of human milk kappa-casein, and recombinant lactaptin (RL2) were initially discovered as molecules that induced apoptosis of cultured cancer cells and did not affect non-malignant cells. Here, we analyzed the lactaptin potency to form complexes with nucleic acids and to act as a gene delivery system. Plasmid DNA with EGFP gene was used for in vitro studies. We have demonstrated that lactaptin formed positively-charged noncovalent 110 nm-sized complexes with plasmid DNA. Ca^2+^ and Mg^2+^ ions stabilized these complexes while polyanion heparin displaced DNA from the complexes. The delivery of functional nucleic acids was assessed by fluorescent microscopy using A549 lung adenocarcinoma cells. We observed RL2:pDNA complexes provided GFP expression in the treated cells that strongly confirmed the entering pDNA into the cells. The efficiency of cell transformation by these complexes increased when RL2:pDNA ratio increased. RL2 didn’t decrease cell viability under using concentration. We have also examined RL2-mediated delivery of RNA molecules using anti-EGFP siRNA. A549 cells were co-transfected with GFP-expressed pDNA and RL2:siRNA complexes, and decrease of GFP in RL2:siRNA-treated cells was detected.

We have previously demonstrated that some RNAs, for example U25 snoRNA, can decrease cell viability [1]. Complexes of RL2 with U25 snoRNA were prepared for transformation of cancer cells. We have shown that these complexes boost cytotoxic effect more than U25 snoRNA or RL2 alone. The cytotoxicity of such complexes in part was due to the activation of inflammation-involved genes.

Collectively these findings prove lactaptin as delivery system for nucleic acids. Moreover, under special conditions, complex of lactaptin with RNAs can be a double-active cytotoxic compound.

Disclosure: The work was supported by Russian State funded budget project (VI.62.1.5, 0309-2016-0003) and Russian Foundation for Basic Research (Grant 16-34-60136).

## ECDO 58 Response of three types of cancer cells to photodynamic treatment assessed by means of digital holographic microscopy

### A.V. Belashov^1^, A.A. Zhikhoreva^1,2^, D.A. Rogova^1,3^, N.A. Avdonkina^4^, I.A. Baldueva^4^, *A.B. Danilova*^*4*^, *M.L. Gelfond*^*4*^, T.L. Nekhaeva^4^, I.V. Semenova^1^, O.S. Vasyutinskii^1^

#### ^*1*^*Ioffe Institute, St. Petersburg, 194021, Russia;*^*2*^*ITMO University, St. Petersburg, 197101, Russia;*^*3*^*Peter the Great St. Petersburg Polytechnic University, 195251, Russia;*^*4*^*N.N. Petrov National Medical Research Center of Oncology, Pesochnyi, St. Petersburg 197758, Russia*

Photodynamic therapy (PDT) is a promising modality applied for treatment of local and systemic cancer. A cell death pathway induced by PDT is very important for immune system activation. However currently there are controversies on optimal pathway of cell death under PDT. In this research we used digital holographic microscopy and tomography to differentiate pathways of cells death induced by PDT in melanoma (Mel), soft tissue sarcoma (STS) and renal cell carcinoma (RCC) cultures and to assess differences in PDT efficacy.

The cultures were irradiated by a semiconductor laser at the wavelength of 660nm for the induction of intracellular response. Standard doses (100 mW/cm^2^ during 7 minutes) and low doses (≈25 mW/cm^2^ during 7 minutes) of irradiation were applied. Cell cultures were monitored during 1.5 hours after photodynamic treatment, holograms were taken every five minutes at several areas of the Petri dish. PDT efficacy was evaluated by calculations of the average phase shift introduced by individual cells before and after photosensitized generation of intracellular singlet oxygen. Digital holograms of the cells were recorded in the inverted holographic microscope constructed in off-axis Mach-Zehnder configuration. Phase images were obtained by numerical processing of the recorded holograms. Morphological parameters of cells before and after treatment were obtained from three-dimensional distributions of refractive index obtained using holographic tomography. Changes of cellular morphology were analyzed and attributed to a cell death pathway.

Irradiation of the samples at the fluence rate of 100 mW/cm^2^ during 7 minutes resulted in the decrease of average phase shift in RCC (from 1.32 to 1.17, p<0.01) and STS (from 1.21 to 0.93, p<0.01) cells while in Mel cells no significant changes of this parameter were recorded (from 0.98 to 0.94). The morphological changes detected in RCC and STS at these treatment parameters indicated cells death through the necrosis pathway. At lower irradiation doses morphological changes typical for apoptosis were observed in RCC cells, including cell rounding and blebbing. In conclusion in this study we have shown significant PDT resistance of Mel cells in comparison with RCC and STS by means of digital holographic microscopy.

Disclosure: The reported study was funded by RFBR under the research project # 18-32-00364.

## ECDO 59 Quantification of cell death dynamics at photodynamic treatment in vitro using digital holographic microscopy and tomography

### Irina Semenova^1^, Andrey Belashov^1^, Anna Zhikhoreva^1,2^, Tatyana Belyaeva^3^, Elena Kornilova^3^, Anna Salova^3^, Oleg Vasyutinskii^1^

#### ^*1*^*Ioffe Institute, St. Petersburg, 194021, Russia;*^*2*^*ITMO University, St. Petersburg, 197101, Russia;*^*3*^*Institute of Cytology of RAS; St. Petersburg, 194064, Russia*

Investigations of photodynamic (PD) treatment efficacy at the cellular level are aimed at identification of cell death pathways and determination of corresponding treatment doses. The methods commonly used for distinction between apoptotic and necrotic cell behavior are based on the assessment of cell membrane integrity using standard test assays and further analysis by confocal fluorescent microscopy or on the determination of morphological changes of cells by means of flow cytometry or conventional light microscopy and transmission electron microscopy. These methods however either do not allow for monitoring cellular changes in dynamics, or provide limited quantitative data on cells condition and morphology. The technique of digital holographic microscopy (DHM) was already demonstrated to be very informative in research of various processes at the cellular level. Three-dimensional distributions of cellular parameters can be obtained by means of digital holographic tomography (DHT). DHM and DHT provide quantitative information on cells morphology and optical characteristics and allow for monitoring morphological changes in cells in dynamics and without usage of any dyes. However by now these techniques were not yet applied for studies of cellular response to PD treatment.

In this communication we present results of DHM- and DHT-assisted monitoring of changes in morphological characteristics of cells from the two cultured cancer cell lines, HeLa and A549. Cell cultures were subjected to PD treatment in vitro with Radachlorin photosensitizer at various irradiation doses. The results obtained demonstrated four substantially dissimilar scenarios of cells response to treatment, which can be identified as: no response at low doses, apoptosis at elevated doses, secondary necrosis at higher doses and immediate necrosis at high doses. The two cell types were shown to be differently responsive to treatment at the same doses. Although the sequence of death scenarios with increasing irradiation dose was demonstrated to be the same, each specific scenario was realized at substantially different doses. Results obtained by holographic microscopy were confirmed by confocal fluorescence microscopy with the AO/EB test assay.

Thus the observed dose-dependent post-treatment dynamics of cellular morphology allowed for distinguishing between several pathways of cell death. DHM and DHT were demonstrated to be promising tools for estimation of cellular response to external stimuli allowing for quantification of cellular morphology in dynamics.

Disclosure: The reported study was funded by Russian Science Foundation under the research project # 14-13-00266.

## ECDO 60 Targeting Mcl-1 is a promising strategy to kill mantle cell lymphoma cells

### Michael A. Dengler^1^, Charis E. Teh^1^, Marco J. Herold^1^, Andrew W. Roberts^1^, Jerry M. Adams^1^

#### ^*1*^*The Walter and Eliza Hall Institute of Medical Research, 1 G Royal Parade, Parkville, Melbourne, Victoria 3052, Australia*

Aberrant expression of apoptosis-regulating Bcl-2 family members can promote malignant transformation and resistance to conventional therapies, engendering intense interest in the prospects for targeting the Bcl-2 family with small molecules termed BH3 mimetics. Direct inhibition of Bcl-2 itself using the Bcl-2-specific antagonist ABT-199 (venetoclax) has already shown great efficacy with several hematologic malignancies in the clinic. Pro-survival Bcl-2-relative Mcl-1 is also deregulated in many blood cancers and hence may well also prove an important potential therapeutic target. We have assessed its potential in vitro for treating mantle cell lymphoma, an aggressive form of non-Hodgkin lymphoma associated with a very poor prognosis and rarely curable with current therapies.

To establish genetically whether Mcl-1 is critical for maintaining mantle cell lymphoma survival, we first tested the impact of acute Mcl-1 knockout using an inducible CRISPR/Cas9 system. Remarkably, lowering Mcl-1 by inducing *MCL1*-sgRNA expression triggered spontaneous apoptosis in most mantle cell lymphoma cell lines tested, endorsing Mcl-1 as a promising target. To explore the potential of directly inhibiting Mcl-1, we next exposed a panel of the cell lines to the new specific Mcl-1 inhibitor S63845. Four of five cell lines tested indeed proved sensitive to the inhibitor, which induced cell death at relatively low concentrations as a single agent. Furthermore, the efficacy of Mcl-1 inhibition could be enhanced by combined treatment of S63845 with venetoclax or the Bruton’s tyrosine kinase inhibitor ibrutinib.

To confirm these results in a more clinically relevant setting, we also tested the efficacy of the Mcl-1 inhibitor in primary samples from more than ten mantle cell lymphoma patients, using CD40L-expressing feeder cells to mimic and study potential effects of the microenvironment. Notably, all unstimulated primary mantle cell lymphoma samples were also sensitive to S63845. Interestingly, however, the CD40L-stimulation attenuated their sensitivity. Using mass cytometry (CyTOF) to explore the underlying mechanisms of CD40L-mediated resistance to S63845 at the single cell level revealed that the CD40L-stimulation strongly up-regulated pro-survival Bcl-2-relative Bcl-xL. Nevertheless, co-treatment of the stimulated cells with other BH3 mimetics, such as venetoclax and especially the Bcl-xL-specific inhibitor A-1331852, restored sensitivity to Mcl-1 inhibition. Also, pre-treatment with the Bruton’s tyrosine kinase inhibitor ibrutinib partly re-sensitized the stimulated primary tumour cells to S63845.

Together, our findings strongly suggest that Mcl-1 is crucial for maintenance of mantle cell lymphoma. Our results support clinical trial of Mcl-1 inhibitors as single agents, and, if tolerable, also its combination with other targeted therapies such as venetoclax or ibrutinib.

## ECDO 61 Neonatal lethality and inflammatory phenotype in new transgenic mice with overexpression of human interleukin-6 in myeloid cells

### E.А. Gorshkova^1^, R. V. Zvartsev^1^, М. А. Nosenko^1^, V. S. Gogoleva^1^, K. V. Korneev^1^, D. V. Kuprash^1^, А. V. Deikin^2^, M. S. Drutskaya^1^, S.А. Nedospasov^1^

#### ^*1*^*Russian Academy of Sciences, and Lomonosov Moscow State University Moscow, Russia;*^*2*^*Russian Academy of Sciences, Moscow, Russia;*

IL-6 is a pleiotropic cytokine involved in regulation of inflammation, metabolism and tumorigenesis. Using loxP-Cre system we generated mice with transgenic overexpression of human IL-6 and reporter fluorescent protein EGFP in cells of macrophage-monocyte lineage. High level of hIL-6 production by macrophages and monocytes, as confirmed in vitro in primary bone marrow-derived macrophages, resulted in vivo in early postnatal death. hIL-6 overexpression by myeloid cells led to their increased numbers in periphery and defective erythropoiesis. In order to circumvent this early lethality we have also generated mice with tamoxifen-inducible overexpression of hIL-6 in CXCR3+ myeloid cells in vivo. Phenotypic features of both types of mice will be presented. Additionally, using reverse genetics we are studying the role of IL-6 in physical endurance and cell metabolism.

Disclosure: Supported by grant 14-25-00160 from the Russian Science Foundation.

## ECDO 62 Molecular Characterization of SMAC Mimetic-Sensitive and -Resistant B-Cell Precursor Acute Lymphoblastic Leukemia

### Zinngrebe J^1^, Meyer M^1^, Meyer LH^1^ and Debatin KM^1^

#### ^*1*^*Ulm University, Ulm, Germany*

Pediatric acute lymphoblastic leukemia (ALL) is one of the most common malignancies in childhood. Survival rates increased over the past decades, but the prognosis for patients with relapsed ALL or ALL resistant to conventional chemotherapy remains poor. Thus, novel therapeutic options are urgently required. The family of inhibitor of apoptosis proteins (IAPs) has been shown to play an important role in the prevention of cell death, and to mediate gene activation important for cell survival. IAP antagonists, also known as SMAC Mimetics (SMs), were developed to counteract IAPs’ function. SMs have been shown to induce cell death in a number of different cancer entities, including B-cell precursor (BCP)-ALL.

We analyzed the intrinsic sensitivity of patient-derived BCP-ALL samples (n=29) to SM treatment ex vivo. We evaluated the efficacy of four different SMs, the monovalent compounds AT406 (Debiopharm Int.) and LCL161 (Novartis) and the bivalent compounds Birinapant (Medivir) and BV6 (Genentech), in inducing cell death in BCP-ALL primograft cells ex vivo. We identified a subgroup of BCP-ALL samples to be highly sensitive to SM-induced cell death (n=8). Further molecular characterization of the samples’ response to SM treatment revealed that TNF signaling is indispensable for SM-mediated cell death as co-incubation with Enbrel, a TNFR2-Fc fusion protein, completely prevented cell death induced by SMs. Moreover, we found that SMs induced different modes of cell death in the different xenografts as determined by inhibitors of caspase or RIP1K activity. Interestingly, in a subset of xenografts caspase inhibition resulted in increased SM-induced cell death indicating that caspase activity prevents SM-induced cell death in some samples. As protein levels of IAPs did not correlate with sensitivity, and can therefore not be used as predictor of SM sensitivity, we compared the basal gene expression profiles of SM-sensitive and SM-resistant samples to identify candidate genes that might account for resistance to SM-treatment. By using this approach, we identified a characteristic gene signature associated with sensitivity to SM-treatment. In summary, only a subset of BCP-ALL samples is sensitive to single agent treatment with SMs. A successful clinical application of this class of drugs requires the in-depth understanding of the underlying resistance mechanisms. The candidate genes associated with sensitivity to SM-induced cell death might help classifying patients into “responder” and “non-responder” to SM-based treatment regimens and may be used as biomarkers before start of therapy.

## ECDO 63 Impairment of mitochondrial ATP production downregulates Wnt signaling

### Magdalena Bachmann^1^, Roberto Costa^1^, Roberta Peruzzo^1^, Giulia Santinon^1^, Mattia Vicario^1^, Giulia Dalla Montà^1^, Andrea Mattarei^1^, Ruben Quintana Cabrera^1^, Enrico Moro^1^, Luca Scorrano^1^, Massimo Zeviani^2^, Mario Zoratti^1,3^, Cristina Paradisi^1^, Francesco Argenton^1^, Marisa Brini^1^, Tito Calì^1^, Sirio Dupont^1^, Ildikò Szabò^1,3^, Luigi Leanza^1^

#### ^*1*^*University of Padova, Italy;*^*2*^*MRC Mitochondrial Biology Unit, Cambridge, UK;*^*2*^*CNR Institute of Neuroscience, Padova, Italy*

Wnt signaling affects fundamental development pathways by regulating cell differentiation and proliferation. Aberrant activation of this pathway promotes the development of several cancers. Wnt/β-catenin signaling is known to influence mitochondrial function, but the possibility that the mitochondrial energetic state affects Wnt signaling has not been explored so far. Here we show that sub-lethal concentrations of different pharmacological compounds acting on mitochondrial fitness downregulate Wnt/β-catenin signaling in HEK293 cells and colon cancer lines. Accordingly, impaired respiratory chain complex III function in human fibroblasts from a GRACILE syndrome patient led to reduced Wnt signaling with respect to healthy cells. The above data was further validated in vivo in zebrafish reporter lines. Other signaling pathways were unaffected, indicating specificity of the mitochondria-Wnt signaling axis. We identified a mechanism whereby a decrease in mitochondrial ATP reduced uptake of calcium to the endoplasmatic reticulum (ER), leading to ER stress and, as a consequence, to inhibited Wnt signaling. In turn, recovery of ATP level restored Wnt activity. These findings reveal an unexpected mechanism related to the control of Wnt pathway activity by mitochondrial ATP and underline the importance of mitochondrial function and energetic state in cellular signaling.

## ECDO 64 A comparative study of apoptosis in peripheral blood leukocytes in patients with Parkinson's disease and healthy donors

### Julia Teterina^1^, Anna Boyko^1^, Olga Shustova^1^, Maria Grechikhina^1^, Ekaterina Doronina^1^, Natalia Troyanova^1^, Elena Kovalenko^1^, Alexander Sapozhnikov^1^

#### ^*1*^*Shemyakin – Ovchinnikov Institute of Bioorganic Chemistry, Moscow, Russia*

Parkinson's disease (PD) is one of the most common neurodegenerative diseases. In recent years, there are growing evidences that the pathogenesis of this disease is connected with regional and peripheral immune processes. Currently, the association of clinical signs of PD with different characteristics of patient immune status is actively being searched. In the framework of this problem we perform an investigation of functional state of immune cells, in particular, level of apoptosis and autophagy (p62 expression) in cultivated in vitro fractions of peripheral blood leukocytes isolated from a group of patients with Parkinson's disease in comparison with a group of healthy donors.

A total of 29 healthy donors and 27 patients with PD participated in the study. Flow cytometric analysis of early stage (Anexin V+/PI-) of spontaneous and induced (by oxidative stress) apoptosis in the samples of peripheral blood mononuclear cells (PBMC) revealed that proportion of apoptotic cells in the PD group was increased in comparison with the values in the healthy group. This was detected for both, the model of spontaneous apoptosis (54.1% and 29.8%, respectively, p=0.005), and the model of induced (50 µM H_2_O_2_, 20 hours) apoptosis (55.2% and 38%, respectively, p=0.01). No significant difference in these models was registered for the samples of peripheral blood granulocytes (PMN). Evaluation of the activity of the process of autophagy in leukocytes was analyzed by detection of intracellular content of the important marker of autophagy – protein p62. Analysis of the data measured by ELISA in the cell samples lysates showed that intracellular p62 level in PBMC was significantly higher in the PD group compared to the healthy group (3.25 ng/ml and 1.14 ng/ml, respectively, p=0.007). No significant difference in this model was registered for the samples of PMN.

The registered differences between patients and healthy donors can be considered as the possible peripheral biomarkers of PD pathogenesis; application of these biomarkers for clinical practice might extend the current approaches to diagnostics and analysis of the course of the PD.

Disclosure: This work was supported by Russian Science Foundation, grant No. 16-15-10404.

## ECDO 65 Pirh2 plays the oncogenic role in p53-negative lung cancer cells H1299

### V. Mamontova^1^, O. Fedorova^1^, O. Shuvalov^1^, A. Petukhov^1^, N. Barlev and A. Daks^1^

#### ^*1*^*Institute of Cytology RAS, St Petersburg, Russia*

TP53 is the one of the major tumor suppressors, which, in response to various forms of stress, promotes cell cycle arrest, autophagy, and apoptosis. The Pirh2 protein - the product of the human *RCHY1* gene - is a RING-domain E3 ligase that performs ubiquitination of the p53 tumor suppressor and targets it to proteasome degradation. In turn, p53 activates the expression of the *RCHY1* gene, forming a negative regulatory feedback loop. Thus, being a suppressor of p53 activity, Pirh2 promotes carcinogenesis. However, it is worth noting that many types of cancers are characterized by the loss of p53 normal function, and the role of the Pirh2 protein in such cancer cells has not been adequately studied.

We investigated effects of Pirh2 on the tumorigenic potential of non-small cell lung cancer cell line, H1299, which lacks p53 expression. As a result, we found that an increase in Pirh2 expression augmented the proliferation rate and migration potential of these cells. Also, Pirh2 increased cellular resistance to the genotoxic drug, doxorubicin. Knockdown of Pirh2 had opposite effect. We demonstrated that Pirh2 suppresses apoptosis in H1299 both under normal conditions and when treated with doxorubicin. Concomitantly, an increase of c-Myc oncogene expression was detected. This result can partially explain the phenomenon of Pirh2-mediated tumorigenicity of lung cancer cells and provides a new potential target for the development of drugs against p53-negative tumors.

Disclosure: This work was funded by RSF No 18-75-10076

## ECDO 66 The knockout of p53-spesific methyltransferase Set7/9 leads to apoptosis increase in A549 lung cancer cells under genotoxic conditions

### A. Daks^1^, V. Mamontova^1^, O. Fedorova^1^, O. Shuvalov^1^, A. Petukhov^1^, N. Barlev^1^

#### ^*1*^*Institute of Cytology RAS, St Petersburg, Russia*

Lysine-specific methyl transferase Set7/9 was first described as a histone H3-specific methyltransferase. Later, Set7/9 was shown to methylate about 30 non-histone proteins, including the p53 tumor suppressor. TP53 is a known regulator of cell cycle, autophagy, and apoptosis involved in cellular response to various forms of stress.

Previous experiments have demonstrated the role of Set7/9 in p53 response and cellular sensitivity to drugs. By means of the CRISPR/Cas9 genome editing system, we created Set7/9 knockout in A549 human non-small lung carcinoma cells. Using this model cell system, we showed that knockout of Set7/9 increased the sensitivity to genotoxic drugs doxorubicin and cisplatin, and induced apoptosis in cells treated with etoposide and doxorubicin.

We also demonstrated that Set7/9 knockout caused a decrease in the formation of gamma H2A.X repair foci. Based on our findings, we assume that Set7/9 methyltransferase is involved in DNA damage response and can be considered as a potential marker for the efficacy of genotoxic chemotherapy against lung cancer.

Disclosure: This work was funded by RSF No 17-75-10198.

## ECDO 67 Orphan Receptor NR4A3 is a novel target of p53

### O. Fedorova^1^, A. Daks^1^, A. Petukhov^1^, O. Shuvalov^1^, N. Barlev^1^

#### ^*1*^*Institute of Cytology RAS, St. Petersburg, Russia*

Tumor protein p53 is a key tumor suppressor that acts as a transcription factor. It regulates the expression of genes whose products induce cell cycle arrest, autophagy, and apoptosis in response to various stress stimuli. Several published reports suggested a link between the expression of p53 and orphan receptors NR4A. Orphan receptors represent a large group of nuclear receptors for which specific ligands are not known. Members of the NR4A family of orphan receptors (NR4A1, NR4A2 and NR4A3) are implicated in regulation of the genes involved in energy balance, metabolism, angiogenesis, thrombosis, proliferation, cell migration, and apoptosis.

We demonstrated by various assays that p53 directly bound the promoter of NR4A3 gene and induced its transcription. We also showed that overexpression of NR4A3 attenuated proliferation of cancer cells and induced their apoptosis by augmenting the expression of pro-apoptotic genes. We also demonstrated potential mechanism of apoptosis caused by NR4A3 via its physical interaction with anti-apoptotic protein Bcl-2, which sequesters the latter into a non-functional complex. Moreover, the bioinformatics analysis demonstrated that high levels of NR4A3 expression correlated with better survival of breast and lung cancer patients. Our results showed that NR4A3 is a novel transcriptional target of p53, which is involved in apoptosis. Thus, we hypothesize that NR4A3 plays tumor suppressive role.

Disclosure: This work was supported by RSF research project #18-75-10076.

## ECDO 68 Cancer-Testis Antigens – Semenogelins 1 and 2 down-regulates migration of human cancer cell models and sensitize them to genotoxic drugs

### Shuvalov O^1^, Kizenko A^1^, Petukhov A^1^, Fedorova O^1^, Daks A^1^, Barlev N^1^

#### ^*1*^*Institute of Cytology RAS, St. Petersburg, Russia*

Semenogelin 1 and 2 (Sg1 and 2, or Sgs) are main proteins of human semen which prevent its coagulation and provide antibacterial defense. Besides reproductive tissues, Sgs are expressed in several tumors including lung, breast, and pancreatic adenocarcinoma. So, they are referred as cancer-testis antigens. However, their biological role in cancer cells is not currently understood.

Firstly, we carried out bioinformatics meta-analysis of cancer patients’ survival rates depending on the expression level of Sgs (Kaplan-Meier plots). We have shown that the high level of Sgs expression can be associated with either increased or decreased survival of patients depending on the type of malignancy.

To test if Sgs would provide different types of neoplasms with oncogenic or tumor suppressive features, we have established human adenocarcinoma cell models (lung, breast, and pancreatic) that stably overexpressed either Sg 1 or Sg2. We have assessed the influence of Sgs expression on the proliferation, migration and viability of cancer cells. By using wound-healing assay, we have shown that the expression of both Sg1 and 2 led to the decreased migration ability of cells.

By using MTT assay and flow cytometry, we have shown that the expression of Sgs confers susceptibility of our cancer cell models to genotoxic drugs (doxorubicin and cisplatin) and induces metabolic alteration and oxidative stress resulting in increased cell death.

Taken together, our data have demonstrated that in our cancer cells models Sgs displays tumor suppressive functions.

Disclosure: This work was supported by RSF grant #17-75-10205.

## ECDO 69 Necroptosis caused by either TNFR1 or Fas activation shares common signal transduction pathway involving lysosomes and mitochondria

### Yashin D.V.^1^, Sharapova T.N.^1^, Romanova E.A.^1^, Sashchenko L.P.^1^

#### ^*1*^*Institute of Developmental Biology RAS, Moscow, Russia*

Recently we have discovered that Tag7-Hsp70 complex interacts with TNFR1 receptor inducing death of tumor cells via apoptosis and necroptosis. Freshly we have also found that Tag7 protein activate cytotoxic lymphocytes that kill HLA-negative tumor cells via FasL-Fas interaction, inducing both apoptosis and necroptosis. We have shown that transduction of necroptotic signal via TNFR1 and Fas has many common points. Necroptosis begins with RIPK1 activation and necrosome formation. Subsequent activation of MLKL results in the increase of Ca2+ level in the cell and activation of Ca2+-dependent enzymes causing lysosomal membrane permeabilization and the release of cathepsins to the cytosol. STAT3 translocation to the mitochondria and binding to a component of the respiratory chain complex I causes ROS accumulation. The common signal transduction pathway in tumor cells induced by two distinct death receptors is very interesting observation. It is known that tumor cells often evade apoptosis using various mechanism to escape immune system control. Alternative activation of necroptosis pathway is a backup pathway to ensure target cell death. Knowledge about necroptosis signal transduction pathway will help to develop medicals that will help to keep immunoescaping tumor cells under immune system control.

Disclosure: The work is supported by RSF 15-14-00031-P.

## ECDO 70 Functional characterization of two novel serine protease inhibitors able to block CD44-triggered necroptosis in GM-CSF-primed neutrophils

### Xiaoliang Wang^1^, Martina Gobec^2^, Shida Yousefi^1^, Irena Mlinarič-Raščan^2^, Hans-Uwe Simon^1^

#### ^*1*^*University of Bern, CH-3010 Bern, Switzerland;*^*2*^*University of Ljubljana, Aškerčeva 7, SI-1000 Ljubljana, Slovenia*

The most common form of neutrophil death is apoptosis under both physiological and inflammatory conditions. However, neutrophil necroptosis has also been described, a process which depends on receptor-interacting protein kinase-3 (RIPK3) and mixed lineage kinase-like (MLKL) activities. The mode of cell death has consequences on the surrounding environment and the subsequent inflammatory responses. This is especially relevant for neutrophils, which contain granules filled with reactive chemicals and enzymes. Clearly, the death of a necrotic neutrophil may induce inflammatory responses by the immediate release of danger-associated molecular patterns (DAMPs), but also by causing tissue damage. In this report, we investigated the effect of newly synthesized serine protease inhibitors on neutrophil death in vitro. Two inhibitors have been identified that are able to inhibit CD44-induced necroptosis in GM-CSF-primed neutrophils. In contrast, FAS receptor-mediated apoptosis was not blocked. Interestingly, both of these two serine protease inhibitors can inhibit PI3K and NADPH oxidase activities in CD44-induced necroptosis, but only one of them could block MLKL phosphorylation. Therefore, some neutrophil serine proteases appear to initiate production of CD44-induced reactive oxygen species and subsequent neutrophil necroptosis. In future work, we plan to focus on identifying these serine proteases, because they could be potential targets for therapeutic modulation of neutrophil-associated disorders in infectious, inflammatory and autoimmune diseases.

## ECDO 71 VCP inhibition kills cancer cells via proteostatic disruption of the ER and mitochondria

### Dong Min Lee^1^, Min Ji Seo^1^, In Young Kim^1^, Seok Soon Park^2^, Kyeong Sook Choi^1^

#### ^*1*^*Ajou university graduate school of medicine, Suwon, Korea;*^*2*^*University of Ulsan College of Medicine, Seoul 05505, Korea*

Valosin-containing protein (VCP), an ATPase whose function is critically linked to the maintenance of proteostasis, has emerged as a novel therapeutic target in cancer cells. However, it is not clearly understood how VCP inhibition demonstrates anti-cancer effects. In the present study, we investigated the underlying mechanisms by which VCP inhibition induces the stress responses and death in various cancer cells.

The effects of VCP inhibition by its chemical inhibitors, siRNAs, and the dominant negative mutant of VCP on the viabilities of various cancer cells were examined employing IncuCyte or Live & Dead kit. In vivo anti-tumor effect of CB-5083, a VCP inhibitor, was investigated in MDA-MB 435S cell xenograft model. The morphological changes of cancer cells following VCP inhibition were observed by the confocal and electron microscopy. The expressions of the proteins of interest were investigated by immunoblotting and immunocytochemistry.

We found that paraptosis, a cell death accompanied by the dilation of the endoplasmic reticulum (ER) and mitochondria, critically contributes to the anti-cancer effect of VCP inhibition. The failure in the ER-associated degradation (ERAD) and mitochondria-associated degradation (MAD) by VCP impairment is responsible for the disruption of proteostasis, subsequent dilation of these organelles, and a final cancer cell death. In addition, we found that ATF4 is critically involved in the cellular stress response and cell

Our results suggest that many cancer cells may critically depend on VCP for their proteostatic maintenance and survival. Thus, VCP inhibition may provide a therapeutic strategy in various malignant cancer cells via induction of paraptosis, a cell death mode associated with the proteotoxic stress to the ER and mitochondria.

## ECDO 72 The C-terminus of p63 isoforms regulates distinct functions in different organs

### Eleonora Candi^1^

#### ^*1*^*University of Rome “Tor Vergata”, Rome, Italy*

The transcription factor p63, member of the p53 family, mediates different cellular responses and it is involved in different processes. Due to the presence of two independent promoters, two classes of p63 proteins are expressed that differ at the (N)-terminus: TAp63 and ΔNp63. TAp63 is highly expressed in a dimeric inactive conformation in female germ cells during meiotic arrest. The ΔNp63 proteins are found primarily expressed in the basal regenerative layers of the epidermis and other stratified epithelia, they are important to mantain high proliferative potential of epidermal stem cells. The 3’-end of p63 mRNA undergoes to tissue specific multiple processing, generating a, b and g isoforms. An attractive hypothesis is that the different isoforms fulfills different functions in various cellular level and during development. To address this in vivo, a p63a specific knock-out mouse was generated in which the endogenous p63a isoform is targeted and replaced with p63b isoform. Interestingly, the heterozygous female mice, bearing one p63a and one p63b allele (HETp63) are sterile. This makes it impossible to generate a full p63a knock out, but opens up the question why the mice are sterile. We evaluated several organs to study simultaneous expression of both p63a and p63b contribution in development. First, in new born ovaries we observed that the number of primary oocytes was strongly reduced. In the ovary, the active TAp63 C-terminus induces apoptosis via enhanced expression of the pro-apoptotic Bbc3 (Puma) and Pmaip1 (Noxa), independently of the DNA-damage driven phosphorylation and activation signals. On the other hands, no abnormalities were detected in development of organs, such as thymus and skin, expressing mainly DNp63a, indicating that in these cases DNp63b isoform does not interfere and synergistically acts with DNp63a. These results demonstrated that in vivo p63 C-terminus domains, including TID and SAM domain, have different functional role in the TAp63 or DNp63 isoforms.

## ECDO 73 Implication of immune system in Vaccinia virus‐induced cell death

### Olga Koval^1,2^, Galina Kochneva^3^, Olga Troitskaya^1^, Anastasiya Tkachenko^1^, Anna Nushtaeva^1^, Elena Kuligina^1^, Vladimir Richter^1^

#### ^*1*^*Institute of Chemical Biology and Fundamental Medicine SB RAS, Novosibirsk, Russia*^*2*^*Novosibirsk State University, Novosibirsk, Russia;*^*3*^*State Research Center of Virology and Biotechnology “Vector” Rospotrebnadzor, Koltsovo, Russia*

Immunotherapeutic approaches become a new hope for cancer treatment. Among them oncolytic viruses demonstrate clinical success combining oncolytic effects and the inflammatory response against variety of solid tumors. Genetically modified *Vaccinia virus* (VV) can be particularly useful for tumors with drug resistance syndrome. However, mechanisms underlying *Vaccinia virus* immunity demand extensive research for further improving therapeutic efficacy of this agent. We have engineered double recombinant *Vaccinia virus* (VV-GMCSF-Lact) coding human granulocyte-macrophage colony-stimulating factor (GM-CSF) and apoptosis-inducing protein lactaptin. Lactaptin was discovered as a molecule specifically inducing death of various cancer cells in vitro and in vivo. VV-GMCSF-Lact efficiently induces death of infected tumor cells by activation of critical apoptosis markers. The analysis of antitumor activity against advanced MDA-MB-231 tumor in mice revealed that VV-GMCSF-Lact delayed the tumor growth up to 94% after the course of intratumoral injections. Virus replication and immune cells infiltration of distant tumor nodes were visualized immunohistochemically. We detected VV-GMCSF-Lact-increased phagocytic activity of peritoneal macrophages that can also improve its antitumor effect. Treatment of tumor cells with VV-GMCSF-Lact and tyrphostin (an EGF receptor inhibitor) decreased the efficiency of oncolysis that has proved the importance of EGF signaling pathway in VV-dependent cell death. Experiments revealing the role of EGF signaling in VV-GMCSF-Lact-mediated cell death is underway.

Investigating immunogenic cell death (ICD) markers in CT26 and Mx7 mouse cancer cells infected with VV-GMCSF-Lact we demonstrated an efficient VV-mediated calreticulin and HSP70 protein externalization, and the release of cellular high-mobility group box-1 (HMGB1) protein and ATP. However, comparing in vivo vaccination effects of VV-treated cells and lactaptin-treated cells, we shown tumor-protective properties only for lactaptin-treated cells. This result could be partly explained by the fact that VV particles are more immunogenic than destroyed tumor cells.

Disclosure: Supported by the VolkswagenStiftung Grant #90315 and by the Russian Science Foundation Grant RSF 16–14-10284.

## ECDO 74 DNA methyltransferase 3A regulates autophagy long-term memory

### Patricia González-Rodríguez^1^, Jens Füllgrabe^1^, Mathilde Cheray^1^, Virginia Cunha^1^, Kristian Dreij^1^, Bertrand Joseph^1^

#### ^*1*^*Karolinska Institutet, Stockholm, Sweden*

Autophagy is a conserved catabolic pathway that targets cytoplasmic components to autophagosome-dependent lysosomal degradation. Autophagy induction is highly regulated by a core set of Atg (Autophagy-related) proteins that are involved in different steps of the process. Multiple forms of cellular stress as starvation, hypoxia, oxidative stress, protein aggregates, pathogens, etc. stimulate this pathway. Transcriptional control is critical and needs to be finely regulated. In our study, we report that short induction of autophagy is linked in both in vitro and in vivo to a persistent downregulation of MAP1LC3 isoforms expression, key components of the autophagy core machinery, through de novo DNA methyl-transferase DNMT3A-dependent locus-specific DNA methylation. This acquired epigenetic memory of autophagy is sustained in offspring generations and might influence the way cells and potentially whole organisms are able to adapt by make them remember the initial stimuli and less responsive to following treatments.

## ECDO 75 Proteomics reveals the role of autophagy in cornification of keratinocytes

### Leopold Eckhart^1^, Karin Jaeger^1^, Supawadee Sukseree^1^, Shaomin Zhong^1^, Brett Phinney^2^, Veronika Mlitz^1^, Florian Gruber^1,3^, Robert Rice^4^, Erwin Tschachler^1^

#### ^*1*^*Medical University of Vienna, Vienna, Austria;*^*2*^*University of California, Davis, CA, USA;*^*3*^*Christian Doppler Laboratory on Biotechnology of Skin Aging, Vienna, Austria;*^*4*^*University of California, Davis, CA, USA*

Epithelial cells of the skin undergo special modes of programmed cell death during terminal differentiation. Targeted gene inactivation studies in mice have revealed essential roles of DNase1L2 and DNase2 in nuclear DNA degradation and less-clearly defined contributions of autophagy factors in other degradation processes within epithelial cells that undergo either cornification in the epidermis or holocrine secretion in sebaceous glands. Here, we determined the role of autophagy in the cornification of nails, which represent a unique model system for the analysis of the cross-linked and stabilized proteome of dead cells. Autophagy was suppressed by deletion of the essential autophagy factor Atg7 in keratin K14-expressing cells (*Atg7*^*f/f*^*K14-Cre*). The abrogation of autophagy did not cause significant changes in gene transcription but resulted in significant increases in the abundance of a broad range of enzymes and other non-structural proteins. In particular, the amounts of the subunits of the proteasome and of the TRiC/CCT chaperonin were strongly elevated in mutant mouse nails, indicating an essential role of autophagy in removing these large protein complexes during normal cornification. By contrast, the abundance of keratins and keratin-associated proteins was decreased in nails of *Atg7*^*f/f*^*K14-Cre* mice. The results of this study suggest that autophagy degrades non-cytoskeletal proteins and leaves the cytoskeleton intact when keratinocytes undergo cornification to form mechanically resistant skin appendages.

## ECDO 76 Angiogenic, apoptotic and autophagic profiling of chronic myeloid leukaemia patients’ platelets ex vivo before and after treatment with imatinib

### L Repsold^1^, R Pool^1^, G Tintinger^1^, M Karodia^2^, AM Joubert^1^

#### ^*1*^*University of Pretoria, Pretoria, Gauteng, South Africa*^*2*^*Sefako Makgatho Health Sciences University, Ga-Rankuwa, Pretoria, Gauteng, South Africa*

Introduction: Chronic myeloid leukaemia (CML) is characterised by a long sub‐clinical period wherein there is a slow build‐up of abnormal white blood cells. In addition, platelets play an important role in cancer and tumour development, in particular their involvement in angiogenesis in tumours. Therapies directed at specifically targeting angiogenesis is a recognized method of antitumour therapy, however, it is not well researched in haematological malignancies.

Aim: In this ex vivo study the mechanism of CML progression, treatment and the signalling involved in the angiogenic, apoptotic and autophagic profiles and protein levels of these markers in CML patients’ platelets before and after treatment with Imatinib were investigated.

Materials and Methods: Blood was collected from five CML patients according to staging and exclusion‐and inclusion criteria, as well as 30 control participants. Collection of blood from CML patients occurred at diagnosis and after 6 months of treatment with Imatinib (400 mg per os/day). The angiogenic‐, apoptotic‐ and autophagic profile of CML patients was determined ex vivo on platelets by means of flow cytometry, electron microscopy, and western blot analysis.

Results: Preliminary results indicated an initial high platelet count in CML patients at time of diagnosis. After 6 months of treatment with Imatinib, platelet levels lowered to normal ranges. These results correlated with Annexin‐ V analysis which indicated a slight increase in apoptosis in CML patients’ platelets. At the time of diagnosis, CML patients presented with platelet dysfunction that may be as a result of the pathogenesis of CML. After 6 months of treatment with Imatinib these levels normalised, either as a result of treatment with Imatinib or as a result of increased apoptosis resulting from treatment with Imatinib.

Discussion and Conclusion: The importance of angiogenesis‐ targeted therapies in CML has recently become evident as the occurrence of tyrosine kinase inhibitor resistance and, specifically Imatinib resistance, increases. In CML, this resistance has recently become linked to bone marrow (BM) angiogenesis which aids in the growth and survival of leukaemia cells. This study will enable researchers to focus on affected cellular mechanisms and the identification of targets for therapeutic intervention.

## ECDO 77 Systemic network analysis identifies XIAP as potential target in TRAIL resistant BRAF mutant melanoma

### Greta Del Mistro^1^, Philippe Lucarelli^2^, Ines Müller^1^, Sébastien De Landtsheer^2^, Anna Zinoveva^1^, Meike Hutt^3^, Martin Siegemund^3^, Roland E. Kontermann^3^, Stefan Beissert^1^, Thomas Sauter^2^, Dagmar Kulms^1,^

#### ^*1*^*TU-Dresden, Dresden, 01307, Germany;*^*2*^*University of Luxembourg, Belvaux, 4367, Luxembourg;*^*3*^*University of Stuttgart, Stuttgart, 70569, Germany*

Metastatic melanoma remains a life-threatening disease because most tumors develop resistance to targeted kinase inhibitors thereby regaining tumorigenic capacity. We show the novel second generation hexavalent TRAIL receptor-targeted agonist IZI1551 to induce pronounced apoptotic cell death in *mut*BRAF melanoma cells. Aiming to identify molecular changes that may confer IZI1551 resistance we combined Dynamic Bayesian Network modelling with a novel regularization strategy resulting in sparse and context sensitive networks and show the performance of this strategy in the detection of cell line specific deregulations of a signalling network. Comparing IZI1551-sensitive to IZI1551-resistant melanoma cells the model accurately and correctly predicted activation of NFκB in concert with upregulation of the anti-apoptotic protein XIAP as the key mediator of IZI1551 resistance. Thus, the incorporation of multiple regularization functions in logical network optimization may provide a promising avenue to assess the effects of drug combinations and to identify responders to selected combination therapies.

## ECDO 78 Single-molecule imaging of the plasma membrane TNFR1 equilibrium reveals novel roles for ligand-independent TNFR1 clustering in cell fate control

### Sjoerd J. L. van Wijk ^1^, Christos Karathanasis ^1^, Sonja Smith ^1^, Sebastian Malkusch ^1^, Simone Fulda ^1^, Ivan Dikic ^1^, Mike Heilemann ^1^

#### ^*1*^*Goethe University, Frankfurt am Main, Germany*,

Ligand-induced TNFR1 reorganization and activation pleiotropically controls cell proliferation, programmed cell death and survival. Despite the relevance of TNFR1 clustering for cell fate signaling, the spatial distribution of TNFR1 in the plasma membrane and in particular the role of receptor dimerization has remained controversially debated. At present, TNFR1 models range from ligand-independent receptor pre-dimerization to ligand-induced receptor oligomerization. Clustering-deficient mutations in TNFR1 and other death receptors are causing autoimmune syndromes, rheumatoid arthritis and dysregulation of cell death and death receptor blockage has substantial potential for therapeutic intervention.

Here, we explore the molecular and cellular determinants that control TNFR1 clustering using quantitative single-molecule localization microscopy (*d*STORM/PALM) at physiological cell surface expression levels. In the absence of ligand, TNFR1 occurred as monomers/dimers and TNFR1 clusters into trimers and higher oligomers upon TNF binding. Ligand-induced TNFR1 clustering was prevented with anti-TNFR1 antibodies that also inhibit downstream NF-ĸB signalling. Furthermore, prominent ligand-independent TNFR1 dimerization through the pre-ligand assembly domain (PLAD) could be imaged at the plasma membrane as well. PLAD TNFR1 dimers decreased upon TNF binding, while TNFR tri- and oligomers increased, suggesting pre-complex formation and dynamic TNFR1 equilibriums at the membrane. Intriguingly, chemical inhibition of PLAD interactions and TNFR1 PLAD mutations disrupt PLAD dimerization, TNFR1 oligomerization and NF-ĸB signalling, underscoring the significance of ligand-independent TNFR1 dimerization in cell fate control in healthy and tumour cells.

In conclusion, our findings confirm an intricate network of homotypic TNFR1 domain associations, both intracellular and extracellular through the PLAD and ligand-receptor interactions that shape cell fate.

## ECDO 79 Wild-type p53 phosphatase regulates the cell death pathway in neutrophils

### B. Uyanik^1,2^, C. Garrido^1,2,3^, O.N. Demidov^1,2,4^

#### ^*1*^*INSERM UMR 1231, Lipids Nutrition Cancer, Dijon, France;*^*2*^*University of Burgundy, Dijon, France;*^*3*^*Anticancer Center Georges François Leclerc, Dijon, France;*^*4*^*Institute of Cytology, RAS, St. Petersburg, Russia*

Neutrophils are one of the main effectors of the immune system. They have a fundamental role in innate immunity, host defense mechanisms and are the first cells recruited to the site of inflammation.

Neutrophils are continuously produced in the bone marrow and represent the most abundant fraction of white blood cells in human. Neutrophils are short-lived cells, which survive in circulation only for 1-2 days. Interestingly, their lifespan is influenced by various signals during the inflammatory response, which can delay or induce their death. These multiple signals have been associated with different mechanisms of cell death and are intimately related to physiological functions of neutrophils.

Several studies have shown that prolongation of neutrophil lifespan is critical for effective host defense. The primary signaling pathways involved in neutrophil survival and death such as p38, NFKB or Bcl2 family are known to be preferential targets of Wip1 phosphatase.

Wild-type p53 induced phosphatase, Wip1, can directly dephosphorylate and inhibit the activities of MAPK or NFKB signaling, thereby supporting neutrophil apoptosis and, in doing so, modifying neutrophil properties and regulating the inflammatory response.

In this context, we determined that Wip1 can regulate neutrophils apoptosis and necroptosis through modulation of caspase 8, caspase 9 and RIPK1 signalization. We also discovered that the inhibition of Wip1 activity could extend neutrophil survival and control critical functions of neutrophils associated to death such as NETosis.

In conclusion, we have confirmed the crucial role of Wip1 in neutrophil survival pathways and regulation of their lifespan. The targeting of Wip1 activity in neutrophils could be a new therapeutic strategy in the treatment of several diseases that trigger the inflammatory response.

## ECDO 80 GSDMD-deficient mice are protected from TNF-induced SIRS

### Wulf Tonnus^1^, Andreas Linkermann^1^

#### ^*1*^*University Hospital Carl Gustav Carus, Technical University Dresden, Germany*

The role of necroptosis has been established in TNF-induced severe inflammatory response syndrome (SIRS), and pyroptosis-deficient gasdermin D (GSDMD)-ko mice have been reported to be protected against LPS-induced death. However, to the best of our knowledge, the role of gasdermins in TNF-induced shock has not been investigated. Here, we report that GSDMD-ko mice are strikingly protected from TNF-induced death. In direct comparison to MLKL-ko mice that exhibited a rate of more than 35% deaths 72 hours after TNF injection, all GSDMD-ko mice (n=20) survived the observation period of 7 days and were almost unaffected regarding movements and body temperature. We conclude that GSDMD mediates the lethality of the TNF shock model. In addition to the sepsis models, we performed cisplatin-induced acute kidney injury and kidney ischemia-reperfusion injury (IRI) in GSDMD-ko mice and will present the results of these experiments on the ECDO meeting for the first time. Our data indicate that functionally, TNF signaling is upstream of GSDMD-mediated pyroptosis.

## ECDO 81 Selective caspase-2 inhibition and synapse protection with a new irreversible pentapeptide derivative

### Elodie Bosc^1^, Julie Anastasie^1^, Feryel Soulami^1^, Ségolène Pretat^1,2^, Gullen Lacin^1,2^, Eric Duplus^1^, Philippe Tixador^1^, Hugo Cochet^1,2^, Bernard Brugg^1^, Chahrazade El Amri^1^, and Etienne Jacotot^1^

#### ^*1*^*Sorbonne Université - CNRS UMR8256 - INSERM U1164. Institut de Biologie Paris-Seine. 7 quai St Bernard 75005 Paris, France;*^*2*^*MicroBrain Biotech. ESPCI - Institut Pierre-Gilles de Gennes., 6 rue Jean Calvin 75005 Paris; France*

Caspase-2 (Casp2) has emerged as a unique Caspase with potential roles in maintaining genomic stability, metabolism, chronic and acute neurodegeneration, and aging. We have for instance established that Casp2 is a key initiator of neuronal cell death in neonatal brain damage^1^, and recent findings show that Casp2 is a potential therapeutic target in human diseases, including optic nerve injury^2^, and Alzheimer disease^3,4^. Consequently, there is a need to design Casp2 selective inhibitors (or substrates) to precisely define its apoptotic and non-apoptotic roles and to interfere pharmacologically with disease-related pathways. Most Caspase inhibitors have consisted in active site-directed peptide-based inhibitors. However, the vast majority of the substrate-derived Caspase inhibitors lack sufficient specificity to inhibit individual Caspase. Furthermore, Casp2 and Casp3 share similar features regarding their active sites and the well-defined pentapeptide-based inhibitors of Caspase-2 (i.e. VDVAD) are also efficient inhibitors of Casp3. Previous works^5^, based on rational design study indicate that a bulky residue at P2 position within the non-selective Ac-VDVAD-CHO peptide result in peptides which present potent inhibition effect on Casp2 and are less recognized by Casp3. We have extended this approach and defined a new irreversible pentapeptide derivative, named LJ-2, which combine an amino-terminal quinolyl-carbonyl and a C-terminal difluorophenoxy-methyl-ketone warhead with the Casp2-preferred pentapeptide backbone VDVAD where the Ala in position P2 is replaced by a substituted isoquinoline. Kinetics using recombinant human Casp2 and Casp3 show that, when compared to the VDVAD-based group II caspase inhibitor Δ2Me-TRP601,^6^ LJ-2 has very strong off-rate (k3/Ki) and increased selectivity toward Casp2.^7^ As Casp2 is required for β-amyloid-induced synapse loss in cellular and animal models^3^, we have established microfluidic neuronal culture systems where hippocampal neurons or compartmentalized cortico-hippocampal neuronal networks are subjected to β-amyloid [Aβ1-42] oligomers treatments to trigger synapse-loss. In these microsystems, Cre recombinase-mediated Casp2 knock-out blocks Aβ-induced synaptotoxicity in primary *casp2* ^*flox/flox*^ hippocampal neurons^8^, and submicromolar concentrations of LJ2 prevent synapse loss in wild-type neurons treated with β-amyloid [Aβ1-42] oligomers. Hence, LJ-2 is the first potent and selective irreversible Casp2 inhibitor.

## ECDO 82 Applying imaging flow cytometry for the characterization and quantification of NETosis and cell death in vitro and in vivo

### Patrick M. Lelliott^1^, Masatoshi Momota^1,2^, Michelle S. J. Lee^1^, Etsushi Kuroda^1,2^, Norifumi Iijima^1,2^, Ken J. Ishii^1,2^, Cevayir Coban^1^

#### ^*1*^*Immunology Frontier Research Center (IFReC), Osaka University, Osaka, Japan;*^*2*^*National Institutes of Biomedical Innovation, Health and Nutrition (NIBIOHN), Osaka, Japan*

The unique form of cell death, termed NETosis, involves the release of extracellular DNA peppered with antimicrobial proteins and histones which can immobilize and kill pathogens. Neutrophil extracellular traps (NETs) are being increasingly recognized to be playing a key role in a wide range of diseases and conditions, with both beneficial and detrimental outcomes.

A major issue plaguing research into NETosis is a lack of reliable, non-biased, and high-throughput techniques for the quantification of NETs both in vitro and in vivo. Studies have relied on quantification methods such as the measurement of DNA release or the presence of histone citrullination, however, this is increasingly being criticized as insufficient to discriminate NET formation from other types of cell death. This is particularly a problem in vivo, for which multiple factors could be influencing DNA levels and other markers of NET release.

We have developed a novel technique to identify and characterize NETosis using imaging flow cytometry, thereby allowing for the rapid and accurate quantification of this phenomenon both in vitro and in vivo. This technique is able to discriminate not only NETs, but also cells undergoing nuclear decondensation (a precursor step before NET formation), DNA fragments, and other types of cell death which occur without DNA release. We describe the application of imaging flow cytometry to measure PMA induced NETs in vitro, and use a malaria infection model to demonstrate the ability of this technique to measure NETs in whole blood samples in vivo. We outline an image analysis algorithm allowing the automated quantification of these cell types, thereby removing any potential operator bias in this approach.

We expect this technique will provide a valuable tool to better understand the process of NET formation and its importance in disease.

## ECDO 83 Ferroptosis and its detrimental role in multiple sclerosis

### Emily Van San^1,2^, Behrouz Hassannia^1,2^, Nele Goossens^2^, Sze Men Choi^1,2^, Conor McGuire^1,2^, Sofie Voet^1,2^, Geert Van Loo^1,2^, Peter Vandenabeele^1,2^, Tom Vanden Berghe^1,2^

#### ^*1*^*VIB-UGent Center for inflammation Research, Ghent, Belgium;*^*2*^*Ghent University, Ghent, Belgium*

Multiple sclerosis (MS) is a chronic neuronal disorder characterized by central nervous (CNS) inflammation, demyelination and, in cases of chronic disease, astroglial scarring. The formation of demyelinated lesions leads to axonal injury and neurological deficits. Typically, MS, like many other autoimmune inflammatory diseases, is at least partially driven by a mutual auto-amplifying mechanism of inflammation and cell death. However, the exact contribution of cell death and its therapeutic potential is still unclear. Basically, the detrimental cell death occurs mainly in oligodendrocytes and neurons, but it is still largely unknown how these cells die. Iron homeostasis at the cellular level is important to maintain proper cellular function, and its dysregulation can contribute to neurodegenerative diseases. Interestingly, oligodendrocytes contain high levels of iron in the brain, while iron is also accumulating in neurons during aging. Essentially, this increases the vulnerability of these cells to iron-dependent cell death called ferroptosis. In ferroptosis, the generation of redox-active iron promotes the formation of phospholipid peroxyl radicals through Fenton-type reactions and activation of lipoxygenases, which drives the process of lipid peroxidation and ultimately cell death. Strikingly, it is shown that iron deposition correlates with oxidized phospholipids and demyelination in multiple sclerosis lesions. Ferroptotic cell death is genetically counteracted by the oxidized phospholipid repair enzyme Glutathione Peroxidase 4 (GPX4), and pharmacologically by iron chelators, natural lipophilic free radical traps such as vitamin E and synthetic lipophilic free radical traps such as ferrostatin-1 (Fer-1) and liproxstatin-1 (Lip-1). In this study, we investigate the role of ferroptosis in MS using different experimental models of MS and following both genetic and pharmacological approaches. Interestingly, a high vitamin E diet protects mice completely from cuprizone-induced demyelination while no effect was observed in experimental autoimmune encephalomyelitis (EAE), which suggests a differential contribution of ferroptosis in both experimental models of MS. Nevertheless, GPX4 overexpression delayed the EAE-induced disease onset. We developed a novel Fer-analogue with improved solubility and in vivo stability that crosses the blood-brain barrier. Preliminary data suggest that our Fer-lead can reduce the EAE-induced disease score. To explore the therapeutic potential of our Fer-lead, we have set up an experimental relapsing-remitting model of multiple sclerosis in Biozzi ABH mice. Future research will focus on the added value of our novel Fer-lead along current autoimmune-suppressing therapies to improve disease severity in different experimental models of MS.

## ECDO 84 Investigating the Stability and Localisation of the E3 Ubiquitin Ligase Pellino2

### Callum McGrenaghan^1^, José Bengoechea ^1^, Paul N. Moynagh ^1,2^, Adrien Kissenpfennig^1^

#### ^*1*^*Queen’s University Belfast, Belfast, UK;*^*2*^*National University of Ireland Maynooth, Maynooth, Co. Kildare, Ireland*

The Pellino family of E3 ubiquitin ligases are fast emerging as key regulators of cell death signalling pathways. First identified as interaction partners of the IL-1 receptor associated kinases (IRAKS), the Pellino family consists of 3 members: Pellino1, Pellino2 and Pellino3, the latter exists in two alternately spliced forms pellino3a and 3b. The Pellino family have been shown to interact with the receptor interacting protein (RIP) kinases, known key modulators of cell death signalling. Pellino3 has been shown to regulate the pro-apoptotic response in TNF receptor signalling. Pellino1 has been shown to be a dual modulator of apoptosis and necroptosis in the same pathway. Pellino2 is the least studied of the family and has recently been shown to be critical in the activation of the NLRP3 inflammasome, facilitating maturation and release of IL-1β and IL-18, promoting pyroptosis in macrophages. Little is known of the localisation and stability of Pellino2. To address this a Pellino2-eGFP fusion protein was generated.

Classical molecular cloning methods were used to generate the human Pellino2-eGFP fusion protein. Pellino2-eGFP was transfected into 293Ts and its expression was validated by fluorescence microscopy and western blotting. To validate that the fusion protein remained functional it was co-expressed in 293Ts along with a known interaction partner IRAK1. Localisation of Pellino2-eGFP was determined by confocal microscopy. Protein stability was assessed in 293Ts transfected with pellino2-eGFP using the proteasome inhibitor MG262. Lentiviral transduction was used to overexpress a myc-tagged murine Pellino2 in immortalised bone marrow derived macrophages. Stability of Pellino2-myc was assessed in response to treatment with LPS.

Pellino2-eGFP was successfully validated by western blot and fluorescence microscopy. Pellino2-eGFP retained its E3 ubiquitin ligase activity as co-transfection with IRAK1 resulted in the polyubiquitination of IRAK1. Pellino2-eGFP was shown to localise within the cytoplasm and nucleus by confocal microscopy. Pellino2-eGFP accumulated following addition of MG262 when compared to both an untreated control and an eGFP empty vector control. Murine Pellino2-myc levels decrease at the 3 and 6 hour time point post-LPS stimulation. These results show that human Pellino2 is targeted for proteasomal degradation. Murine Pellino2 degrades to below the levels of the untreated control 6 hours after stimulation with LPS, suggesting its degradation is required for the resolution of inflammation.

## ECDO 85 Selective small molecule stabilisers/activators of p53(Y220C) mutant

### Regina Sayarova^1^, Raniya Nazyrova^1^, Rimma Mingaleeva^1^, Olga Kartseva^1^, Irina Glagoleva^1^, Natalya Alexandrova^1^, Azzam Hamad^1^, Razan Subani^1^, Vitaly Chasov^1^, Rafil Khairullin^1^, Regina Miftakhova^1^, Matthias Baud^2^, Albert Rizvanov^1^, Emil Bulatov^1^

#### ^*1*^*Kazan Federal University, Russia;*^*2*^*University of Southampton, UK*

In about 50% of tumor cases inactivation of p53, a key oncosuppressor and transcription factor, is caused by mutations that primarily affect DNA-binding domain. Oncogenic missense mutation Y220C is the ninth most common for p53 and is annually observed in about 100,000 new diagnosed cancer cases worldwide. Presence of this mutation disturbs tertiary structure of the p53 DNA-binding domain that further leads to destabilization of the whole protein, its partial denaturation and loss of transcriptional activity. Selective small molecule modulators of p53(Y220C) mutant can be used to structurally stabilize the protein and restore its impaired transcriptional functions. Aminobenzothiazole MB725 and its derivatives represent a highly promising scaffold for development of potent stabilisers/activators of p53(Y220C) mutant.

In this study we employ a wide spectrum of modern interdisciplinary methods and approaches to explore how small molecules could effectively restore biological activity of p53(Y220C) mutant. This includes organic synthesis, structural biology and a range of molecular and cell biology techniques. Mutant p53(Y220C) and p53-/- MCF7 cell lines are being generated using CRISPR/Cas9 gene editing technology. Cellular effect of the compounds is investigated using following methods – assessment of cytotoxicity by MTS test; monitoring of cell proliferation and viability using xCELLigence real time cell analysis; analysis of p53-dependent gene expression by quantitative real-time reverse transcription PCR; quantitative analysis of alterations in intracellular protein levels by immunoblotting; cytofluorometric analysis of cell cycle and programmed cell death. Evaluation of mutant p53(Y220C) stabilization against intracellular proteasomal degradation to be performed using fluorescent reporter system. In addition, we studied interaction of MB725 and its analog with recombinantly expressed and chromatographically purified p53 and p53(Y220C). For that we used two biophysical methods – surface plasmon resonance to measure interaction Kd and differential scanning fluorimetry to estimate protein thermal stability in presence of the compounds. The results can be used to develop personalised therapeutics targeting not only Y220C, but also other p53 mutants.

Disclosure: The study was supported by Grant of the President of Russian Federation MK-4253.2018.4 to E.B. and RFBR 18-34-00702 mol_a Grant to R.S.

## ECDO 86 3D-cell model system for the early diagnosis of cell sensitivity to antitumor agents by evaluating of mitochondrial activity

### Elena Petersen^1^, Ekaterina Skorova^1^, Eugenia Shabalina^1^

#### ^*1*^*Moscow Institute of Physics and Technology, Dolgoprudny, Russia*

The problem of tumor resistance to the therapy remains very relevant at the moment due to the fact that therapeutic methods that exist at the moment have been developed without taking into account new information on more complex multicomponent processes of formation of malignant neoplasms. The results obtained in such systems do not always fully correspond to the data obtained in clinical trials.

In this study, we present new data from dynamic observations assessing the sensitivity of various cancer lines to the damaging effects of DMSO and doxorubicin, using a complementary set of biosensors. Signal levels of mitotracker orange and fluorescein diacetate referred to the functional state of both cellular and mitochondrial membranes. As a result we obtained two simultaneous kinetic curves of the reactions in the dynamic detection system of the two sensors that allowed us not only to estimate the final state of the cells, but also to study the individual stages of signal transmission of apoptosis from the periphery of the cell. For instance this system allowed us to show that cellular reaction on 5 and 10% DMSO differs not only in speed of changes in dye intensity (decrement angles -0,10147 and -0,0395 resp. for fluorescein; -0,00475 and +0,0083 resp. for mitotracker orange) but also inform of reaction curve (see – 0, 00475 vs. +0, 0083), that in turn suggests that processes underlying different kinetics of dye intencities should have different mechanisms.

Disclosure: This research was supported by RSF grant (№ 18-15-00391).

## ECDO 87 PERK and IRE1α axis of the unfolded protein response contribute to immunogenic cancer cell death (ICD)

### Nicole Rufo^1^, Abhishek D. Garg^1^, Francesca Finotello^2^, Zlatko Trajanoski^2^, Lasse Sinkkonen^3^. Thomas Sauter^3^, Michael Dewaele ^1^, Jean-Christophe Marine ^1^, Patrizia Agostinis^1^

#### ^*1*^*Cell Death and Research Therapy Lab, KU Leuven, Belgium;*^*2*^*Medical University of Innsbruck, Austria;*^*3*^*University of Luxembourg, Luxembourg*

Immunogenic cell death (ICD) is a cell death subroutine associated with the spatiotemporally defined emission/release of damage-associated molecular patterns (DAMPs), which favors the establishment of a productive interface between dying cells and the immune system capable of eliciting effective anti-tumor immunity. Only few ICD-associated DAMPs have been identified so far and include the surface exposure of calreticulin, the active exodus of ATP and passive release of HMBG1 from dying cancer cells. Notably, apoptotic ICD is hallmarked by the rapid induction of an ER stress-mediated danger signaling pathway governed by the PERK-eIF2a arm of the unfolded protein response (UPR). Yet the knowledge of the molecular signature underlying ICD induction and the signaling mechanisms differentiating apoptotic ICD from tolerogenic apoptosis, remain largely incomplete. In this study, we dissected at the molecular and in vivo levels the signaling pathways underlying ICD upon treatment with different ICD inducers (e.g. mitoxantrone and hypericin-based photodynamic therapy) compared to a relative non-ICD inducer (e.g. cisplatin) with the final aim of identifying novel molecular features able to discriminate immunogenic from tolerogenic cancer cell death. We performed RNAseq analysis to identify the genetic profile of melanoma cells dying in response to ICD and non-ICD inducers, followed by in vitro and in vivo (prophylactic vaccination) pathway validation. We show that ICD is hallmarked by an inflammatory transcriptional signature, which closely interconnects with the genetic program elicited by the UPR and cross-talks with the trafficking mechanisms prompting the emission of danger signals. Beyond the role of the PERK arm of the UPR in the trafficking and exodus of critical DAMPs, we found that the IRE1α-XBP1 pathway contributes to ICD by a transcriptional proinflammatory response, thus unravelling an unprecedented role of IRE1α-XBP1 axis in ICD. In conclusion our study underscores the decision-making role of cancer cell autonomous ER stress pathways in ICD and the role of PERK and IRE1a pathways in turning tolerogenic apoptosis into an immunogenic cancer cell death.

## ECDO 88 Physiological role of linear ubiquitination: more to it than meets the eye

### Nieves Peltzer^1^, Maurice Darding^1^, Antonella Montinaro^1^, Peter Draber^1^, Helena Draberova^1^, Sebastian Kupka^1^, Eva Rieser^1^, Lucia Taraborrelli^1^, Amanda Fisher^2^, Tobias L. Haas^3^, Yutaka Shimizu^1^, Aida Sarr^1^, James Rickard^4,5^, Silvia Alvarez-Diaz^4,5^, Ciaran Hutchinson^6^, Charlotta Böiers^7^, Michael T. Ashworth^6^, Allison Beal^1^, Tariq Enver^7^, John Bertin^8^, Philippe Bouillet^4,5^, William Kaiser^2^, Andreas Strasser^4,5^, John Silke^4,5^ and Henning Walczak^1^

#### ^*1*^*UCL Cancer Institute, University College London, 72 Huntley Street, London WC1E 6DD, UK;*^*2*^*University of Texas Health Science Center, 7703 Floyd Curl Drive, San Antonio, TX 78229, USA;*^*3*^*Università Cattolica del Sacro Cuore and Fondazione Policlinico Universitario A. Gemelli, 00168, Rome, Italy;*^*4*^*The Walter and Eliza Hall Institute of Medical Research, 1G Royal Parade, Parkville, Victoria 3052, Australia;*^*5*^*University of Melbourne, Parkville, Victoria 3052, Australia;*^*6*^*Great Ormond Street Hospital & UCL Great Ormond Street Institute of Child Health, London, UK;*^*7*^*Stem Cell Laboratory, UCL Cancer Institute, University College London, London W1CE 6BT, UK;*^*8*^*GlaxoSmithKline, Collegeville, Pennsylvania, USA*

The linear ubiquitin chain assembly complex (LUBAC) is required for optimal gene activation and prevention of cell death upon activation of immune receptors, including TNFR11. Deficiency in the LUBAC components SHARPIN or HOIP in mice results in severe inflammation in adulthood or embryonic lethality, respectively, owing to deregulation of TNFR1-mediated cell death. In humans, deficiency in the third LUBAC component HOIL-1 causes autoimmunity and inflammatory disease, similar to HOIP deficiency. Here we show, by creating HOIL-1-deficient mice, that HOIL-1 is as essential for LUBAC function as HOIP, albeit for different reasons: whereas HOIP is the catalytically active component of LUBAC, HOIL-1 is required for LUBAC assembly, stability and optimal retention in the TNFR1 signalling complex, thereby preventing aberrant cell death. Both HOIL-1 and HOIP prevent embryonic lethality at mid-gestation by interfering with aberrant TNFR1-mediated endothelial cell death, which only partially depends on RIPK1 kinase activity. Co-deletion of caspase-8 with RIPK3 or MLKL prevents cell death in *Hoil-1*^*−/−*^ (also known as *Rbck1*^*−/−*^) embryos, yet only the combined loss of caspase-8 with MLKL results in viable HOIL-1-deficient mice. Notably, triple knockout *Ripk3*^*−/−*^*Casp8*^*−/−*^*Hoil-1*^*−/−*^ embryos die at late gestation owing to haematopoietic defects that are rescued by co-deletion of RIPK1 but not MLKL. Collectively, these results demonstrate that both HOIP and HOIL-1 are essential LUBAC components and are required for embryogenesis by preventing aberrant cell death. Furthermore, they reveal that when LUBAC and caspase-8 are absent, RIPK3 prevents RIPK1 from inducing embryonic lethality by causing defects in fetal haematopoiesis.

## ECDO 89 Studies on photo-sensitivity of a glycol porphyrin derivative and its anti-tumor efficacy

### Sarka Vosahlikova^1^, Irena Moserova^1,2^, Jarmila Kralova^3^, Milan Reinis^4^, Romana Mikyskova^4^, Radek Spisek^1,2^

#### ^*1*^*Sotio, Prague, Czech Republic;*^*2*^*Charles University, 2*^*nd*^*Faculty of Medicine, Prague, Czech Republic;*^*3*^*Institute of Molecular Genetics AS CR, Prague, Czech Republic;*^*4*^*Institute of Molecular Genetics of the AS CR, Prague, Czech Republic*

Photodynamic therapy (PDT) has attracted great attention in cancer treatment. Porphyrin derivative with ethylene glycol chains linked to meta position of meso-tetraphenylporphyrin, mTPP(EG)4, is one of assumed compounds that can be used for treatment of cancer in photodynamic therapy (PDT). The aim of the study is to demonstrate the photodynamic efficacy in vitro and in vivo as well as to analyse its therapeutic mechanism.

The cell viability, cell surface exposure of immunogenic molecules, maturation and phagocytic capacity of PDT-treated tumor cells were monitored by flow cytometry. The kinetics of key components of ER stress-mediated apoptotic pathway was analyzed by western blotting.

Our first results indicate a new and attractive possibility of immunogenic cell death induction. Initiation of immunogenic cell death by PDT was successfully demonstrated in vitro on human and mouse tumor cell lines. The effects of mTPP(EG)4 were compared with cells treated with mTPP(EG)4 without subsequent photoactivation, hypericin-mediated PDT, and high hydrostatic pressure treated cells.

The aim of our following in vivo assays is to evaluate immunogenicity of mTPP (EG) 4 in mice and to assess the ability of PDT treated cells to induce effective immunity against transplanted syngeneic tumors (mouse TRAMP-C2, TC-1 and B16).

The results obtained in this study will provide a basis for possible further preclinical experiments with mTPP(EG)4 or other derivatives, testing their potential in PDT and development of new antitumor therapies.

## ECDO 90 Ubiquitin-mediated regulation of the necroptotic effector MLKL

### Laura Ramos Garcia^1^, Gianmaria Liccardi^1^, Sidonie WickyJohn^1^, Lu Yu^1^, Mercedes Calvo^1^, Jyoti Choudhary^1^, Pascal Meier^1^

#### ^*1*^*Chester Beatty Laboratories, London SW3 6JB, UK*

The ability to escape apoptotic cell death is one of the main characteristics of cancers cells. The pro-caspase, Caspase-8 is mutated in many types of cancers including breast, ovarian and lung. Together with RIPK1, RIPK3 and FADD it forms a cell death-assembling platform, called complex-II, Ripoptosome or Necrosome, which drives apoptotic cell death. Under caspase inhibition or loss of caspase activity, this complex can drive an alternative modality of cell death called Necroptosis. This requires RIPK3 mediated phosphorylation of the pseudokinase MLKL: the final executioner of programmed necrosis characterized by calcium influx and plasma membrane disruption. Given the potentially catastrophic event of spontaneous necroptosis activation, MLKL needs to be tightly regulated. We find that, similarly to RIPK1 and RIPK3, ubiquitylation of MLKL modulates its ability to induce necroptotic cell death. Interestingly, MLKL ubiquitylation correlates with cell death induction in both human and mouse. Furthermore our data indicate that phosphorylated and oligomerised MLKL, required for the latest step of cell death execution, are ubiquitylated, suggesting that ubiquitylation modulates the killing activity of active MLKL. Ongoing work aiming to identify the ubiquitylated lysines on MLKL will shed light into the mechanism of MLKL’s activity modulation via ubiquitylation.

## ECDO 91 Necroptosis may be triggered in human neuroprogenitor cells after zika^br^ infection

### Rafaela Rosa-Ribeiro^1^, Ricardo Weinlich^1^

#### ^*1*^*Hospital Israelita Albert Einstein, São Paulo, Brazil*

When virus enters into the cell, the cell may trigger its own death in an attempt to limit virus replication. Three main types of cell death are involved on virus infection: apoptosis (caspase-dependent), necroptosis (RIPK3 and MLKL-dependent and caspase-independent) and pyroptosis (dependent on inflammatory caspases). During the evolutionary interaction between viruses and cells, different viruses acquired ways to block the cell death pathways. As an example, regarding necroptosis inhibition, herpes virus and cytomegalovirus express proteins that bind to RIPK1 or RIPK3 blocking their interaction, thus hampering the propagation of the necroptotic signaling. It has been previously reported that ZIKA can infect neuroprogenitor cells (NPC), which induces cell death and impairment of 3D neuroesphere formation. Although caspase-3 activation was shown as a consequence of ZIKA infection, which suggests an apoptotic phenotype, studies that thoroughly investigate the different cell death mechanisms involved are still lacking. Trying to elucidate this question, we genetically reprogrammed stem cells (from the tooth pulp of heathy patients) into NPCs. NPCs were then infected with ZIKA^BR^ in different MOI (1, 3, 10) for 1 hour. After this period cells were monitored for 96 hours using live-cell imaging in the presence of a dye that stain dead cells (Sytox Green, Thermo). The MOI 10 of ZIKA^BR^ was the most effective concentration, promoting more than 50% of cell death. Next, in order to check whether NPCs express the necroptosis machinery, qPCRs for RIPK1, RIPK3 and MLKL were performed on uninfected and infected cells (ZIKA^BR^ MOI10). The results showed that the infection increases RIPK3 and MLKL expression in all the time points tested. Then, to explore if ZIKA induces necroptosis in NPCs, four experimental conditions were tested: no treatment; infection with ZIKA^BR^ MOI10 for two hours; NPC treated with 12.5 mM of zVAD; and NPC infected with ZIKA^BR^ for two hours followed by zVAD treatment. Sytox Green quantification by live-imaging showed that, in NPCs, ZIKA^BR^ infection can trigger cell death even when caspases are blocked, suggesting that cells are dying by necroptosis, as both pyroptosis and apoptosis are inhibited by the presence of zVAD. Our results have shown that infected NPC may not only die through caspase activation and apoptosis. These findings raise important questions whether non-apoptotic cell death pathways are involved in the damage caused by ZIKA infection during neural development.

## ECDO 92 Synergistic Interaction between the MCL-1-Specific Inhibitor S63845 and Venetoclax in B-Cell Precursor Acute Lymphoblastic Leukemia

### Felix Seyfried^1^, Felix Stirnweiß^1^, Stefan Köhrer^1^, Klaus-Michael Debatin^1^, Lüder Hinrich Meyer^1^

#### ^*1*^*Ulm University Medical Center, Ulm, Germany*

Deregulated cell death pathways contribute to therapy failure in B-cell precursor acute lymphoblastic leukemia (BCP-ALL) patients. Small molecule inhibitors of key apoptosis regulators have been developed that bind to anti-apoptotic molecules like BCL-2, leading to release of pro-apoptotic proteins and cell death induction. In particular, the BCL-2-specific inhibitor venetoclax (VEN) has demonstrated substantial anti-cancer activity and became an approved drug for the treatment of CLL patients. However, other pro-survival BCL-2 family proteins including MCL-1 mediate VEN resistance. Here, we evaluated the activity of a novel BH3-mimetic, S63845 (S) that selectively targets MCL-1, and addressed potential synergism of simultaneous BCL-2 and MCL-1 blockage by VEN and S in BCP-ALL.

We analyzed the activity of the MCL-1 inhibitor in BCP-ALL cell lines (N=6) and patient-derived primograft samples (N=27) determining half-maximal effective concentrations (EC_50_) by cell viability assays. We found heterogeneous sensitivities to S with EC_50_ values ranging from 16 nM to almost 10 µM. Protein levels of MCL-1, BCL-2, BCL-XL and BCL-W were not associated with S sensitivity in leukemia cell lines nor in primograft samples. Moreover, we also compared sensitivities of both inhibitors but found independent activities of S and VEN in individual ALL samples.

Next, we generated two MCL-1 knock out BCP-ALL cell lines by CRISPR/Cas9 gene editing and found clearly increased VEN sensitivities upon depletion of MCL-1, indicating that MCL-1 at least partially counteracts the activity of VEN. Based on these findings, we investigated the effects of pharmacological MCL-1 inhibition and evaluated the combinatorial effects of S and VEN. Importantly, dual blockage resulted in clear synergistic effects in all 6 BCP-ALL cell lines and 7 primografts (combination indices < 0.3).

Moreover, we also addressed the anti-leukemia activity of combined BCL-2 and MCL-1 inhibition in vivo and treated NOD/SCID mice bearing a high-risk leukemia derived from an infant, MLL/ENL rearranged pro-B ALL with VEN, S, the combination of both, or vehicle for 10 days. Most importantly, significantly reduced loads were found in the co-treated group as compared to vehicle, VEN or S alone in spleen, bone marrow, and central nervous system (p-values < 0.05).

Taken together, our data show heterogeneous activity of S63845 in individual BCP-ALL samples, which is not associated with MCL-1 protein levels or VEN sensitivity. Both, genetic depletion and inhibition of MCL-1 synergize with VEN inducing increased anti-leukemia activity in vitro. Importantly, co-targeting BCL-2 and MCL-1 significantly reduced leukemia loads in a pre-clinical model of high-risk BCP-ALL, warranting further evaluation and possible clinical application of targeting MCL-1 alone and in combination with BCL-2 inhibition.

## ECDO 93 Dinaciclib broadly sensitizes cancer cell to TRAIL-induced apoptosis and overcomes chemoresistance

### Johannes Lemke^1,2^, Antonella Montinaro^2^, Anna-Laura Kretz^1^, Silvia von Kartstedt^2^, Doris Henne-Bruns^1^, Henning Walczak^2^

#### ^*1*^*University of Ulm, Germany;*^*2*^*University College London, London, UK*

Resistance to conventional radio- and chemotherapy is a major obstacle in successfully treating cancer. Therefore, novel effective and cancer-selective therapeutic strategies are urgently needed. Recently, we identified the combination of the death ligand TRAIL and CDK9 inhibition as an exceptional potent strategy to selectively kill tumor cells. Here, we evaluated the combination of the clinically established CDK9-inhibitor Dinaciclib in combination with TRAIL as a novel therapeutic strategy in vitro and in vivo.

Intriguingly, nanomolar concentrations of Dinaciclib drastically sensitizes numerous cell lines of different tumor entities to TRAIL-induced apoptosis and, importantly, almost completely abolished clonogenic survival. Mechanistically, the combination induced cell cycle arrest and caspase-8-dependent apoptosis in these cells by the shift of the ratio of pro- and anti-apoptotic. Intriguingly, Dinacilcib also potently sensitized to TRAIL-induced apoptosis in pancreatic cancer organoids and primary human pancreatic cancer cells. Moreover, Dinaciclib and TRAIL exhibited a potent therapeutic activity in different (orthotopic) cancer mouse models without causing toxicitiy in vivo. Finally, this novel combination was still highly effective in cancer cells, which have acquired resistance to conventional chemotherapy.

In conclusion, due to its potency, CDK9-inhibition in combination with TRAIL provides a novel and promising therapeutic approach for chemoresistant cancers.

## ECDO 94 Induction of caspase-dependent chromatin disassembly in DFF40/CAD-deficient glioblastoma-derived cells

### Laura Martínez-Escardó^1^, María Sánchez-Osuna^1^, Sarah Besora^2^, Jordi Bruna^2^, Victor J. Yuste^1^

#### ^*1*^*Universitat Autònoma de Barcelona, Campus de Bellaterra, 08193 Cerdanyola del Vallès, Barcelona, Spain;*^*2*^*Hospital Universitari de Bellvitge & Institut Català d'Oncologia (ICO) Duran i Reynals, 08907 L’Hospitalet de Llobregat, Barcelona, Spain*

Apoptosis, a tightly regulated cell death program, involves a caspase-triggered cascade of biochemical and morphological events. The activation of Caspase-Activated DNase (CAD), or DFF40, is considered to be the key event allowing dying cells to show classical apoptotic nuclear changes. Apoptotic nuclear morphology is characterized by chromatin condensation into highly packaged round masses and fragmentation of the nucleus. We have previously reported that human glioblastoma (GBM)-derived cells, an extremely aggressive solid brain tumor, holds an intrinsic defect in DFF40/CAD expression^1^. Thus, the natural behavior of GBM cells when challenged with different cytotoxic compounds is to show a unique mass of compacted chromatin in the absence of nuclear fragmentation^2^. However, H&E-stained histological sections from GBM-affected patients revealed several malignant cells showing spontaneous condensation and fragmentation of the nucleus. In this sense, GBM-cultured cells switched its predominant nuclear aspect to a DFF40/CAD-dependent apoptotic nuclear morphology upon an adequate activation of caspases. Therefore, besides GBM cells are deficient in DFF40/CAD expression, the endonuclease can be efficiently activated.

Disclosure: This work was supported by a grant from “Ministerio de Ciencia, Innovación y Universidades”/”Fondo Europeo de Desarrollo Regional” (SAF2017-83206-R) and “Agència de Gestió d’Ajuts Universitaris i de Recerca” (Generalitat de Catalunya) (2017-SGR-1780).

## ECDO 95 Therapy resistance: Do Cancer-Associated Fibroblasts Contribute?

### Anna-Mart Engelbrecht^1^, Carla Fourie^1^, Megan Mitchell^1^

#### ^*1*^*Stellenbosch University, Stellenbosch, 7600, South Africa*

Cancer-associated fibroblasts constitute the most abundant mesenchymal cell type present within the tumour microenvironment. Recent evidence suggests that exposure of stromal fibroblasts to nutrient deprived cancer cells leads to their metabolic reprogramming which may be exploited for cancer cell survival which may contribute to their unresponsiveness or resistance to chemotherapy. Apoptotic or senescent fibroblasts in the tumour microenvironment can secrete a variety of bioactive molecules which promotes tumour growth, metastasis and drug resistance. The mechanisms underlying the interactions between epithelial cancer cells and surrounding stromal fibroblasts remain to be elucidated when treated with doxorubicin.

Proteomic analysis of glucose deprived conditioned media from E0771 cancer cells revealed an increased clustering of proteins involved in epithelial-to-mesenchymal transition and glucose metabolism. MEF cells exposed to E0771 conditioned media exhibited increased oxidative stress levels resulting in increased HIF-1α protein expression. Additionally, enhanced glucose uptake in combination with an increase in GLUT4 translocation was also observed. Our data suggest that glucose deprivation in the E0771 cells does indeed induce the “activation” of a CAF-like phenotype in MEF cells, and this “activation” is associated with extensive metabolic reprogramming. Furthermore, our results also indicate that senescent fibroblasts are able to significantly increase cell viability in E0771 cells following treatment with Doxorubicin. We have demonstrated that healthy, stromal fibroblasts are affected by chemotherapeutic agents to secrete paracrine factors that enhance breast cancer growth and therapeutic resistance.

## ECDO 96 Metabolo-epigenetic superposition of IDH1 and TP53 mutations and cell fates in gliomas

### Alexander Kagansky^1^, Valeriia Gulaia^1^, Dmitry Guschin^2^

#### ^*1*^*Far East Federal University, Vladivostok, Russia;*^*2*^*Institute for Basic Science, Daejeon, Korea*

Most frequent genetic alterations in gliomas are isocitrate dehydrogenase 1 (IDH1) mutations (of arginine 132) and gain-of-function mutations of TP53 (TP53-GOF). Both IDH1 and TP53-GOF mutations heavily impact upon metabolic pathways essential for the cell proliferation, epigenetics, gene expression, apoptosis, and cell senescence in the corresponding tumours. Remarkably these somatic mutations are often co-found in the same tumours and therefore in order to develop effective approaches to their treatment, it is crucial to understand the affected biological processes. Thus to decipher the impact of these aberrations on the tumour biology, we need to understand how these key mutations alter key pathways contributing to the disease phenotype and to isolate the advantages that their cooperation brings in to facilitate tumour development and evade the existing treatments. Our approach is to examine the molecular consequences of both mutations introduced in glioma cell culture individually and together using genome editing.

## ECDO 97 Altered polarisation status of OPA1-deficient macrophages mediated by metabolic rewiring of macrophages

### Nikita Markov^1^, Darko Stojkov^1^, Shida Yousefi^1^, Hans-Uwe Simon^1^

#### ^*1*^*University of Bern, CH-3010 Bern, Switzerland*

Mitochondria are well known for their role as bioenergetic and biosynthetic organelles. They have emerged as a crucial component of many cellular systems and pathways. Unsurprisingly, mitochondria play an important role in the immune system. Optic Atrophy 1 (OPA1) is a mitochondrial inner membrane protein known for its role in mitochondrial fusion and morphology. Interestingly, and independent of its role in mitochondrial fusion, OPA1 is also responsible for maintaining the mitochondrial cristae junctions tight, allowing efficient levels of oxidative respiration. We have recently reported that lack of OPA1 leads to defect in neutrophil extracellular DNA (NETs) formation, an essential innate immune function.

Macrophages are the key component of immune system located at the crossroads of Th1 and Th2 immune responses. Macrophage polarization is a process by which macrophages exhibit different functional programs in response to microenvironmental signals. Following IFN-γ signalling and TLRs activation macrophages undergo M1 polarization which represents classical activation characterised by proinflammatory response. Alternatively, M2 macrophages are found in conditions dominated by Th2 response and induced by IL-4/IL-13/IL-10 signalling, thereby acquiring anti-inflammatory and tissue remodelling properties.

Intriguingly, differently polarized macrophages rely on different sources of energy. M2 macrophages exhibit increased flux through oxidative phosphorylation (OXPHOS) and M1 macrophages rely on glycolysis for the energy production. Therefore, metabolic rewiring of polarized macrophages could become a new therapeutic strategy to threat pathologies that have a high macrophage involvement.

Our preliminary data indicates that M0/M2 polarized OPA1-deficient macrophages exhibit metabolic profile, which is similar to M1 wild type macrophages. Mitochondrial dysfunction in M0/M2 OPA1-deficient macrophages drastically downregulates the level of oxidative phosphorylation and NAD^+^/NADH ratio and compensatory upregulation of glycolysis flux, mimicking M1 wild type macrophages. Moreover, mitochondrial dysfunction and energy crisis caused by the drop of ATP level entirely alters activation of many energy/stress sensing kinases such as AMPK, p38 and AKT/mTOR pathway. Most surprisingly, despite the mitochondrial dysfunction, we observed a major upregulation of key markers of M2 polarization such as Arg1, Ym1 and Cdh1.

Collectively, these findings provide novel insights on the interplay between macrophage polarization and mitochondrial dysfunction, which could be possibly serving as a basis for future therapeutic approaches.

## ECDO 98 How can a milk protein selectively kill cancer cells? Mechanisms underlying lactoferrin-induced apoptosis

### Cátia S. Pereira^1^, Joana P. Guedes^1^, Hernâni Gerós^1^, Lígia R. Rodrigues^1^, Manuela Côrte-Real^1^

#### ^*1*^*University of Minho, Braga, Portugal*

Lactoferrin (Lf) is an iron-binding protein abundant in milk that has been shown to exhibit anticancer activity. Since Lf is non-toxic to cancer cells and is well tolerated in humans, this protein has a huge potential to be used in cancer therapy. However, the targets and mechanisms underlying its selective anticancer activity are poorly elucidated, which limits its clinical exploitation. The recruitment of the proton pump V-ATPase to the plasma membrane, where it mediates the acidification of the tumor microenvironment, is a recognized feature involved in the acquisition of a metastatic phenotype in different cancers, including breast cancer. Therefore, inhibitors of this pump have emerged as promising anticancer drugs. Here we showed that bovine lactoferrin (bLf) preferentially inhibits cell proliferation and induces apoptosis in two highly metastatic breast cancer cell lines, which display a prominent localization of V-ATPase at the plasma membrane, but not in a lowly metastatic or a non-tumorigenic cell lines. We then characterized the mechanism underlying bLf-induced apoptosis and demonstrated that bLf selective cytotoxicity is caused by the inhibition of the extracellular acidification rate and the ensuing intracellular acidification in the highly metastatic breast cancer cells. Accordingly, bLf, like the well-known proton pump inhibitors concanamycin A and bafilomycin A1, inhibits V-ATPase proton pumping and hydrolytic activities in sub-cellular fractions enriched in this proton pump. We recently also demonstrated that bLf preferentially induces apoptosis in other types of highly metastatic cancer cells other than breast. Altogether, our data demonstrated for the first time that bLf acts as a V-ATPase inhibitor and established a common mechanism of action of bLf against highly metastatic cancer cell exhibiting this proton pump at the plasma membrane. This study opens promising perspectives for the safer and more rational application of bLf in the therapy of these life-threatening cancers.

## ECDO 99. Inorganic Polyphosphate Induces Necrosis of Glial Cells and Modulates Electrophysiological Activity of Neurons

### Neginskaya M.A.^1^, Berezhnaya E.V.^1^

#### ^*1*^*Academy of Biology and Biotechnology, Southern Federal University, Rostov-on-Don, Russia*

Inorganic polyphosphate (PolyP) is a linear biopolymer composed of tens to hundreds orthophosphate residues linked by the high energy phosphoanhydride bonds. PolyP is found in all living organisms and plays numerous physiological functions. In mammals PolyP is involved in the regulation of Ca^2+^ uptake in mitochondria, bone tissue development, and blood coagulation. Micromolar concentrations of PolyP is also known to present in a mammalian brain. However, the role of PolyP in neurons and glial cells is unclear. In this study we explore physiological reactions of neurons and glial cells on exogenous inorganic PolyP with different lengths (14 and 130 residues).

Crayfish stretch receptor (CSR) consisting of only two receptor neurons surrounding by glial cell was used as an object. Extracellular recordings were used to observe electrophysiological reactions of neurons to PolyP. Cell death was controlled with the aid of fluorescent dyes Propidium Iodide and Hoechst 33342. Calcium indicator Fluo-4 AM was used to study PolyP-induced changes in concentration of cytosolic calcium.

It was shown that long (100 µM) but not short (100 µM) PolyP induces temporary increase of action potentials frequency of neurons. Long PolyP also increased the level of necrosis of surrounding glial cells and reduced the level of spontaneous glia apoptosis. Pre-incubation of CSR with inhibitor of P2 purinoceptors PPADS (100 µM) protected glial cells from PolyP-induced necrosis but did not influence the level of glia apoptosis in presence of long PolyP or the electrophysiological reactions of neurons on PolyP. It was interesting that the increase of cytosolic calcium in neurons and surrounding glial cells was stimulated not only by long PolyP but also by short PolyP addition.

The data obtained suggests that long PolyP can trigger the activation of signaling pathways that is involved in regulation of necrosis and apoptosis in glial cells. Long PolyP induced necrosis via activation of purinergic receptors as PPADS abolished its toxic effect. But in neurons electrophysiological reactions on long PolyP was probably connected with membrane depolarization and activation of voltage dependent Ca^2+^ channels due to negative charge of PolyP rather than with activation of ATP receptors of plasma membrane.

Disclosure: The study was supported by Russian Foundation for Basic Research (project No. 17-04-01728). Neginskaya M. was supported by the stipend of the President of Russian Federation for young researches.

## ECDO 100. Analysis of epigenetic perturbations in gliomas

### Alexander Kagansky^1^, Valeriia Gulaia^1^, Vladlena Tiasto^1^, Valeriia Mikhailova^1^, Hazel Thoms^2^, Joshua Burton^3^, Andrey Yurchenko^4^, Dmitry Guschin^5^, Paul Brennan^6^, Artur Biktimirov^7^, and Vladimir Teif^3^

#### ^*1*^*Center for Genomic and Regenerative Medicine, School of Biomedicine, Far East Federal University, Vladivostok, Russia;*^*2*^*MRC Human Genetics Unit, University of Edinburgh, Edinburgh, UK;*^*3*^*School of Biological Sciences, University of Essex, Colchester, Essex, UK;*^*4*^*Institut de Cancérologie Gustave Roussy, Villejuif, France;*^*5*^*Center for Genome Engineering, Institute for Basic Science, Daejeon, Korea;*^*6*^*MRC Centre for Regenerative Medicine, University of Edinburgh, Edinburgh, UK;*^*7*^*FEFU Medical Centre, School of Biomedicine, Far East Federal University, Vladivostok, Russia*

Glioblastoma multiforme (GBM) is a rapidly growing malignant tumour that occurs in either the brain or the central nervous system of patients. It has a low survival rate and few means of treatment with varying degrees of success. Most frequent genetic alterations in gliomas are isocitrate dehydrogenase 1 (IDH1) mutations (of arginine 132) and gain-of-function mutations of TP53 (TP53-GOF). Both IDH1 and TP53-GOF mutations heavily impact upon metabolic pathways essential for the cell proliferation, epigenetics, gene expression, apoptosis, and cell senescence in the corresponding tumours. Remarkably these somatic mutations are often co-found in the same tumours and therefore in order to develop effective approaches to their treatment, it is crucial to understand the affected biological processes. Our work is aimed at looking further into epigenetic changes in glioblastoma, more specifically chromatin changes and histone modifications. Six glioblastoma samples and 6 brain control samples obtained from patients were analysed by RNA-Seq. The resulting data was analysed for known single nucleotide variants, DNA repeat reads, and differential gene expression. Gene ontology analysis of the differential expression data highlighted alterations in several key pathways, including p53 pathway. To decipher the contributions of IDH1 and TP53-GOF alterations to the tumour biology, we need to understand how these mutations alter key pathways contributing to the disease phenotype separately and in combination. This will allow to determine the hypothetic advantages that their cooperation brings in to facilitate tumour development and evade the existing treatments. Therefore our further aim is to introduce these mutations via CRISPR-Cas and to examine the molecular consequences of both mutations introduced in glioma cell culture individually and together.

